# Primary versus castration-resistant prostate cancer: modeling through novel murine prostate cancer cell lines

**DOI:** 10.18632/oncotarget.8436

**Published:** 2016-03-28

**Authors:** Georges Daoud, Alissar Monzer, Hisham Bahmad, Farah Chamaa, Layal Hamdar, Tarek H. Mouhieddine, Sami Shayya, Assaad Eid, Firas Kobeissy, Yen-Nien Liu, Wassim Abou-Kheir

**Affiliations:** ^1^ Department of Anatomy, Cell Biology and Physiological Sciences, Faculty of Medicine, American University of Beirut, Beirut, Lebanon; ^2^ Department of Biochemistry and Molecular Genetics, Faculty of Medicine, American University of Beirut, Beirut, Lebanon; ^3^ Graduate Institute of Cancer Biology and Drug Discovery, College of Medical Science and Technology, Taipei Medical University, Taipei, Taiwan

**Keywords:** prostate cancer, castration-resistant prostate cancer, cancer stem cells, Pten, TP53

## Abstract

Cell lines representing the progression of prostate cancer (PC) from an androgen-dependent to an androgen-independent state are scarce. In this study, we used previously characterized prostate luminal epithelial cell line (Plum), under androgen influence, to establish cellular models of PC progression. Cells derived from orthotopic tumors have been isolated to develop an androgen-dependent (PLum-AD) versus an androgen-independent (PLum-AI) model. Upon immunofluorescent, qRT-PCR and Western blot analyses, PLum-AD cells mostly expressed prostate epithelial markers while PLum-AI cells expressed mesenchymal cell markers. Interestingly, both cell lines maintained a population of stem/progenitor cells. Furthermore, our data suggest that both cell lines are tumorigenic; PLum-AD resulted in an adenocarcinoma whereas PLum-AI resulted in a sarcomatoid carcinoma when transplanted subcutaneously in NOD-SCID mice. Finally, gene expression profiles showed enrichment in functions involved in cell migration, apoptosis, as well as neoplasm invasiveness and metastasis in PLum-AI cells. In conclusion, these data suggest that the newly isolated cell lines represent a new *in vitro* model of androgen-dependent and –independent PC.

## INTRODUCTION

Prostate cancer (PC) is the most commonly diagnosed cancer among men in the United States [1], and is the second most leading cause of cancer-related deaths in men worldwide [2]. Relationship between androgens and PC has established the basis for the current treatment of androgen deprivation in advanced PC [3], where several studies have shown that androgens promote PC cell growth through different mechanisms, such as induction of autophagy by androgen-mediated increases in reactive oxygen species [4] or targeting of rapamycin (mTOR) activation and post-transcriptional increases in cyclin D proteins [5]. However, the effect of hormone therapy is temporary, and most tumors become resistant to androgen deprivation therapy (ADT) within few years [6]. Eventually, many patients die of metastatic castration-resistant prostate cancer (CRPC), also known as androgen-independent prostate cancer (AIPC). Although new anti-androgen therapies effectively alleviate symptoms and prolong life, there are still no curable treatments for CRPC [6]. Several clinical trials have been carried out to assess different therapeutic modalities for treating CRPC, but the results have not been encouraging [7, 8]. Since drugs such as abiraterone, enzalutamide and TOK-001 are failing and immunotherapy and bone-targeted therapies such as bisphosphonates, denosumab and Radium-223 are not very efficient [9], other attempts are heading towards gene-targeted therapies such as delivering apoptotic genes (like BikDD) through a vector-liposome complex to induce apoptosis in PC cells [10]. This gene-targeted therapy still needs further validation and testing before we can reach an effective treatment for PC in general and CRPC in particular. Thus, understanding the mechanistic basis that underlies the genesis of CRPC and PC metastasis is fundamental for defining appropriate targets for treatment and prophylactic therapies.

It has been proposed that prostate tumors arise from a small population of androgen-independent cells, often presumed to be androgen-independent prostate stem cells. During androgen-dependent development, the androgen receptor (AR) acts as the primary mediator of growth and survival of PC cells. Later, and upon androgen-independent progression, cancer cells tend to develop a variety of cellular pathways to flourish in an androgen-depleted environment, via different mechanisms including AR gene amplification, AR gene mutations, involvement of coregulators, ligand independent activation of the AR, and the involvement of tumor stem cells [11].

The transition from primary to metastatic PC is initiated by a mechanism called epithelial-to-mesenchymal transition (EMT) [12]. Studies have shown that expression levels of AR are inversely correlated with androgen-mediated EMT in PC epithelial cells, suggesting a low AR content required for the EMT phenotype [13]. Interestingly, recent studies have found that CRPC cells may acquire their independency from androgens via differential expression of androgen receptor cofactors [14], splice variants [15], and other genetic alterations [16].

The effect of androgen deprivation on the progression of PC is still poorly understood. This is mainly due to the scarcity of *in vitro* cell models recapitulating disease progression. We have recently generated a novel murine *in vitro* system, namely PLum cells, which recapitulated, to some extent, the disease progression upon ADT conditions [17]. In the current study, we examined the molecular, functional, and pathophysiological differences between two novel murine PC cell lines that were derived from androgen-dependent (PLum-AD) and androgen-independent (PLum-AI) PC, both of which harbor the same genetic background (*Pten-/−TP53-/−*) [17]. Our results show that PLum-AI cells express an aggressive nature that may reflect the castration-resistant stage of PC as compared to PLum-AD cells which represent the primary stage.

## RESULTS

### Characterization of the morphology and cell markers expression of PLum-AD and PLum-AI cells

**Figure 1:**
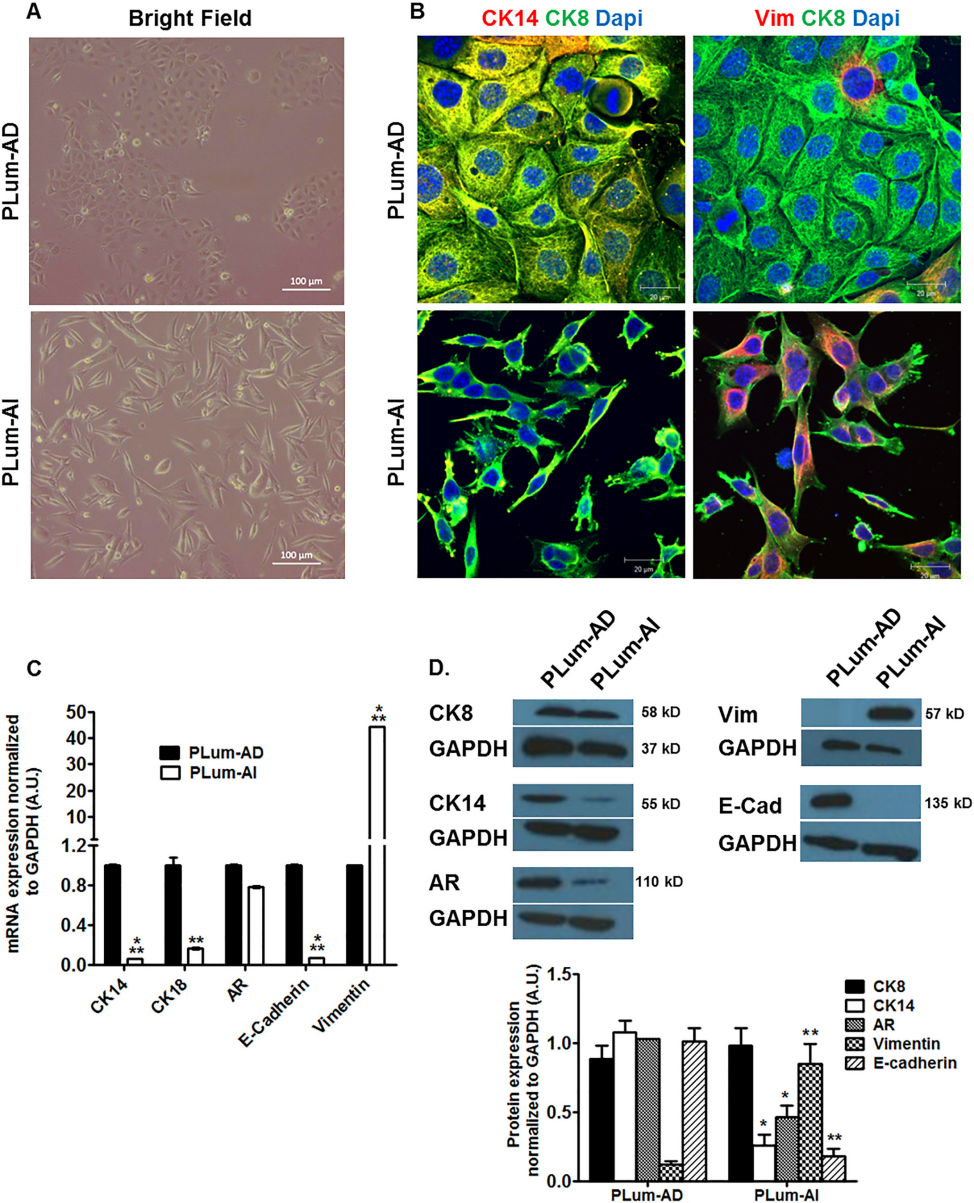
Lineage characterizations of PLum-AD and PLum-AI cell lines (**A**) Representative bright-field images of PLum-AD and PLum-AI cells. Scale bar = 100 μm. (**B**) Representative immunofluorescent images of PLum-AD and PLum-AI cells stained for the indicated antibodies and the nuclear counterstain Dapi are shown. Scale bars = 20 μm. (**C**) Expression of different prostate epithelial lineages and mesenchymal cell markers determined using qRT-PCR analysis. Values were normalized to GAPDH and the data were plotted relative to PLum-AD. The data are reported as mean ± SD (**P < 0.01 and ***P < 0.001; Plum-AI compared to PLum-AD cells, *t*-test). (**D**) Protein expression of different prostate epithelial and mesenchymal markers in PLum-AD and PLum-AI cells was analyzed by Western Blotting with the indicated antibodies. A representative blot and quantification of each protein/GAPDH signal intensity ratios are shown, the data are reported as mean ± SEM (*P < 0.05 and **P < 0.01; PLum-AI compared to PLum-AD cells, *t*-test).

In our study, we used orthotopic tumors that were previously generated from parental PLum cells under androgen-dependent or androgen-independent conditions [17] in order to establish cellular models of PC progression. Tumors were then taken and minced into small pieces to obtain PLum-AD and PLum-AI cells. We further sought to determine the characteristics of the newly isolated PLum-AD and PLum-AI murine PC cells. We cultured both PLum-AD and PLum-AI cells in the same media, PrEGM, in which the parental PLum cells were cultured and have been shown to select for their growth [17]. Unlike PLum-AD cells that grew well in serum-free medium, PLum-AI cells grew better in 5% FBS-containing medium and therefore they were maintained in 5% FBS in all experiments. Morphologically, PLum-AD cells isolated from tumors in the presence of androgens, mainly adenocarcinoma, showed a typical epithelial phenotype (cobble-stone appearing cells with well-defined cell-cell interaction), whereas PLum-AI cells isolated from poorly differentiated sarcomatoid carcinoma in the absence of androgen revealed a typical epithelial-to-mesenchymal morphology (migratory phenotype with minimal cell-cell interaction) (Figure 1A). Thus, both cell lines remained identical to the original tumors they were isolated from as regards to their phenotypes [17].

These morphological differences were further confirmed by immunofluorescent staining using lineage specific markers including CK8 (epithelial luminal cell marker), Vimentin (mesenchymal cell marker) and CK14 (intermediate cell marker). While all cells expressed CK8 homogenously, our analysis showed that co-expression of CK14 and CK8 was found in the majority of PLum-AD cells and only in minor populations of PLum-AI cells. Inversely, PLum-AI cells co-expressed vimentin and CK8 while PLum-AD cells showed little co-expression of the same combination (Figure 1B). Given the aggressive nature of prostate sarcomatoid tumor in comparison to adenocarcinoma, these data proved to be consistent with the morphology and expression profile of the original tumors these cells were isolated from.

Moreover, a quantitative reverse transcription-PCR (qRT-PCR) and Western Blot (WB) analyses were performed to characterize the newly described cell lines. mRNA expression levels of several genes involved in prostate lineage differentiation, self-renewal and EMT process showed higher expression of CK14, CK18 and E-cadherin (epithelial markers) levels in PLum-AD cells compared to PLum-AI cells. Inversely, vimentin expression level in PLum-AI cells was very high compared to PLum-AD cells (about 40 times higher) (Figure 1C). WB analysis showed similar results at the protein expression levels consistent with the mRNA expression data confirming the epithelial phenotype of PLum-AD cells compared to the mesenchymal phenotype of PLum-AI cells (Figure 1D). Interestingly, AR protein expression level was higher in PLum-AD cells compared to PLum-AI cells despite no significant change at the mRNA level (Figure 1C and 1D). These data correlate with the hypothesis that PLum-AI cells might represent the CRPC stage of the disease.

### PLum-AD and PLum-AI cells retain stem/progenitor cell properties *in vitro* (self-renewal ability and differentiation plasticity)

**Figure 2:**
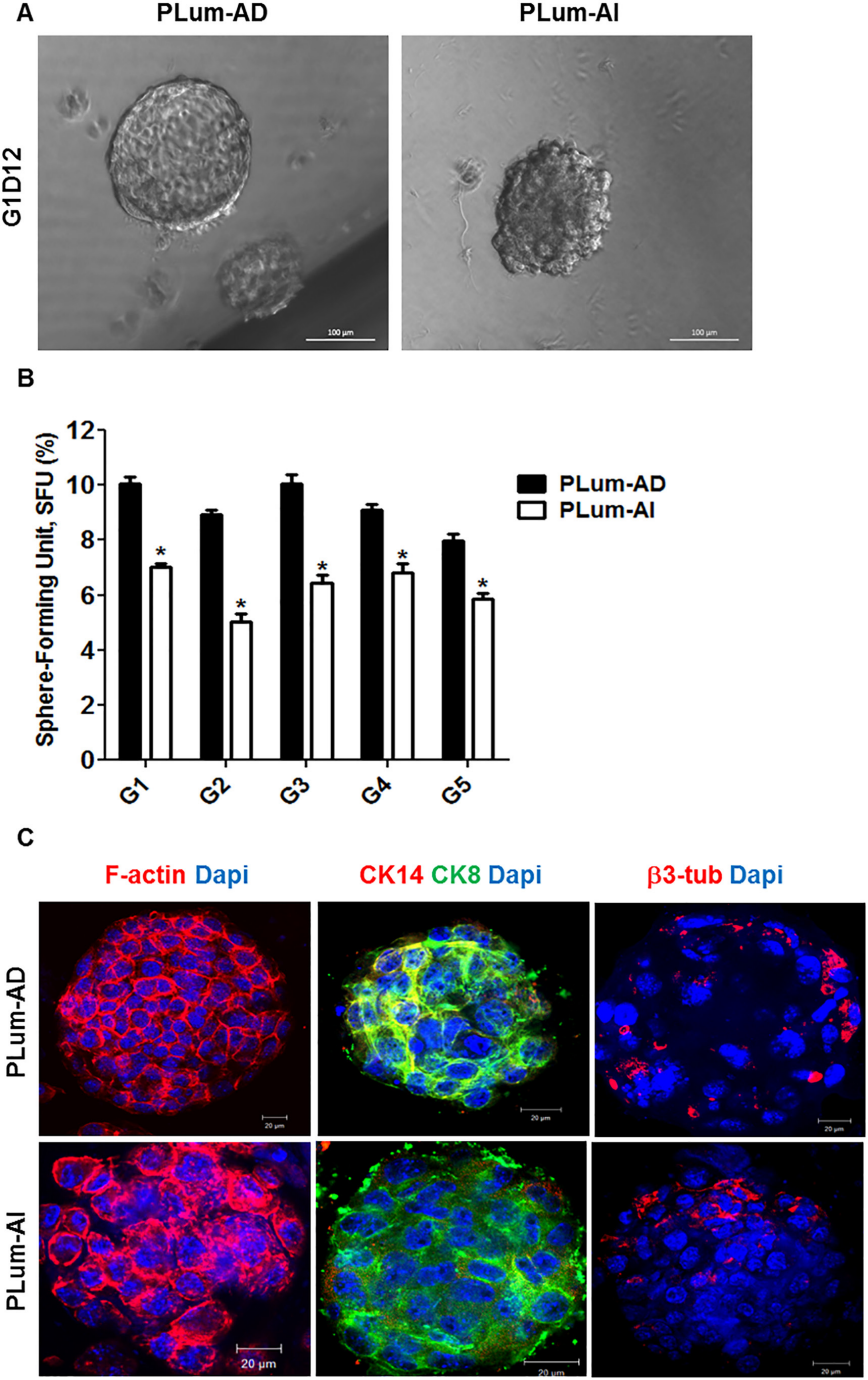
PLum-AD and PLum-AI cell lines display stem-like cell properties (**A**) Representative bright-field images of PLum-AD and PLum-AI protospheres in Matrigel^™^ at G1D12 (Generation 1 Day 12). Scale bar = 100 μm. (**B**) PLum-AD and PLum-AI cells were plated in MatrigelTM at a density of 2,000 cells/well in a 12-well plate for sphere formation assay. Sphere forming units expressed as % of 2,000 input cells at each generation obtained from serially passaged protospheres are shown. The data are reported as mean ± SD (*P < 0.05; PLum-AI compared to PLum-AD cells, *t*-test). (**C**) Representative immunofluorescent images of PLum-AD and PLum-AI protospheres stained for F-actin, CK8, CK14, β3 tubulin and Dapi are shown. Scale bars = 20 μm.

Since the original PLum cells were generated from an enriched population of stem/progenitor cells [17], we sought to evaluate the stem/progenitor cell-like properties of PLum-AD and PLum-AI cells, including capability of self-renewal and differentiation. Sphere formation assay was performed on these cells as it had been previously used for the growth of prostate epithelial stem/progenitor cells *in vitro* [17, 18]. Our results showed that both cell lines formed spheres and therefore contain cells with stem/progenitor characteristics (Figure 2A and 2B). Interestingly, PLum-AD cells formed large regular spheres reflecting their epithelial origin, whereas PLum-AI cells produced irregular spheres that are stellate in shape supporting their mesenchymal phenotype (Figure 2A). These observations point to the aggressiveness of PLum-AI cell line fitting the criteria of CRPC stage of the disease.

To assess the self-renewal ability of these cell lines, spheres were allowed to further propagate for several generations. Remarkably, both cell lines continued to form spheres for 5 generations without losing their sphere-forming capacity, suggesting that they both possess stable self-renewal ability (Figure 2B). Our data showed that the sphere forming unit (SFU) was always higher in PLum-AD cells compared to PLum-AI cells, indicating the presence of more cell populations with stem/progenitor cell-like characteristics (Figure 2B).

Moreover, in order to assess the differentiation potential of both cell lines, we stained PLum-AD and PLum-AI protospheres for structural and lineage markers including F-actin, CK8, CK14 and β3 tubulin. Expression of β3-tubulin was shown to be increased in CRPC and might have a role in the progression of PC [19]. Confocal images through PLum-AD and PLum-AI protospheres are shown in Figure 2C. F-actin staining revealed the architectural organization of the spheres, where PLum-AD spheres showed intact organization while PLum-AI spheres revealed disorganized phenotype. Interestingly, both PLum-AD and PLum-AI spheres contained cells with different differentiation potential as they stained positive for CK8, CK14 (less in PLum-AI) and β3-tubulin (Figure 2C).

### PLum-AI cells demonstrate more migratory and invasive capacity than PLum-AD cells

**Figure 3:**
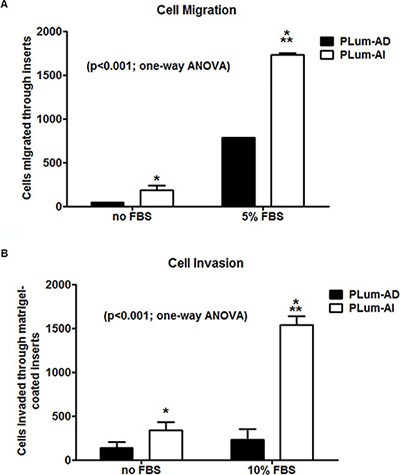
Migration and invasion potentials of PLum-AD and PLum-AI cells PLum-AI cells showed significantly higher migration and invasion potential compared to PLum-AD. Moreover, FBS positively induced migration and invasion in both cell lines. (**A**) The cell migration potential in response to FBS was assessed in PLum-AD and PLum-AI cells using the transwell-migration assay. The data are reported as mean ± SD (*P* < 0.001; One way ANOVA; *P < 0.05 and ***P < 0.001; PLum-AI compared to PLum-AD cells, Tukey's multiple comparison test). (**B**) The cell invasion potential in response to FBS was assessed in PLum-AD and PLum-AI cells using the Matrigel^™^-coated transwell-invasion assay. The data are reported as mean ± SD (*P* < 0.001; One way ANOVA; *P < 0.05 and ***P < 0.001; PLum-AI compared to PLum-AD cells, Tukey's multiple comparison test).

Because invasion of neoplastic cells into adjacent tissues and metastasis into distal organs are typical characteristics of the aggressive CRPC, the migration and invasion abilities of PLum-AD and PLum-AI cells in the presence and absence of the chemoattractant FBS were evaluated. Upon performing the transwell migration and invasion assays, both cell lines were able to migrate and invade in response to FBS, with higher fold induction in PLum-AI cell invasion, confirming basic characteristics of cancer cells in general (Figure 3). Interestingly, in the absence of FBS, PLum-AI cells showed higher basal migratory (Figure 3A) and invasion (Figure 3B) potentials compared to PLum-AD cells, which is consistent with the results obtained previously showing that PLum-AI cells possess an epithelial-to-mesenchymal phenotype and seem to be more aggressive in nature than PLum-AD. The difference in basal migration and invasion potential was maintained and enhanced in response to FBS.

### PLum-AI cells possess higher tumorigenic potential than PLum-AD cells

**Figure 4:**
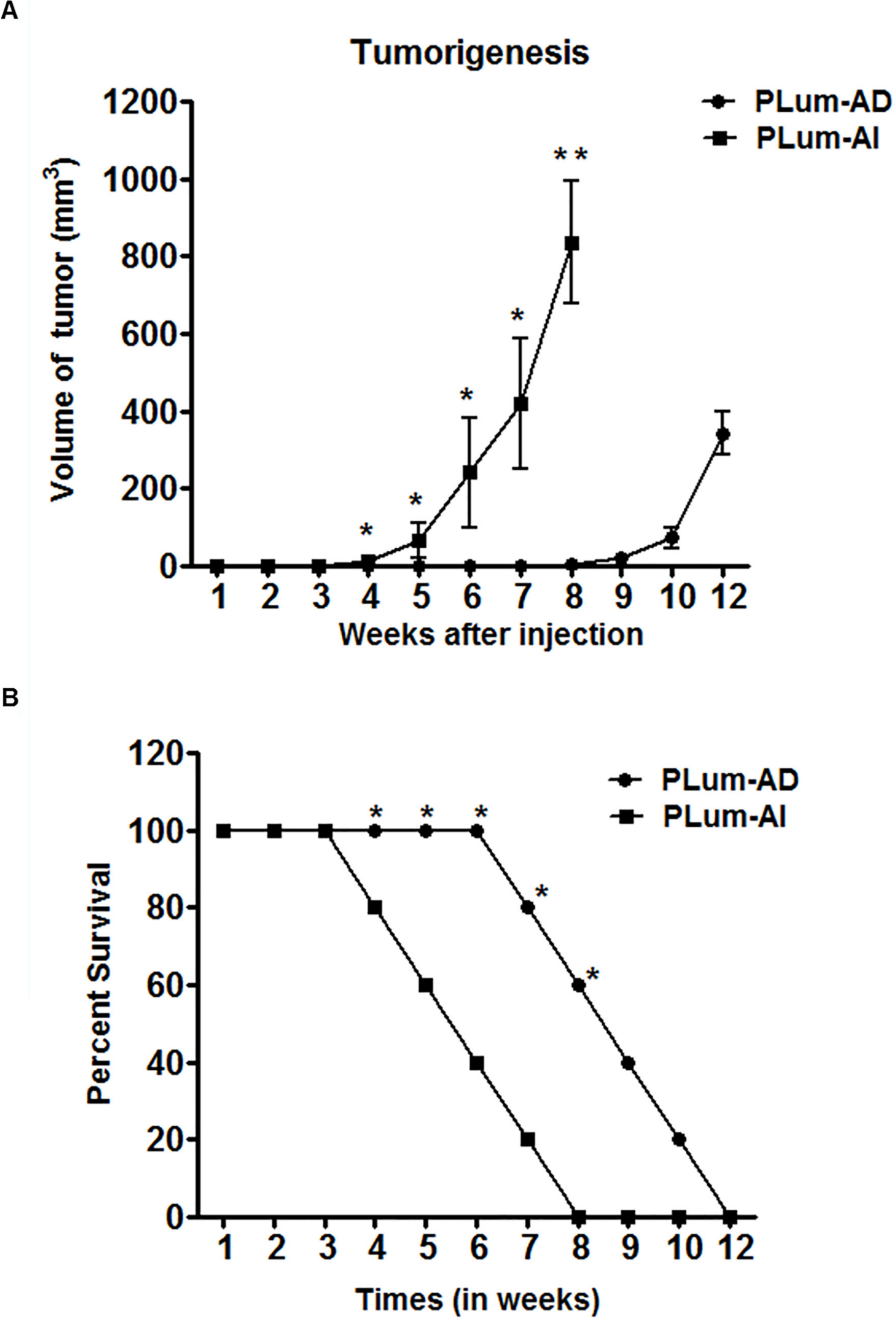
Tumorigenic potential of PLum-AD and PLum-AI cells (**A**) Tumor size measurements in mice that had been injected with PLum-AI cells show development of larger tumor and in less than half the time needed than for those with transplanted PLum-AD cells. Tumor size and expansion were determined by direct physical measurements of the tumors at the primary site of injection, twice per week, until the termination of the experiment. Data represent an average of *n* = 3 mice. The data are reported as mean ± SD (*P < 0.05 and **P < 0.01, Plum-AI compared to PLum-AD cells at that time point, *t*-test). (**B**) Survival analysis. The survival curves of male NOD-SCID mice injected subcutaneously with 1 × 10^6^ PLum-AI (*n* = 15) cells revealed lower survival rate as compared to mice injected with PLum-AD (*n* = 15) cells. The data are reported as mean ± SD (*P < 0.05, PLum-AI compared to PLum-AD cells at that time point, *t*-test).

Since the invasion and migration assays showed more aggressive phenotype in PLum-AI cells as previously mentioned, we further investigated the ability of those cells to form tumors *in vivo*. First, we injected cancer cells subcutaneously into the flanks of 8–10 week old NOD-SCID mice. Once palpable tumor was detected, tumor size was measured regularly twice every week until the time of death. Interestingly, mice that had been transplanted with PLum-AI cells developed tumor in less than half the time that was needed for those with transplanted PLum-AD cells (4 weeks in PLum-AI injected mice versus 8 weeks in PLum-AD injected mice) (Figure 4A). This might be explained by the aggressive phenotype of PLum-AI cells. Additionally, tumors formed by PLum-AI cells were significantly larger in volume than those formed by PLum-AD cells suggesting the more aggressive nature of PLum-AI cells.

The survival rate of mice from the time of injection till death was also documented (Figure 4B). Mice that were injected with PLum-AI cells showed a much less survival rate (all mice were dead by 8 weeks post-transplantation) compared to those injected with PLum-AD cells (all mice were dead by 12 weeks post-transplantation). This is in line with the aggressive behavior of PLum-AI cells.

### Characterization of subcutaneously transplanted tumors initiated from PLum-AD and PLum-AI cells

**Figure 5:**
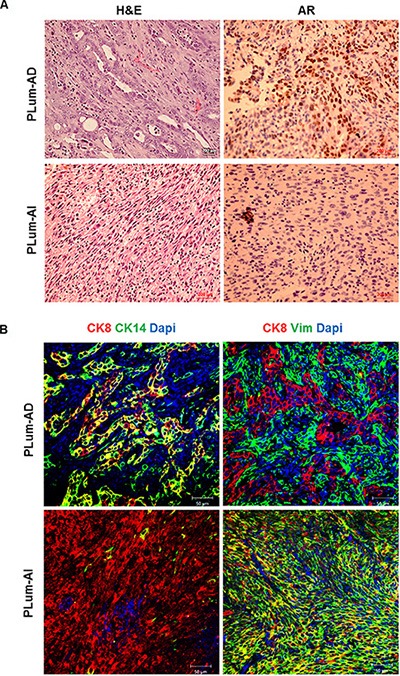
Characterization of subcutaneously transplanted tumors initiated from PLum-AD and PLum-AI cells (**A**) Cross-sections of subcutaneous tumors stained with H & E (left panel) showed typical adenocarcinoma in PLum-AD tumors and sarcomatoid carcinoma in PLum-AI tumors. Immunohistochemical analysis of AR (right panel) showed high expression in PLum-AD tumors and low expression in PLum-AI tumors. Scale bars = 200 μm. (**B**) Cross-sections of PLum-AD and PLum-AI subcutaneous tumors were stained with CK14, CK8, vimentin and Dapi. Scale bars = 50 μm.

Next, we sought to further characterize the tumors initiated from subcutaneous transplantation of both cell lines in mice. After fixation in 4% PFA, the tumors were embedded in paraffin, sectioned, stained with H & E and examined by light microscopy (Figure 5A). The majority of the tumor area formed by subcutaneous transplantation of PLum-AD cells consisted of adenocarcinoma, while PLum-AI cells formed sarcomatoid carcinoma with spindle-shaped tumor cells. Furthermore, immunohistological examination of serial subcutaneous tumor sections stained with AR showed that PLum-AD adenocarcinoma displayed strong homogenous nuclear labeling of AR compared to PLum-AI sarcomatoid carcinoma which showed a low expression for AR (Figure 5A). This is consistent with the tumor phenotypes where adenocarcinomas typically express AR while sarcomatoid carcinomas lose the AR expression. Moreover, tumor sections were stained for CK8, CK14 and Vimentin. Our results revealed that tumors formed by PLum-AD cells expressed CK8 in addition to some cells co-expressing CK8/CK14. This is consistent with the glandular phenotype of adenocarcinomas. Tumors generated by PLum-AI cells also showed CK8 expression but with a major loss of CK14 expression (Figure 5B). Interestingly, Vimentin expression, a marker of EMT, was more prominent in tumors generated by PLum-AI cells compared to PLum-AD cells (Figure 5B). This is consistent with the nature of the two tumors and correlates with the hypothesis that PLum-AI cells might represent the CRPC stage of the disease. All these data indicate that the isolated cell lines are tumorigenic in nature and tumors resemble the original tumors they were isolated from and the phenotype of the cells *in vitro*.

### Differential gene expression profile between PLum-AD and PLum-AI cells

**Figure 6:**
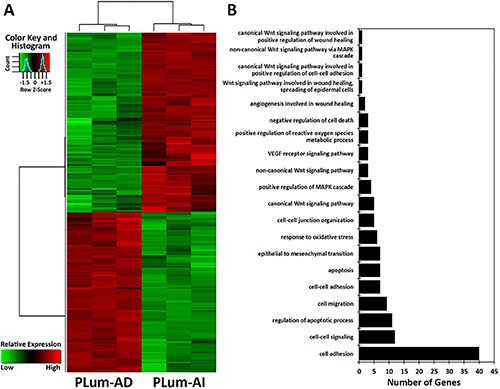
Microarray analysis of the differentially expressed genes between PLum-AD and PLum-AI cells (**A**) Hierarchical clustering analysis of gene expression profiles. Each column represents one sample, and each horizontal line refers to a gene. Color legend is on the top-left of the figure. Red indicates genes with a greater expression relative to the geometrical means; green indicates genes with a lower expression relative to the geometrical means. (**B**) Biological process Gene Ontology (GO) analysis of the differentially expressed genes, classified into 20 categories, many of which shared the same genes, according to their functional correlation.

In the present study, we utilized a genomics microarray analysis to identify differences in the gene expression pattern in PLum-AD and PLum-AI cells. A global gene expression profile of both cell lines was performed on 6 samples (3 PLum-AD, and 3 PLum-AI). Of interest, gene expression analysis revealed 382 transcripts with differential expression of 4 fold change (FC) or greater, and significance of less than 0.05. Among these, 210 were upregulated while 172 genes were shown to be downregulated in PLum-AI cells compared to PLum-AD cells (Figure 6A and Supplementary Table S1). Based on functional gene ontology (GO) annotation analysis, gene clusters were classified into 20 categories, many of the genes were shared among the different ontologies as these can be performing 2 or more specific biological function (Figure 6B and Supplementary Table S2). Analysis of the specific enriched pathways revealed several altered genes associated with biological pathways including: cell-cell interaction, cell death, cell migration, response to oxidative stress, EMT and Wnt signaling pathways as illustrated in Figure 6B.

Among the genes related to cell migration that were identified in PLum-AI cells, six genes (*FLT1, PDGFRA, ITGA7, TGFBR3, MMP2, FMNL3*) were upregulated and three genes (*CTGF, THBS1, ST14*) were downregulated, all compared to PLum-AD cells (Supplementary Table S3). In addition, several genes related to the response to oxidative stress (*NQO1, SOD3, MMP2, LCN2, TXNIP, and DGKK*) were differentially expressed in PLum-AI cells (Supplementary Table S3). Importantly, *MMP2* gene, encoding a proteolytic enzyme, matrix metalloproteinase 2 (MMP2 enzyme), which is shown to be involved in the invasion and metastasis of PC [20], was significantly upregulated in PLum-AI cells as it displayed 33-fold higher expression in those cells compared to PLum-AD cells. Other biological processes identified including angiogenesis, cell migration, response to oxidative stress, EMT and proteolysis, and are relevant to invasion and metastasis of PC were shown to be altered, which was statistically different with a *p*-value < 0.05 (Supplementary Table S3).

## DISCUSSION AND CONCLUSIONS

In PC research, *in vitro* cell culture models of prostate carcinogenesis are not widely available, where there is a void in cell lines representing primary adenocarcinoma of the prostate as well as the progressive stage of the disease into an androgen independent state. We recently generated a novel murine *in vitro* system, namely PLum cells, recapitulating the disease progression in androgen deprived conditions [17]. In this study, we isolated two novel murine PC cell lines derived from androgen-dependent (PLum-AD) and androgen-independent (PLum-AI) PC and characterized their ability for self-renewal, differentiation, migration and invasion potential and tumorigenicity.

Several possible mechanisms have been postulated to explain how PC cells could become androgen refractory; one of which is the bypass of the AR activation pathway as a result of different factors as *Pten* inactivation, *TP53* mutations, Bcl-2 pathway alterations, neuroendocrine (NE) factors, and alternative growth factor regulation and utilization [16]. In a recent cohort study of 150 CRPC affected individuals recruited in 2015, it is shown that *TP53* and AR alterations were the mostly augmented in CRPC compared to primary PC [21]. It is noteworthy to state that the two novel cell lines described in this study were isolated from *Pten-/−TP53-/−* tumors [17] and therefore serving as a good model to study CRPC. Other studies stated that the combined deletion of *Pten* and *TP53* is characterized by stem cell features and EMT, where increased stem/progenitor activity is apparent by the expanded progenitor self-renewal activity *in vitro* and the histologically diverse tumor formation [18, 22]. Interestingly, our newly derived PLum-AD and PLum-AI cell lines possess this feature of forming spheres *in vitro*. To date, there is limited knowledge concerning the differentiation status and correlative tumorigenic and metastatic properties of PC tumor-initiating cells, whereby the effect of androgen deprivation therapy on the status of these populations of cells remains an essential question [23–25]. However, our model can be the best fit to answer this question by extensively studying these intricate genetic variations and downstream targets in the PLum-AD and PLum-AI cell lines after which new therapies could be derived to overcome castration resistance.

In our study, results of both *in vitro* and *in vivo* experiments done to characterize the newly isolated PLum-AD and PLum-AI cells correlate with the hypothesis that PLum-AD cells might represent the early stage of PC whereas PLum-AI cells might represent the CRPC stage of the disease. The lack of AR in metastases also suggests a relatively immature metastasis-initiating cell. It is important to note that no metastasis was detected following subcutaneous injection of cancer cells in mice. This might be due to the subcutaneous site of injection of tumor cells that we used suggesting that further *in vivo* approaches at different sites of injection are needed to clarify their ability to metastasize as seen in CRPC.

Our results strongly suggest that the newly isolated cell lines mimic the different stages of PC. Therefore, we used them to check the differences in gene expression profile using microarray analysis and to emphasize on the different pathways that are modified in PLum-AI compared to PLum-AD cells in favor of metastasis. Interestingly, the microarray results identified a list of genes that are either upregulated or downregulated in Plum-AI compared to Plum-AD. Among these genes, four were already selected and used to characterize both cell lines (*Vim, Krt18, Krt14 and Cdh1*). The microarray results showed a clear upregulation of vimentin and downregulation of CK14, CK18 and Cdh1 in Plum-AI compared to Plum-AD (Supplementary Table S1) in accordance with our real time PCR results (Figure 1C). Therefore, further analysis were carried forward using systems biology to identify specific pathways, biological processes and signaling molecules that might contribute to PC development and the progression from androgen-dependent to androgen-independent state. One of the listed genes is EPCAM gene, encoding epithelial cell adhesion molecule. It has been reported that EPCAM plays an important role in PC proliferation, invasion, metastasis and chemo-/radio resistance [26]. Interestingly, our analysis revealed that *EPCAM* gene was significantly downregulated in PLum-AI cells (96-fold) compared to PLum-AD cell which might contribute to the aggressive nature of androgen-independent cells. Moreover, the prostate as an organ is known to be exceptionally susceptible to continual oxidative stress, as a consequence of inflammation, hormonal deregulation, diet, and/or some epigenetic modifications [27]. It is noteworthy that several lines of evidence have suggested that one of the major aging-associated influences on prostate carcinogenesis is oxidative stress and its cumulative impact on DNA damage [28–30]. Our analysis showed that the oxidative stress pathway is activated in Plum-AI and several genes related to the response to oxidative stress are upregulated. Moreover, Wnt signaling is shown to have a role in regulating the self-renewal of PC cells with stem cell characteristics irrespective of the androgen receptor activity [31]. It is also one of the key signaling pathways that induce EMT [32]. The association between EMT and cell invasion has also been verified in cancer progression [32], whereby MMPs as MMP-2 and MMP-9 increase the enzymatic degradation of extracellular matrix components inducing cell invasion and tumor spread [33]. In our study, it is shown that *MMP2* gene was significantly upregulated in PLum-AI cells, displaying 33-fold higher expression in those cells compared to PLum-AD cells. Finally, using systems biology analysis, several processes believed to be central to the aggressive nature of PLum-AI cells included EMT, cell migration and invasion mechanisms, along with a marked alteration in the pro-apoptotic genes (Supplementary Table S4).

**Figure 7:**
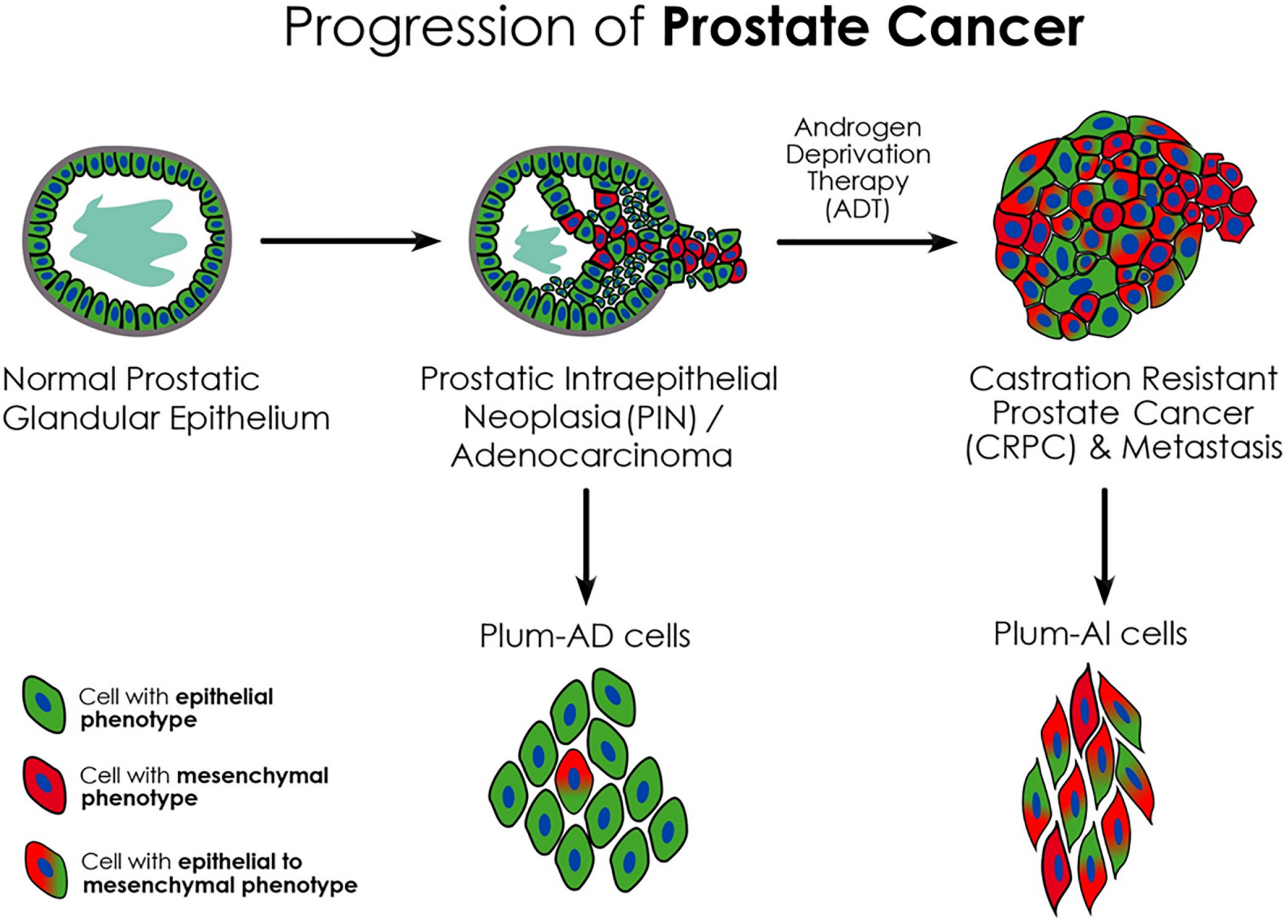
Schematic model showing phenotypic progression of PC from primary adenocarcinoma to CRPC following ADT PLum-AD cells demonstrate an epithelial phenotype and are derived from prostate adenocarcinoma tumors representing the primary stage of PC, whereas PLum-AI cells are derived from androgen-deprived tumors undergoing EMT thus representing the advanced stage of the disease.

Altogether, these data suggest that the newly isolated cell lines represent a new *in vitro* model of androgen-dependent and –independent PC, where they recapitulate the progression of the disease to a more invasive phenotype upon androgen deprivation (Figure 7). These cell lines recapitulate many aspects of human PC, namely the formation of relevant tumor types and the progression from androgen-dependent to androgen-independent PC. Therefore, these novel cell lines will be helpful as *in vitro* model to decipher the molecular mechanisms involved in this progression that would eventually lead to new therapeutic targets.

## MATERIALS AND METHODS

### Ethics statement

All animal experiments were done according to the protocol approved by the Institutional Animal Care and Utilization Committee (IACUCC# 10-07-154) of the American University of Beirut and according to the NIH Guide and the American University of Beirut Guidelines for Use and Care of Animals.

### Animal housing conditions

NOD-SCID mice used for *in vivo* transplantation of PLum-AD or PLum-AI cells were housed in specific pathogen-free animal housing and maintained under identical conditions. Animal experimentation was approved by the Institutional Animal Care and Utilization Committee (IACUCC# 10-07-154) of the American University of Beirut and conducted using the standards for animal care according to the NIH Guide and the American University of Beirut Guidelines for the Care and Use of Laboratory Animals.

### Cell culture

### Reagents

PrEGM with supplements was purchased from Lonza (Lonza, MD). FBS, antibiotic reagents and trypsin were purchased from Sigma-Aldrich (Saint Louis, MO). Dispase and Collagenase Type II were purchased from Invitrogen (Invitrogen, CA). BD Matrigel^™^ was purchased from BD Biosciences (BD Biosciences, CA).

### 2D culture

Orthotopic tumors under androgen-dependent or androgen-independent conditions were generated previously [17]. Tumors were then taken, minced into small pieces and incubated with Collagenase II/PrEGM for 1 hr at 37°C on a rotating wheel. Cells were then carefully passed through 19-, 23-, 25-, 27-, and 30.5-gauge needles. After washing, the suspension was then passed through 40-mm cell strainer and centrifuged for 5 minutes at 200 g. Finally, the pellet was resuspended in PrEGM. PLum-AD cells are generated from androgen-dependent orthotopic tumors, and PLum-AI cells are generated from androgen-independent tumors. PLum-AD and PLum-AI single cells were suspended in T75 cm^2^ flasks with PrEGM, a serum-free prostate epithelial cell basal media, supplemented with bovine pituitary extract, insulin, hydrocortisone, gentamicin, amphotericin B, retinoic acid, transferrin, T3 (3,3ʹ,5-triiodo-L-thyronine), epinephrine, recombinant human epidermal growth factor, and penicillin/streptomycin as directed by the manufacturer. The cells were incubated at 37°C in a humidified 5% CO_2_ incubator. Cell media were changed every 2–3 days. PLum-AD cells were cultured in serum free media while PLum-AI cells, which grew better in 5% FBS-containing media, were all time of culture in this media. Cells were propagated using regular trypsinization techniques after becoming 50–70% confluent. Occasionally, bright field phase contrast images, using an inverted light microscope, were taken to evaluate any morphological changes.

### 3D cultures and sphere-formation assay

Single cells were suspended, in duplicates, in 100 μl volume of 1:1 Matrigel^™^ and serum free PrEGM solution at a density of 2,000 cells/well. This suspension was plated smoothly around the rim of each well of a 12-well plate and left for 1 hour at 37°C in a 5% CO_2_ humidified incubator to solidify. The serum free PrEGM cell medium was gently added to the center of each well, and was replenished every 2–3 days. Spheres were harvested at 12–15 days after plating. The sphere-forming unit (SFU) was expressed as a percentage of the counted number of formed spheres by the total number of plated cells.

### Sphere propagation and self-renewal ability assessment

The cell media was gently aspirated from the center of the well, and the Matrigel^™^ at the rim of each well was digested by adding 500 μl of serum-free PrEGM containing dispase (1 mg/ml), followed by incubation at 37°C in a humidified 5% CO_2_ incubator for an hour. The resulting sphere-containing solution was collected and centrifuged at 200 g for 6 minutes. The resulting pellet was suspended in 500 μl of 0.05% trypsin-EDTA solution. This solution was then immediately and carefully passed through a series of 21, 25, 27, and 31 gauge needles. PrEGM medium containing 2% fetal bovine serum (FBS) was added to inactivate trypsin, and cells were then subjected to another round of centrifugation (200 g for 6 minutes). The resulting pellet was then washed and resuspended in serum-free PrEGM. The trypan blue exclusion method was used to quantify viable cells. Cells were then suspended in Matrigel^™^ and plated as described previously at a density of 2,000 cells per well. The spheres were propagated for a minimum of five generations and the SFU was calculated for each generation as previously described.

### Immunohistochemistry, immunofluorescence and confocal microscopy

#### Antibodies and reagents

Antibodies from the indicated manufacturers used in this study were as follows: mouse monoclonal anti-CK8 (1/200 dilution), rabbit polyclonal anti-CK14 (1/200 dilution) (Covance, CA); rabbit polyclonal anti-AR (1/50 dilution) and rabbit polyclonal anti-Vimentin (1/50 dilution) (Santa Cruz Biotechnology, CA); Rabbit polyclonal anti-beta III Tubulin antibody (1/50 dilution) (Abcam Inc., MA); rhodamine phalloidin (1/100 dilution); Alexa 488 goat anti-mouse, goat anti-rabbit, Alexa 568 goat anti-mouse, goat anti-rabbit (Invitrogen, CA); Biotinylated goat anti-rabbit IgG (Vector Laboratories, Burlingame, CA). All secondary Alexa Fluor antibodies were used at 1/100 dilution, and the secondary biotinylated antibody was used at 1/200 dilution. Fluoro-gel II with Dapi was purchased from EMS (Electron Microscopy Sciences, PA).

### Histology and immunohistochemistry

Subcutaneous tumor tissues were harvested and fixed in 4% PFA overnight, rinsed well in PBS and transferred to 70% ethanol before standard processing, to obtain paraffin-embedded sections. Certain sections were stained with hematoxylin and eosin (H & E), and examined by a light microscope. The remaining unstained tissue sections were deparaffinized, and antigen retrieval was performed in a citrate buffer in a steamer at 100°C for 60 min followed by 30 min incubation at room temperature. Slides were treated with Novolink^™^ peroxidase block (Leica biosystems, UK) for 5 min, and then blocking was performed with protein block (3% BSA, 0.1% Triton X-100, and 10% normal goat serum in PBS) for 5 min at room temperature. Primary antibody incubation in primary antibody buffer (3% BSA, 0.1% Triton X-100, and 2% normal goat serum in PBS) was performed overnight at 4°C, followed by secondary biotinylated antibody incubation at room temperature for 30 min. The ABC peroxidase kit (Vector Laboratories, Burlingame, CA) was used followed by DAB (Dako, Carpinteria, CA) for chromogen visualization. All slides were counterstained with hematoxylin.

### Immunofluorescent staining procedure for tissues

Immunofluorescence was performed on tumor tissue sections using the same protocol as used for IHC with the following exceptions: no peroxidase block was used here; protein blocking was performed using the same blocking buffer for an hour at room temperature; the secondary antibodies were Alexa Fluor 488 conjugated goat anti-mouse and goat anti-rabbit IgG and Alexa Fluor 568 conjugated goat anti-rabbit and goat anti-mouse IgG in secondary antibody buffer (2% normal goat serum and 0.1% Triton X-100 in PBS). Slides were mounted with the anti-fade Fluoro-gel II with Dapi.

### Immunofluorescent staining procedure for monolayer cells

Prostate epithelial lineage markers expressed by the PLum-AD and PLum-AI cells grown in 2D monolayer cultures were characterized using indirect immunofluorescence analysis. Adherent cells were fixed in 4% paraformaldehyde (PFA) in phosphate buffered saline (PBS) for 10 minutes followed by permeabilization with 0.5% Triton X-100 in PBS for 4 minutes. The cells were then incubated in blocking buffer (0.1% BSA, 0.2% Triton X-100, 0.05% Tween-20, and 10% normal goat serum in PBS) for half an hour to block non-specific sites, and afterwards, the cells were incubated overnight at 4°C in 2% BSA in PBS with the specified primary antibodies. The cells were then washed three times with PBS containing 0.1% Tween-20 and incubated with Alexa-488 and/or 568 conjugated IgG secondary antibodies in 2% BSA in PBS for 30 minutes at room temperature. The cells were later washed and mounted using anti-fade reagent (Fluoro-gel II with Dapi).

### Immunofluorescent staining procedure for protospheres

Cells were grown in 12-well plates in Matrigel^™^ containing media as described above. Spheres were fixed in-situ in 4% PFA at room temperature for 20 min and then collected by pipetting up and down several times. The suspension was centrifuged at 200 g for 2 min and the spheres pellet was permeabilized with 0.5% Triton X-100 for 30 min at room temperature. After centrifugation and carefully aspirating the permeabilization solution, spheres were blocked using the sphere blocking buffer (same as the one used in IF for monolayer cells) for 2 hrs at room temperature. Spheres were then incubated overnight with primary antibodies at 4°C. After washing with PBS containing 0.1% Tween-20, spheres were incubated with Alexa-488 and/or 568 conjugated IgG secondary antibodies for 2 hrs at room temperature. After washing and centrifugation, 10 ml of the anti-fade reagent Fluoro-gel II with Dapi was added directly and the pellet was collected and mounted on glass slides and covered by thin glass coverslip.

### Microscope specifications

Confocal microscopic analyses were performed using Carl Zeiss LSM 710 laser scanning confocal microscope and images were acquired and analyzed using the Carl Zeiss ZEN 2012 image software.

### *In vivo* transplantation

A total of 2 × 10^6^ PLum-AD or PLum-AI cells in 50 μl were mixed 1:1 with growth factor reduced MatrigelTM (BD Bioscience) immediately prior to injection. PLum-AD or PLum-AI cells were injected subcutaneously into the flanks of 6–8 week old male NOD-SCID mice. The mice were grouped into two groups of 15 each. Measurements of tumor volume and survival experiments were carried. Mice were sacrificed at twelve weeks following the first tumor palpation, unless they showed earlier signs of morbidity. After detecting any palpable tumor following injections, tumor size measurements were started twice per week by direct physical measurements to determine tumor size and expansion. Measurements were carried out until the termination of the experiment. All mice were housed in specific pathogen-free animal housing. Animals were sacrificed, after deep anesthesia with isoflurane, through cervical dislocation.

### RNA extraction and Quantitative real time PCR (qRT-PCR)

RNeasy Micro Kit (Qiagen) was used to extract total RNA from 1 × 10^6^ PLum-AD and PLum-AI cells. Super Script III First Strand Synthesis System for RT-PCR (Invitrogen) was used to generate cDNA from the extracted total RNA. Platinum Taq Polymerase (Invitrogen) was used to perform PCR. The amplification step in quantitative real time PCR was carried out using SYBR green PCR master mix (Applied Biosystems, Bedford, MA). All the reactions were done in duplicates using specific primers. The values were normalized to the house keeping gene GAPDH. The primers used were:

*Gapdh*-F: 5ʹCAGAACATCATCCCTGCATC3ʹ;

R: 5ʹCTGCTTCACCACCTTCTTGA3ʹ.

*E-cadherin-*F: 5ʹGACAACGCTCCTGTCTTCAA3ʹ;

R: 5ʹACGGTGTACACAGCTTTCCA3ʹ.

*CK18-*F: 5ʹCTGGTCTCAGCAGATTGAGG3ʹ;

R: 5ʹCTCCGTGAGTGTGGTCTCAG3ʹ.

*CK14-*F: 5ʹGATGACTTCCGGACCAAGTT3ʹ;

R: 5ʹTGAGGCTCTCAATCTGCATC3ʹ.

*Vimentin-*F: 5ʹAAACGAGTACCGGAGACAGG3ʹ;

R: 5ʹTCTCTTCCATCTCACGCATC3ʹ.

*AR-*F: 5ʹGACTCTGGGAGCTCGTAAGC3ʹ;

R: 5ʹACTCCTGGCTCAATGGCTTC3ʹ.

### Western blot analysis

2× Laemmli sample buffer (65.8 mM Tris-HCl, pH 6.8, 2.1% SDS, 26.3% (w/v) glycerol, 0.01% bromophenol blue; BIO-RAD) was used to extract protein samples which were then loaded onto a 10% sodium dodecyl sulfate-polyacrylamide gel. Electrophoresis was done and proteins were then transferred onto nitrocellulose membranes. The membranes were blocked using 5% skimmed milk in PBS containing 0.05% Tween-20 for 30 minutes at room temperature, after which they were incubated overnight at 4°C with the specific primary antibodies. The GAPDH antibody (Novus Biologicals, USA) was used to ensure for equal protein loading. After incubation, the membranes were washed and incubated with HRP-conjugated secondary antibodies. Chemiluminescence was used to visualize the protein bands that were detected using x-ray films. All images were analyzed and quantified using NIH image J software and the signal intensity ratio of proteins/GAPDH are plotted on the graph.

### Transwell migration assay

PLum-AI cell were starved overnight before the experiment. Cells were seeded at a density of 2 × 10^5^ cells/well onto 8 μm pore size inserts (Falcon) which were placed in 24-well plates containing PrEGM in the presence or absence of 5% FBS in the lower chamber used as chemoattractant. The cells were left for 24 hours at 37°C in a humidified 5% CO_2_ incubator. After one day, the inserts were removed and cells that failed to migrate through the inserts were smoothly scraped off using a cotton swab. Cells on the inserts were fixed in Paraformaldehyde and stained with H & E. The membrane of the inserts was then cut and mounted on a microscopic slide for examination using a light microscope. Migration quantification was performed by counting the number of cells that migrated through the inserts. To do that, five separate randomly selected fields were photographed and counted under 20× magnification. Cell migration was expressed as a percentage of migrated cells in the absence of any stimulant.

### Transwell invasion assay

Plum-AI cell were starved overnight before the experiment. Cells were seeded at a density of 2 × 10^5^ cells/well onto 8 μm pore size inserts (Falcon) coated with growth factor reduced MatrigelTM. The inserts were then placed in 24-well plates containing PrEGM in the presence or absence of 10% FBS. The cells were left for 24 hrs at 37°C in a humidified 5% CO_2_ incubator. After one day, the inserts were removed and cells that failed to migrate through the inserts were smoothly scraped off using a cotton swab. Cells on the inserts were fixed in Paraformaldehyde and stained with H & E. The membrane of the inserts was then cut and mounted on a microscopic slide for examination using a light microscope. Invasion quantification was performed by counting the number of cells that invaded through the Matirgel^™^ coated inserts. To do that, five separate randomly selected fields were photographed and counted under 20× magnification. Cell invasion was expressed as a percentage of invaded cells in the absence of any stimulant.

### RNA extraction and microarray hybridization

PLum-AD and PLum-AI cells were grown up to 50–70% in confluency, and then RNeasy Micro Kit (Qiagen) was used to extract total RNA. The RNA samples were hybridized to the mouse Affymetrix Gene Chip 2.0 ST (Affymetrix, Santa Clara, CA). A total of 3 microarray analyses representing 3 biological replicates were conducted for each cell line.

### Microarray data analysis

The microarray data analysis was performed within the R statistical environment. Raw data was normalized using the Robust Multiarray Averaging (RMA) method [34]. Differential expression analyses of the PLum-AD vs. PLum-AI cells were performed using limma [35]. The *p*-values were adjusted for multiple testing using Benjamini and Hochberg (BH) method [36]. A double threshold of adjusted *p*-value of 0.05 and logged fold change of at least 2 was used. PathwayStudio software (v 9.0; Ariadne Genomics, Rockville, MD, USA) was used to assess the altered biological pathways and processes relevant to deprivation of androgen and its contribution to the development of aggressive PC. This bioinformatics platform is used to interpret biological meaning from differential gene expression, build and analyze pathways, and identify altered cellular processes and molecular functions involved.

### Data analysis

Statistical calculations were done with GraphPad Prism 5 analysis software. The significance of the data was analyzed using the student's *t*-test or one way ANOVA followed by Tukey's Multiple comparison test, and differences between two means with a *p* value < 0.05 were considered significant.

## SUPPLEMENTARY MATERIALS TABLES


