# Supplementary material for: Primary versus castration-resistant prostate cancer: modeling through novel murine prostate cancer cell lines
Source: Oncotarget. 2016 Mar 28;7(20):28961–75. doi: 10.18632/oncotarget.8436 (PMC5045370; doi:10.18632/oncotarget.8436)
Supplement: Supplementary file 1 [file oncotarget-07-28961-s001.pdf]

Primary versus castration-resistant prostate cancer: modeling through novel murine prostate cancer cell lines

Supplementary Materials

Supplementary Table S1: Differentially expressed (DE) genes

Gene Row Names	Choromosome Number	Gene Symbol	Gene Description	logFC	Fold Change	*t*-value	Adjusted *P* value	OIAD.3	OIAD.1	OIAD.2	OIAI.3	OIAI.1	OIAI.2
17383886	2	1110008P14Rik	RIKEN cDNA 1110008P14 gene	2.327299300	5.018649872	7.904444720	0.000837940	–1.130256388	–0.884145546	–0.684960319	1.066069220	0.842238180	0.791054853
17358094	19	1500015L24Rik	RIKEN cDNA 1500015L24 gene	–2.641467850	0.160265095	–8.922081004	0.000398122	0.717311575	0.943681310	1.045303159	–1.063158390	–0.716653238	–0.926484414
17219902	1	1700016C15Rik	RIKEN cDNA 1700016C15 gene	–2.212365353	0.215780238	–7.394346208	0.001187575	0.565619692	1.116812204	1.005837307	–0.986116450	–0.849011934	–0.853140820
17419613	4	1810019J16Rik	RIKEN cDNA 1810019J16 gene	–2.657722778	0.158469513	–8.760932175	0.000451699	0.916727854	0.815530141	0.966823097	–1.194831080	–0.771725613	–0.732524399
17268332	11	2010300F17Rik	RIKEN cDNA 2010300F17 gene	–4.696390111	0.038569651	–14.961702079	2.22E–05	0.841383477	0.997998196	0.882680496	–1.045829260	–0.742431135	–0.933801774
17305163	14	2610528A11Rik	RIKEN cDNA 2610528A11 gene	4.921493588	30.305202721	10.382298245	0.000172657	–0.932284408	–1.218133811	–0.509983869	0.846745060	1.019209900	0.794447128
17517903	9	4930461G14Rik	RIKEN cDNA 4930461G14 gene	2.038999174	4.109603405	5.583190597	0.005481326	–0.753444336	–1.018762050	–0.789568157	0.300929661	0.880663365	1.380181517
17217048	1	5430435G22Rik	RIKEN cDNA 5430435G22 gene	2.553130806	5.869065487	6.980406265	0.001605940	–1.314597231	–0.812935477	–0.493572338	0.693742254	1.083502919	0.843859873
17271307	11	Abca8b	ATP-binding cassette, sub-family A (ABC1), member 8b	2.482434471	5.588396837	9.237914265	0.000323310	–0.827651549	–0.888889011	–1.016815700	0.929932276	0.89926281	0.904161176
17267846	11	Abcc3	ATP-binding cassette, sub-family C (CFTR/MRP), member 3	3.548976979	11.704383001	9.987288839	0.00021174	–1.01602649	–1.093057433	–0.573909942	0.732049312	1.023192697	0.927751857
17326318	16	Abi3bp	ABI gene family, member 3 (NESH) binding protein	2.626710191	6.176160272	7.732791844	0.000937748	–1.164152790	–0.895502226	–0.598112412	1.179558102	0.714847055	0.763362273
17364098	19	Acta2	actin, alpha 2, smooth muscle, aorta	2.078457471	4.223553924	6.209633463	0.003199180	–0.971970686	–0.645180842	–1.004266680	0.413585797	1.209149074	0.998683337
17497246	7	Adam12	a disintegrin and metallopeptidase domain 12 (meltrin alpha)	–2.836737910	0.139977038	–9.891267083	0.000222185	0.871363371	0.851629266	0.995878039	–1.077199475	–0.909659936	–0.732011265
17279349	12	Adssl1	adenylosuccinate synthetase like 1	2.615602641	6.128791565	6.867275278	0.001806848	–1.386160980	–0.626308426	–0.595462794	1.043136499	0.880614460	0.684181241
17247039	11	Aebp1	AE binding protein 1	6.078735110	67.589868865	20.650313439	1.13E–05	–0.847928007	–1.008628977	–0.876208104	0.925152601	0.847653537	0.959958950
17254194	11	AI662270	expressed sequence AI662270	–3.091961573	0.117280773	–10.605129335	0.000156631	0.880343735	1.047568986	0.790451892	–0.746439609	–0.959849921	–1.012075083
17284919	13	Akr1c14	aldo-keto reductase family 1, member C14	2.640421359	6.235137428	7.354432753	0.001219757	–1.14838308	–0.868048686	–0.619790636	0.794220865	1.249859199	0.592142338

17290263	13	Akr1c18	aldo-keto reductase family 1, member C18	6.393182374	84.050376307	14.526272481	2.78E–05	–0.990437751	–1.027710138	–0.682537498	0.759665410	0.836128654	1.104891324
17250567	11	Aldh3a1	aldehyde dehydrogenase family 3, subfamily A1	2.389494695	5.239738071	4.533977256	0.014145415	–1.274897428	–0.974560334	–0.116623432	0.359468924	0.636697781	1.369914489
17361041	19	Aldh3b1	aldehyde dehydrogenase 3 family, member B1	2.438179242	5.419573200	7.273922317	0.001287311	–1.262163701	–0.869558439	–0.519598521	0.735411338	0.909766547	1.006142775
17431720	4	Alpl	alkaline phosphatase, liver/bone/kidney	2.016366870	4.045636978	6.535308243	0.002405423	–0.894350392	–0.917076783	–0.850406502	0.495161320	0.935862372	1.230809986
17545159	X	Amot	angiomotin	–2.170506248	0.222132709	–5.995285045	0.00378791	1.149365842	0.357824275	1.078843119	–0.808361093	–0.611788519	–1.165883624
17316780	15	Angpt1	angiopoietin 1	2.493427025	5.631140024	7.776241054	0.000906140	–1.219468433	–0.891036514	–0.562495627	0.789979111	0.994413624	0.888607839
17310673	15	Ank	progressive ankylosis	2.133022752	4.386355528	7.861799196	0.000854367	–0.976190712	–0.982284798	–0.769136375	0.908892842	0.836414930	0.982304114
17364090	19	Ankrd22	ankyrin repeat domain 22	–4.196271387	0.054550212	–13.497335908	4.31E–05	0.730664069	0.916429986	1.072163234	–0.841422187	–0.991966895	–0.885868207
17498467	7	Ano1	anoctamin 1, calcium activated chloride channel	–3.125317641	0.114600271	–9.395568190	0.000294106	0.942189951	0.724632882	1.019762574	–1.178953553	–0.855946217	–0.651685637
17468573	6	Antxr1	anthrax toxin receptor 1	4.706055321	26.101400636	15.449234010	1.93E–05	–1.022198361	–0.868910446	–0.834274826	1.041189681	0.812431507	0.871762443
17524703	9	Ap1m2	adaptor protein complex AP–1, mu 2 subunit	–3.517932540	0.087296490	–10.784609040	0.000144261	0.820022847	0.727034934	1.154434891	–0.793399152	–1.044518202	–0.863575318
17351196	18	Apcdd1	adenomatosis polyposis coli down-regulated 1	2.517598474	5.726281025	7.730990891	0.000937748	–0.533765923	–1.047647417	–1.086755073	1.034880739	0.942540196	0.690747477
17276776	12	Arg2	arginase type II	2.876009436	7.341167071	9.641700769	0.000253442	–1.121685952	–0.735145055	–0.852464221	0.999684680	0.938272496	0.771338052
17506147	8	Atp2c2	ATPase, Ca++ transporting, type 2C, member 2	–2.055624216	0.240544511	–5.042614585	0.008872274	0.297926998	0.904567399	1.284190906	–1.328209308	–0.513367828	–0.645108167
17355026	18	Atp8b1	ATPase, class I, type 8B, member 1	–3.345072584	0.098408546	–11.373104461	0.000110941	0.801148967	0.837692916	1.080761586	–0.994542320	–0.787188357	–0.937872791
17470394	6	B4galnt3	beta–1,4-N-acetyl-galactosaminyl transferase 3	–3.034704921	0.122028927	–10.017265665	0.000207942	0.918210479	0.971880249	0.818384653	–1.145360346	–0.714214255	–0.848900779
17539682	X	BC022960	cDNA sequence BC022960	2.337749938	5.055136112	6.173557868	0.003276747	–1.422035656	–0.479271238	–0.679833915	0.956185255	0.972804010	0.652151544
17475580	7	Blvrb	biliverdin reductase B (flavin reductase (NADPH))	2.200857949	4.597526686	6.99440969	0.001594693	–1.141914593	–0.567545581	–0.954960706	0.717596735	1.134604388	0.812219757

17414572	4	Bspry	B-box and SPRY domain containing	–2.167785772	0.222551978	–8.098061241	0.000730768	0.906345213	0.980598803	0.846272871	–0.832674501	–0.923728147	–0.976814239
17319022	15	C1qtnf6	C1q and tumor necrosis factor related protein 6	3.073370120	8.417373415	11.255984478	0.000117069	–0.818601987	–0.930348272	–0.983083981	0.966782369	0.933437033	0.831814838
17267885	11	Cacna1g	calcium channel, voltage-dependent, T type, alpha 1G subunit	3.327650179	10.039741226	9.099749854	0.000350540	–0.556616985	–0.940294736	–1.170724303	1.047491258	0.923552515	0.696592252
17473155	7	Cacng7	calcium channel, voltage-dependent, gamma subunit 7	2.297954866	4.917601621	8.240839717	0.000647518	–1.008051531	–0.744241348	–0.966971955	1.035451685	0.791267588	0.892545560
17498673	8	Camsap3	calmodulin regulated spectrin-associated protein family, member 3	–2.186559288	0.219674713	–7.651879532	0.000992518	0.853085068	0.795213592	1.058859190	–0.926078396	–0.696948379	–1.084131075
17310250	15	Capsl	calcyphosine-like	–2.126763090	0.228971019	–7.326306769	0.001230195	0.635525483	1.036149100	1.026640925	–0.739581992	–0.939299327	–1.019434188
17545936	X	Car5b	carbonic anhydrase 5b, mitochondrial	2.016520793	4.046068636	4.918380830	0.009798782	–1.592753009	–0.326180694	–0.554799775	0.891021012	0.826946930	0.755765537
17514424	9	Casp1	caspase 1	6.316517526	79.700536250	20.187648010	1.13E–05	–0.945085899	–0.818398518	–0.966141186	0.806004698	0.918190205	1.005430700
17514435	9	Casp4	caspase 4, apoptosis-related cysteine peptidase	2.032786496	4.091944267	4.124358252	0.021065449	–1.732935126	–0.243398652	–0.345856595	0.774604203	0.672419977	0.875166193
17327264	16	Cbr3	carbonyl reductase 3	2.987761400	7.932421837	7.757605802	0.000919003	–1.096544202	–0.712079122	–0.824003363	0.443114478	1.190203201	0.999309008
17366536	2	Ccdc3	coiled-coil domain containing 3	–2.087608094	0.235270429	–5.992359653	0.003793797	0.372871998	1.228560994	0.997272158	–0.685901692	–0.787311608	–1.125491849
17351634	18	Ccdc68	coiled-coil domain containing 68	–2.398594002	0.189649306	–7.354207961	0.001219757	0.596893627	0.871945385	1.191800071	–1.149289167	–0.749081046	–0.762268871
17325904	16	Ccdc80	coiled-coil domain containing 80	3.770774907	13.649487773	12.618868700	6.25E–05	–1.052953880	–0.732986004	–0.935223447	0.844725817	0.989726877	0.886710636
17254041	11	Ccl2	chemokine (C-C motif) ligand 2	3.157552587	8.923146873	9.489827785	0.000271849	–0.853591991	–0.961135707	–0.872810522	0.555977442	1.016211909	1.115348869
17266946	11	Ccl5	chemokine (C-C motif) ligand 5	2.947119211	7.712075688	5.555801922	0.005632645	–1.556753282	–0.601330122	–0.314053282	0.700528332	1.053514964	0.718093390
17254047	11	Ccl7	chemokine (C-C motif) ligand 7	3.124316078	8.719927126	10.615719376	0.000156631	–0.964051282	–1.065539931	–0.687122131	0.861776845	0.931879584	0.923056916
17531705	9	Ccrl2	chemokine (C-C motif) receptor-like 2	2.399138839	5.274882067	4.739406596	0.011779296	–1.658326571	–0.423483498	–0.318136629	0.624969006	0.845389933	0.929587758
17264835	11	Cd68	CD68 antigen	2.519993387	5.735794700	4.058456904	0.022681731	–1.388561776	–0.426699402	–0.448417966	1.358906386	0.917580802	–0.012808043
17505148	8	Cdh1	cadherin 1	–4.831955407	0.035110457	–17.612115546	1.13E–05	0.890376909	0.883730637	0.961447081	–0.906091792	–0.975555604	–0.853907232
17310530	15	Cdh10	cadherin 10	–3.260109075	0.104378098	–11.283700463	0.000116004	0.917707696	0.756941988	1.047529127	–1.009447347	–0.826152655	–0.886578809
17411848	4	Cdh17	cadherin 17	2.139492682	4.406070813	5.448283415	0.006187456	–0.761309488	–0.804598458	–0.963625032	0.338931580	0.688967013	1.501634384

17305143	14	Cdhr1	cadherin-related family member 1	3.263574195	9.603592545	9.695192117	0.000245044	–0.92410581	–1.173540605	–0.590118738	0.805876092	0.926779734	0.955109327
17462373	6	Cecr2	cat eye syndrome chromosome region, candidate 2	2.307791891	4.951246895	6.964204731	0.001631058	–0.917101145	–0.807654593	–0.922442170	1.321252147	0.787364744	0.538581018
17479027	7	Cers3	ceramide synthase 3	–2.485315208	0.178585245	–7.407826314	0.001181366	0.744918621	0.915773809	0.993374784	–1.302197218	–0.747234643	–0.604635354
17504572	8	Ces2g	carboxylesterase 2G	2.272052289	4.830097423	6.755504373	0.001989968	–1.083224653	–1.085565757	–0.468334530	0.634203754	0.899546254	1.103374933
17344064	17	Cfb	complement factor B	2.212842985	4.635879236	7.849811348	0.000861584	–1.049202338	–0.715423558	–0.948795827	0.798277024	0.868145701	1.046998998
17227696	1	Cfh	complement component factor h	2.883299494	7.378356485	7.008632206	0.001573813	–0.631341298	–1.250193644	–0.715919966	1.29335131	0.689347321	0.614756277
17407594	3	Cgn	cingulin	–2.715020068	0.152299165	–9.225417925	0.000325097	0.992322348	0.872277818	0.845137097	–0.843462363	–0.724269917	–1.142004983
17217399	1	Chi3l1	chitinase 3-like 1	2.799848768	6.963674493	9.240998635	0.000323310	–0.878706268	–1.032997303	–0.791573233	0.860709542	1.140207943	0.702359320
17264946	11	Chrnb1	cholinergic receptor, nicotinic, beta polypeptide 1 (muscle)	–2.667378417	0.157412453	–7.887954178	0.000841782	1.070498020	0.517192632	1.074121283	–1.091917446	–0.720755424	–0.849139065
17453496	5	Cldn4	claudin 4	–5.768219620	0.018348175	–18.246568899	1.13E–05	0.829121269	0.971832211	0.926121604	–0.893608687	–0.792108058	–1.041358339
17334097	17	Cldn6	claudin 6	–5.426760389	0.023247826	–12.937919712	5.44E–05	0.935656681	0.851843989	0.906187590	–1.096201124	–0.575032852	–1.022454284
17251829	11	Cldn7	claudin 7	–3.809984686	0.071298489	–13.116427791	5.21E–05	0.949652414	0.804329327	0.971789361	–0.977974443	–0.766638201	–0.981158459
17302869	14	Clybl	citrate lyase beta like	2.048298151	4.136177649	4.858649155	0.010431024	–1.560301422	–0.472038619	–0.426940187	0.834745079	1.080599942	0.543935207
17255260	11	Col1a1	collagen, type I, alpha 1	4.214445908	18.564131383	12.608481036	6.25E–05	–0.748694597	–0.930424497	–1.029761996	1.060662980	0.946664068	0.701554041
17225413	1	Col6a3	collagen, type VI, alpha 3	3.478642875	11.147458123	11.101200366	0.000126154	–0.781555481	–1.035832502	–0.891624760	1.099681804	0.896736742	0.712594197
17398895	3	Crabp2	cellular retinoic acid binding protein II	2.999022168	7.994579585	10.583053468	0.000156631	–1.062844495	–0.887301157	–0.773495634	0.903655908	0.987437363	0.832548015
17219005	1	Creg1	cellular repressor of E1A-stimulated genes 1	2.854402583	7.232039630	10.262444256	0.000181589	–0.939467429	–1.009790666	–0.777378439	0.808134221	0.959110982	0.959391332
17351053	18	Csf1r	colony stimulating factor 1 receptor	–2.286197422	0.205015171	–6.837133228	0.001854555	0.630085656	0.657837015	1.353241305	–0.958094083	–0.793191923	–0.889877969
17232235	10	Ctgf	connective tissue growth factor	–3.699451888	0.076975765	–10.916556469	0.000135248	0.888852820	0.903760518	0.904817869	–0.974907544	–0.596239558	–1.126284105
17438113	5	Cwh43	cell wall biogenesis 43 C-terminal homolog (S. cerevisiae)	–3.073221889	0.118814113	–9.701653465	0.000245044	0.610266716	0.958894796	1.128912322	–0.855651125	–0.875098922	–0.967323786
17438975	5	Cxcl3	chemokine (C-X-C motif) ligand 3	–2.655507117	0.158713074	–6.563673190	0.002359580	0.934819814	0.824308246	0.823702995	–1.458672584	–0.496698172	–0.627460300
17427389	4	Cyp2j6	cytochrome P450, family 2, subfamily j, polypeptide 6	2.418220172	5.345111977	7.405526592	0.001181366	–0.803652346	–1.134864720	–0.722819994	0.893626520	0.581541197	1.186169343

17239546	10	D10Bwg1379e	DNA segment, Chr 10, Brigham & Women's Genetics 1379 expressed	–2.011430009	0.248027156	–6.711934017	0.002066977	1.026685706	0.628578430	1.021939815	–0.622462002	–0.964111779	–1.090630170
17538079	X	D330045A20Rik	RIKEN cDNA D330045A20 gene	3.153696104	8.899326170	10.460308568	0.000169189	–1.110774256	–0.712351271	–0.888642729	0.797078568	0.938666304	0.976023383
17308794	5	D5Ertd579e	DNA segment, Chr 5, ERATO Doi 579, expressed	2.250347174	4.757973294	6.086939526	0.003503610	–0.987245798	–0.571283965	–1.025365857	1.050364404	1.195997974	0.337533243
17287984	13	Dapk1	death associated protein kinase 1	3.056968399	8.322219840	10.098575063	0.000203358	–0.854033540	–1.052563515	–0.802496427	0.964478676	1.054895351	0.689719455
17318502	15	Dgat1	diacylglycerol O-acyltransferase 1	2.187443447	4.554975983	7.686423689	0.000974335	–1.139316702	–0.820925251	–0.748660666	0.954006427	0.783672288	0.971223905
17532755	X	Dgkk	diacylglycerol kinase kappa	–3.658318710	0.079202034	–13.018660506	5.27E–05	0.991593743	0.821515016	0.916679958	–0.893310130	–0.825441150	–1.011037438
17421312	4	Dhrs3	dehydrogenase/ reductase (SDR family) member 3	2.057784148	4.163463418	7.344940039	0.001220649	–1.061999906	–0.987334363	–0.663060147	0.963469043	0.878688724	0.870236649
17269595	11	Dhx58	DEXH (Asp-Glu-X-His) box polypeptide 58	2.007820989	4.021743269	4.648175184	0.012752221	–1.373688040	–0.522198597	–0.533922782	1.074128811	0.18665368	1.169026929
17251900	11	Dlg4	discs, large homolog 4 (Drosophila)	2.363589677	5.146493046	6.487198661	0.002525422	–0.847189393	–0.652227968	–1.105541592	1.239808442	0.970415198	0.394735313
17308413	14	Dmtn	dematin actin binding protein	–2.147876471	0.225644502	–7.162579537	0.001372392	0.621936535	0.866285182	1.196009670	–0.850996347	–1.034379274	–0.798855766
17303386	14	Dnase1l3	deoxyribonuclease 1-like 3	–2.437434606	0.184611636	–7.215983604	0.001329358	0.718318827	0.838419675	1.091142813	–1.266868390	–0.564165636	–0.816847288
17383858	2	Dnm1	dynamin 1	2.933376889	7.638963453	10.247113915	0.000182252	–1.099460988	–0.780209219	–0.840907171	0.980433852	0.874192367	0.865951158
17435834	5	Dpysl5	dihydropyrimidinase-like 5	–2.837843412	0.139869818	–9.708045285	0.000245044	0.989925201	0.848253428	0.875757518	–0.921788909	–0.698624467	–1.093522771
17243868	10	Dram1	DNA-damage regulated autophagy modulator 1	3.456635460	10.978700999	11.806145452	8.87E–05	–1.024396920	–0.971900863	–0.725324475	0.977373988	0.913736285	0.830511986
17348812	18	Dsg2	desmoglein 2	–2.025894686	0.245552823	–5.818807745	0.004370191	0.773161205	0.605332223	1.212641240	–1.322704775	–0.709350683	–0.559079210
17535600	X	Dusp9	dual specificity phosphatase 9	–2.16966571	0.222262165	–6.370741437	0.002772102	1.347992601	0.797972122	0.475092394	–0.942462265	–0.947390440	–0.731204413
17355298	18	Dynap	dynactin associated protein	–4.815723200	0.035507727	–15.732089654	1.87E–05	0.932772688	0.937757452	0.854949917	–1.077990562	–0.809043684	–0.838445811
17407850	3	Ecm1	extracellular matrix protein 1	2.122250005	4.353724158	6.709929264	0.002066977	–1.250032214	–0.81422511	–0.592151071	0.699081913	0.879832460	1.077494021
17543396	X	Eda2r	ectodysplasin A2 receptor	2.812345249	7.024255143	8.088911758	0.000731926	–0.942165070	–0.932960375	–0.784406855	1.112151896	1.083965009	0.463415395

17281084	12	Egln3	EGL nine homolog 3 (C. elegans)	4.894357268	29.740505575	13.239862826	4.90E–05	–1.036190085	–1.052535378	–0.614879374	0.856356392	0.944755532	0.902492913
17491026	7	Emp3	epithelial membrane protein 3	2.754374900	6.747602091	8.144078706	0.000705684	–0.707540916	–1.013597753	–0.945211239	0.642629568	0.769077969	1.254642371
17425533	4	Epb4.1l4b	erythrocyte protein band 4.1-like 4b	–2.037354104	0.243610107	–6.667936263	0.002141179	0.655933547	0.838734820	1.173881686	–1.165606558	–0.761927519	–0.741015977
17340272	17	Epcam	epithelial cell adhesion molecule	–4.952378438	0.032298737	–17.129961854	1.20E–05	0.888956142	0.920052173	0.922391405	–1.020627658	–0.794543062	–0.916228999
17466397	6	Epha1	Eph receptor A1	–2.297680706	0.203389808	–7.097050937	0.001452177	0.624566350	1.070449822	0.962384757	–0.649001693	–0.780779958	–1.227619279
17224661	1	Epha4	Eph receptor A4	–2.178861877	0.220849906	–7.813288032	0.000884288	0.855458351	1.046142101	0.815492749	–1.029789621	–0.954975508	–0.732328072
17431588	4	Ephb2	Eph receptor B2	–2.315509527	0.200891785	–7.960808627	0.000805618	0.828084428	0.924067026	0.951586475	–1.172537058	–0.827762672	–0.703438199
17230484	1	Ephx1	epoxide hydrolase 1, microsomal	3.685588138	12.866860168	11.343914730	0.000112080	–1.126830681	–0.748435286	–0.830234731	0.751172464	1.035679245	0.918648988
17246231	10	Erbb3	v-erb-b2 erythroblastic leukemia viral oncogene homolog 3 (avian)	–2.626090084	0.161982506	–9.164024379	0.000341990	0.690826240	0.953234444	1.071792075	–0.844591055	–0.960336296	–0.910925407
17299196	14	Ero1l	ERO1-like (S. cerevisiae)	2.210117976	4.627131103	4.323674435	0.017269963	–1.604943408	–0.610569497	–0.127962397	1.056220038	0.856786429	0.430468835
17423291	4	Esrp1	epithelial splicing regulatory protein 1	–4.287257566	0.051216144	–15.613509641	1.87E–05	0.895114307	0.948546549	0.890980899	–0.959602483	–0.820572919	–0.954466353
17512634	8	Esrp2	epithelial splicing regulatory protein 2	–2.099683211	0.233309473	–6.158078511	0.003286963	0.824589067	0.584296848	1.204061432	–1.265737881	–0.771857156	–0.575352310
17239719	10	Eya4	eyes absent 4 homolog (Drosophila)	–2.092723230	0.234437745	–7.098182804	0.001452177	0.715837370	0.795322428	1.178510054	–0.709591044	–1.023785922	–0.956292887
17357444	19	Fads3	fatty acid desaturase 3	–3.357119532	0.097590226	–12.204613587	7.22E–05	0.903877461	0.985933482	0.842035726	–0.976514189	–0.939877652	–0.815454828
17357815	19	Fam111a	family with sequence similarity 111, member A	4.480098234	22.317418222	14.941532175	2.22E–05	–1.029232501	–0.829419989	–0.867180845	0.924399775	0.791987885	1.009445674
17372552	2	Fam171b	family with sequence similarity 171, member B	2.820506909	7.064105597	7.593359601	0.001036782	–1.252896957	–0.463308938	–0.918991382	1.014318831	0.929985713	0.690892732
17443901	5	Fam20c	family with sequence similarity 20, member C	2.041794670	4.117574264	5.353912724	0.006810633	–1.307302927	–0.861003377	–0.364181053	0.890258783	1.192588619	0.449639955
17528924	9	Fam83b	family with sequence similarity 83, member B	–3.991397639	0.062873782	–13.074888314	5.26E–05	0.821820830	0.924819391	0.973318736	–1.076394258	–0.901447896	–0.742116803
17358797	19	Fas	Fas (TNF receptor superfamily member 6)	2.231574674	4.696463101	4.947690093	0.009562990	–1.545727819	–0.697257327	–0.206150117	0.680336587	0.718752710	1.050045966

17262964	11	Fat2	FAT tumor suppressor homolog 2 (Drosophila)	2.811022903	7.017819797	10.046494576	0.000207942	–0.979382848	–0.900011324	–0.845397114	0.894299082	1.058373442	0.772118761
17490589	7	Fcgrt	Fc receptor, IgG, alpha chain transporter	3.130110630	8.755020944	11.458814280	0.000109232	–0.840901471	–0.965144591	–0.926139890	0.943817576	0.977018968	0.811349407
17392056	2	Fermt1	fermitin family homolog 1 (Drosophila)	–5.121796533	0.028720078	–15.728728977	1.87E–05	1.023313235	0.808241691	0.889532328	–1.069346407	–0.807404537	–0.844336310
17305662	14	Fermt2	fermitin family homolog 2 (Drosophila)	2.499606276	5.655310655	8.350913518	0.000594774	–0.935891992	–0.862349787	–0.900551772	1.050093078	1.056366869	0.592333603
17324332	16	Fetub	fetuin beta	2.496470502	5.643031888	7.112402793	0.001439454	–0.987440551	–0.845571503	–0.802139630	0.401152519	1.188562590	1.045436575
17541926	X	Fgf13	fibroblast growth factor 13	–2.018387990	0.246833824	–7.476139207	0.001125507	0.929134023	0.963407611	0.836034340	–0.794234195	–1.030239550	–0.904102228
17447831	5	Fgfbp1	fibroblast growth factor binding protein 1	–5.440047240	0.023034702	–18.530795080	1.13E–05	0.878006481	0.942407861	0.911103429	–0.833119773	–0.863127667	–1.035270332
17539536	X	Figf	c-fos induced growth factor	2.710367892	6.544885223	7.181737508	0.001356945	–1.124706854	–1.075284994	–0.420335598	0.612070997	1.078076763	0.930179686
17256365	11	Fkbp10	FK506 binding protein 10	4.051749189	16.584334195	14.995026157	2.22E–05	–0.919750058	–0.915277380	–0.900988470	0.844378685	0.980645831	0.910991392
17386770	2	Fkbp7	FK506 binding protein 7	2.826225374	7.092161425	9.493647479	0.000271849	–1.050737158	–0.637417725	–1.020661151	0.910156650	0.930377790	0.868281594
17455346	5	Flt1	FMS-like tyrosine kinase 1	2.967701592	7.822889529	10.509741783	0.000163914	–0.966681846	–1.008609215	–0.748844510	0.889414035	0.998928779	0.835792757
17321524	15	Fmnl3	formin-like 3	2.594369404	6.039250034	8.568010069	0.000522982	–0.875309706	–1.117276709	–0.705203531	1.094066602	0.750540901	0.853182443
17229020	1	Fmo1	flavin containing monooxygenase 1	4.521384141	22.965306621	13.902608409	3.71E–05	–0.761254522	–1.030348823	–0.924745578	0.735062170	1.008719346	0.972567407
17544025	X	Fndc3c1	fibronectin type III domain containing 3C1	–2.030457305	0.244777473	–7.167783911	0.001368679	1.050605813	0.969546616	0.686287738	–0.777943682	–1.065416936	–0.863079549
17266424	11	Foxn1	forkhead box N1	–3.425049304	0.093101660	–12.271868927	7.12E–05	0.822738882	0.861453044	1.045511483	–0.857520248	–0.932267088	–0.939916073
17309981	15	Fyb	FYN binding protein	3.567296042	11.853950573	10.316043661	0.000177746	–1.232370045	–0.768013871	–0.689502719	0.882781319	0.859466970	0.947638347
17311179	15	Fzd6	frizzled homolog 6 (Drosophila)	–2.263508746	0.208264847	–6.641635849	0.002184549	0.972461284	0.716657984	0.940564709	–1.314024947	–0.836599633	–0.479059397
17261608	11	Gabrp	gamma-aminobutyric acid (GABA) A receptor, pi	–2.593826964	0.165645743	–5.538839083	0.005719168	0.351693205	0.573797282	1.566937343	–0.756045331	–0.947750340	–0.788632159
17251222	11	Gas7	growth arrest specific 7	2.078180569	4.222743361	6.462721713	0.002544156	–0.905325785	–1.017271357	–0.721450004	0.965389128	0.462412864	1.216245155
17371201	2	Gca	grancalcin	–2.438435138	0.184483649	–8.377392601	0.000589060	0.738655797	0.983824895	0.984372967	–1.123258820	–0.761061704	–0.822533136

17528562	9	Gcnt3	glucosaminyl (N-acetyl) transferase 3, mucin type	–3.490397551	0.088978615	–12.332820903	7.00E–05	0.795685740	1.034723670	0.897339411	–0.88505567	–0.991811268	–0.850881884
17363470	19	Gda	guanine deaminase	2.829519576	7.108373934	9.562654810	0.000264744	–1.065146489	–0.954872732	–0.690518408	1.016208427	0.780794199	0.913535003
17510114	8	Gdf15	growth differentiation factor 15	3.041945494	8.236009500	5.857162814	0.004275341	–0.891759005	–0.671115284	–0.935546597	1.450611110	0.859983345	0.187826431
17536600	X	Gdpd2	glycerophosphodiester phosphodiesterase domain containing 2	2.333258435	5.039422569	7.774760632	0.000906140	–1.115769454	–0.757666863	–0.818573984	1.127145971	0.692772093	0.872092237
17483098	7	Gdpd3	glycerophosphodiester phosphodiesterase domain containing 3	–2.957974901	0.128694749	–10.331693055	0.000177746	0.940864603	0.765509631	1.014468353	–1.052461182	–0.824191462	–0.844189943
17301823	14	Gfra2	glial cell line derived neurotrophic factor family receptor alpha 2	–3.624117673	0.081102058	–13.709139000	3.92E–05	0.898836281	0.906580813	0.932983183	–0.907361064	–0.922248376	–0.908790837
17393264	2	Ggt7	gamma-glutamyltransferase 7	2.527029941	5.763838613	9.514022404	0.000271849	–0.941596236	–0.897795325	–0.897458286	0.968055369	0.868896346	0.899898132
17307117	14	Gjb2	gap junction protein, beta 2	–3.070809195	0.119012978	–7.861906191	0.000854367	0.937294266	0.713952907	0.981623938	–1.340093266	–0.555071043	–0.737706802
17413500	4	Glipr2	GLI pathogenesis-related 2	3.342261512	10.141938400	12.243383885	7.12E–05	–1.012124178	–0.854314161	–0.866644765	0.895159284	0.872128754	0.965795065
17406165	3	Glrb	glycine receptor, beta subunit	2.268109732	4.816915887	6.274984343	0.003024898	–1.334235284	–0.440342038	–0.824226284	0.614217102	0.994710233	0.989876271
17439054	5	Gm1045	predicted gene 1045	–2.055373785	0.240586270	–6.153018308	0.003300148	0.685681618	0.734933642	1.199514659	–1.22186852	–0.498696555	–0.899564844
17268088	11	Gm11543	predicted gene 11543	–2.998173697	0.125158337	–7.344262202	0.001220649	0.902815814	0.856359854	0.851316871	–0.419822070	–0.820506315	–1.370164154
17416009	4	Gm12794	predicted gene 12794	–2.026782906	0.245401691	–6.472208872	0.002528262	0.896157075	1.000769066	0.757503566	–0.474137238	–0.990375560	–1.189916909
17401293	3	Gm15472	predicted gene 15472	–3.823893119	0.070614432	–9.701732448	0.000245044	0.858441200	0.658031455	1.150281518	–0.588429628	–1.017254991	–1.061069554
17548692	8	Gm19958	predicted gene, 19958	–4.708647857	0.038243335	–15.596404610	1.87E–05	0.771287867	0.942094846	1.012947770	–0.840652939	–1.001668530	–0.884009013
17548644	4	Gm3579	predicted gene 3579	–5.451424008	0.022853770	–17.736928433	1.13E–05	0.893807027	0.912854431	0.921363581	–0.956896891	–1.017465216	–0.753662931
17279934	12	Gm4755	predicted gene 4755	–2.423311033	0.186427807	–5.968102381	0.003882258	0.973492190	0.256105211	1.322941438	–0.928294379	–0.645188797	–0.979055663
17525482	9	Gm5615	predicted gene 5615	–2.486471645	0.178442152	–6.663556702	0.002146345	0.349855643	1.193012162	1.062519827	–0.979789920	–0.664302433	–0.961295281
17219039	1	Gpa33	glycoprotein A33 (transmembrane)	–2.982037553	0.126566056	–9.806147012	0.000235185	0.650612142	0.945227060	1.110611787	–0.973247911	–0.849525913	–0.883677165
17458439	6	Gpnmb	glycoprotein (transmembrane) nmb	2.103451123	4.297361439	6.070280308	0.003545868	–1.106122280	–1.022827941	–0.474900675	0.891436830	0.506961228	1.205452838
17405471	3	Gpr87	G protein-coupled receptor 87	–2.000942428	0.249836743	–7.213538206	0.001329358	0.812553408	1.085414201	0.817737910	–0.817509374	–1.056433538	–0.841762607
17311104	15	Grhl2	grainyhead-like 2 (Drosophila)	–2.809979615	0.142597479	–8.893102012	0.000406950	0.678706058	0.883744443	1.128657291	–1.113077027	–0.773923201	–0.804107564
17312280	15	Gsdmd	gasdermin D	2.282244986	4.864343085	6.146520924	0.003319335	–1.266285181	–0.740012002	–0.579088223	0.439448280	0.978303258	1.167633869

17211405	1	Gsta3	glutathione S-transferase, alpha 3	2.265432109	4.807984045	5.906699065	0.004106237	–0.905378875	–0.937552564	–0.721306806	0.228772928	1.194793641	1.140671678
17457876	6	Gstk1	glutathione S-transferase kappa 1	2.248516363	4.751939154	7.019932785	0.001560286	–0.724704578	–1.189274757	–0.745111334	0.694362857	0.766701596	1.198026216
17409142	3	Gstm2	glutathione S-transferase, mu 2	3.76769339	13.620364337	13.561607514	4.22E–05	–0.966385439	–0.861973164	–0.903519879	0.800368727	0.985732474	0.945777282
17325069	16	Heg1	HEG homolog 1 (zebrafish)	3.470560170	11.085179063	11.236495730	0.000117069	–1.026707385	–1.026780226	–0.658260365	0.838379005	0.918620711	0.954748259
17276328	12	Hif1a	hypoxia inducible factor 1, alpha subunit	2.065080481	4.184573190	6.916723867	0.001717038	–1.057735718	–0.833365326	–0.791027780	1.176912946	0.899255365	0.605960512
17519550	9	Hmgcll1	3-hydroxymethyl–3-methylglutaryl-Coenzyme A lyase-like 1	2.441819963	5.433267067	8.507736263	0.000542557	–0.784226097	–1.155383033	–0.772197544	0.880816973	0.944565862	0.886423839
17415570	4	Hook1	hook homolog 1 (Drosophila)	–2.971397806	0.127502920	–9.044379885	0.000367007	0.997712930	0.853552103	0.833650210	–0.969798849	–0.566248124	–1.148868270
17450142	5	Hpse	heparanase	2.283341005	4.868039940	7.436278783	0.001153401	–1.194847547	–0.923588354	–0.561836128	0.822040834	0.902645431	0.955585764
17340845	17	Igf2r	insulin-like growth factor 2 receptor	–3.011950221	0.123968869	–9.888212717	0.000222185	0.838466953	0.770961620	1.097232620	–1.026829944	–0.710734198	–0.969097051
17260474	11	Igfbp3	insulin-like growth factor binding protein 3	–2.460441295	0.181690980	–5.723738587	0.004810872	1.252061967	0.348840091	0.923146299	–1.081499028	–1.073650815	–0.368898515
17256264	11	Igfbp4	insulin-like growth factor binding protein 4	3.489830783	11.234241239	12.039064535	7.90E–05	–1.003501183	–0.793311532	–0.926872852	0.930536327	0.772158081	1.020991159
17315305	15	Igfbp6	insulin-like growth factor binding protein 6	3.805866843	13.985567033	12.714052738	6.01E–05	–0.810813520	–0.895089634	–1.015307025	0.747106497	0.943252074	1.030851607
17219554	1	Igsf9	immunoglobulin superfamily, member 9	–2.165082861	0.222969323	–6.562907667	0.002359580	0.965863221	0.553271466	1.118691614	–1.189345999	–0.572687355	–0.875792946
17461684	6	Il17re	interleukin 17 receptor E	–3.020160476	0.123265376	–10.593138861	0.000156631	0.799627732	0.827723375	1.095147745	–0.943533963	–0.847610344	–0.931354545
17212229	1	Il18r1	interleukin 18 receptor 1	–4.293371395	0.050999560	–15.190254713	2.08E–05	0.794039980	1.006239926	0.931284652	–0.941542295	–0.871571032	–0.918451231
17391554	2	Il1a	interleukin 1 alpha	–6.159738035	0.013987306	–17.069424309	1.20E–05	0.805559606	0.957749676	0.955158290	–1.094304897	–0.862113911	–0.762048764
17367659	2	Il1f6	interleukin 1 family, member 6	2.718215677	6.580584245	7.482611193	0.001125507	–1.014598742	–0.878115722	–0.744239288	0.440623919	0.957579977	1.238749856
17367652	2	Il1f9	interleukin 1 family, member 9	4.120059679	17.388477083	9.958573651	0.000215399	–1.222815051	–0.758497998	–0.684035204	0.757113654	1.137964040	0.770270560
17246091	10	Il23a	interleukin 23, alpha subunit p19	–2.441209333	0.184129242	–7.528025134	0.001087581	0.795526577	0.940664806	0.929434420	–1.286121812	–0.609708560	–0.76979543

17226708	1	Il24	interleukin 24	–3.338513721	0.098856955	–8.774961372	0.000450487	1.014017726	1.146181897	0.496283963	–1.069543171	–0.727801334	–0.859139081
17358598	19	Il33	interleukin 33	3.134995776	8.784716790	8.814485482	0.000436145	–1.223337224	–0.631392393	–0.812938175	0.787995060	0.771907511	1.107765222
17435725	5	Il6	interleukin 6	2.267154645	4.813728067	7.198473776	0.001339277	–1.173570666	–0.974295151	–0.520248988	0.873441775	0.775895856	1.018777173
17245399	10	Irak3	interleukin–1 receptor-associated kinase 3	3.088275494	8.504789294	9.502022851	0.000271849	–1.079945311	–0.891019232	–0.720750730	0.634224106	1.001856256	1.055634911
17527661	9	Islr	immunoglobulin superfamily containing leucine-rich repeat	2.373683814	5.182627893	6.907989300	0.001729244	–0.752795683	–0.664407123	–1.217939076	0.604205788	1.216802219	0.814133875
17238605	10	Itga7	integrin alpha 7	2.95878772	7.774703840	7.923694629	0.000824838	–0.925779232	–1.009314022	–0.707142319	0.963249160	1.218189079	0.460797333
17257235	11	Itgb3	integrin beta 3	2.641672337	6.240546335	8.084502126	0.000731926	–1.065815585	–0.997717861	–0.609641701	1.077119469	0.975580633	0.620475045
17258473	11	Itgb4	integrin beta 4	–2.964494360	0.128114497	–9.785267936	0.000237796	0.838116879	0.728029950	1.140902680	–0.782511949	–0.933753843	–0.990783718
17385654	2	Itgb6	integrin beta 6	–2.724538226	0.151297680	–9.759828346	0.000240068	1.006595926	0.735157891	0.982761448	–0.861147713	–0.893982619	–0.969384933
17322163	15	Itgb7	integrin beta 7	4.293870508	19.614797120	13.933834126	3.71E–05	–0.906429016	–0.862385174	–0.952453304	0.758913029	0.866843412	1.095511053
17303722	14	Kcnk5	potassium channel, subfamily K, member 5	2.328224488	5.021869324	8.574587898	0.000522982	–0.830112958	–0.969109428	–0.929880627	1.017454320	0.914889957	0.796758736
17511927	8	Kifc3	kinesin family member C3	–2.004581783	0.249207297	–6.076017733	0.003540398	0.650198030	0.815944737	1.155918042	–0.622037714	–0.693129582	–1.306893513
17471828	6	Klra2	killer cell lectin-like receptor, subfamily A, member 2	2.877387445	7.348182436	7.463586467	0.001128754	–1.092209890	–0.505976627	–1.026261255	1.010634914	1.102096324	0.511716534
17269391	11	Krt14	keratin 14	–3.269364313	0.103710631	–7.658203082	0.000989648	0.849075281	0.765450589	0.998806405	–1.399610987	–0.612849549	–0.600871738
17315245	15	Krt18	keratin 18	–2.394967206	0.190126666	–6.379209267	0.002756150	0.884784475	0.716007052	0.991779267	–0.827174478	–0.393650855	–1.371745461
17315200	15	Krt7	keratin 7	–4.598959457	0.041264373	–11.777880176	8.94E–05	0.917003665	0.903062714	0.869906913	–1.123352657	–0.562207768	–1.004412868
17321823	15	Krt80	keratin 80	–3.214607989	0.107722537	–9.120071008	0.000349986	1.175656517	0.695361432	0.802583398	–1.158449449	–0.744998978	–0.770152920
17217639	1	Lad1	ladinin	–3.190940075	0.109504338	–10.316737754	0.000177746	0.882233628	0.935729172	0.888632116	–1.074482839	–0.628973947	–1.003138129
17348492	18	Lama3	laminin, alpha 3	–3.804761455	0.071557091	–14.026233886	3.60E–05	0.891041246	0.921218641	0.922907004	–0.919626069	–0.984705546	–0.830835276
17232593	10	Lama4	laminin, alpha 4	3.936294388	15.308854051	13.854016904	3.74E–05	–0.981767105	–0.946531209	–0.801248161	0.901169941	0.999703681	0.828672852
17228136	1	Lamc2	laminin, gamma 2	–2.954123534	0.129038767	–9.37321407	0.000298250	0.803614898	1.100028569	0.792591026	–0.884586212	–0.690868106	–1.120780176
17526956	9	Layn	layilin	4.593430808	24.141289056	16.376179639	1.52E–05	–0.887552365	–1.010786271	–0.834839770	0.903655436	0.956199246	0.873323724
17378827	2	Lbp	lipopolysaccharide binding protein	5.560284024	47.185903562	19.681094035	1.13E–05	–1.001541768	–0.901837278	–0.831052980	0.927470550	0.893529604	0.913431872
17383892	2	Lcn2	lipocalin 2	2.408456143	5.309058883	5.507972200	0.005902005	–1.477019141	–0.826346724	–0.201556176	0.791623696	0.839664282	0.873634062
17310044	15	Lifr	leukemia inhibitory factor receptor	4.337710347	20.219989554	12.258576862	7.12E–05	–0.828344011	–0.930569022	–0.942783637	1.074727526	1.014099822	0.612869322
17233226	10	Lilrb4	leukocyte immunoglobulin-like receptor, subfamily B, member 4	2.433816825	5.403210263	7.517830390	0.001092913	–0.722319673	–1.067019074	–0.876560527	0.727341492	0.683501489	1.255056293

17364114	19	Lipa	lysosomal acid lipase A	2.241563768	4.729093835	6.551957564	0.002384151	–1.350175891	–0.781839573	–0.493640600	0.902564680	0.754480427	0.968610958
17355443	18	Lipg	lipase, endothelial	–2.279846543	0.205919657	–6.242507911	0.003110267	0.427012173	1.109254749	1.058141583	–0.707147041	–0.638167112	–1.249094351
17358749	19	Lipn	lipase, family member N	4.325658215	20.051777353	11.375664844	0.000110941	–0.908034522	–1.112836859	–0.668021074	0.719805919	0.854075224	1.115011313
17400622	3	Lix1l	Lix1-like	2.228101633	4.685170769	6.533390118	0.002405423	–1.213256914	–0.447169606	–0.965628465	0.760130881	1.129142245	0.736781858
17461477	6	Lmcd1	LIM and cysteine-rich domains 1	3.357530835	10.249849596	8.412894197	0.000578366	–0.938623708	–0.864264446	–0.839582890	1.206682999	1.018148545	0.417639501
17299105	14	LOC100505112	disks large homolog 5-like	–2.249165722	0.210345707	–8.021784070	0.000764834	0.922636805	0.955802616	0.837873806	–0.684672429	–0.998620543	–1.033020255
17455704	5	LOC625240	PRAME family member 12-like	–2.956184767	0.128854536	–9.502703844	0.000271849	0.611280662	1.105092735	0.983485191	–0.873359117	–0.972649127	–0.853850343
17354367	18	Lox	lysyl oxidase	5.245583005	37.938296753	17.367724602	1.20E–05	–0.877599064	–0.981180290	–0.869889421	0.773613460	0.957539023	0.997516292
17480485	7	Lrrc32	leucine rich repeat containing 32	3.899883653	14.927323994	12.823082504	5.68E–05	–0.966226292	–0.878278628	–0.875149571	1.064657486	0.941830316	0.713166690
17439901	5	Lrrc8b	leucine rich repeat containing 8 family, member B	–2.101385699	0.233034312	–7.362051965	0.001217620	1.018107457	0.755644629	0.931361084	–1.140804256	–0.787603952	–0.776704962
17318083	15	Ly6a	lymphocyte antigen 6 complex, locus A	2.928687292	7.614172697	5.960794944	0.003906531	–1.540986800	–0.444308725	–0.528759497	0.695475682	0.834922332	0.983657008
17318100	15	Ly6c2	lymphocyte antigen 6 complex, locus C2	3.435407059	10.818338643	9.128296094	0.000349986	–1.189165789	–0.889323272	–0.585461556	0.790138752	0.746749533	1.127062332
17312229	15	Ly6f	lymphocyte antigen 6 complex, locus F	2.027171110	4.076048196	4.153201511	0.020477239	–0.369176854	–1.232875192	–0.727230153	–0.041172396	1.227893783	1.142560811
17318013	15	Lynx1	Ly6/neurotoxin 1	2.501194237	5.661538829	6.813198474	0.001890217	–1.221026093	–0.810639549	–0.583070785	0.760230500	0.599807751	1.254698175
17279724	12	Macc1	metastasis associated in colon cancer 1	–5.862009627	0.017193301	–18.113332930	1.13E–05	0.917288557	0.859445302	0.948945791	–1.077127201	–0.810642357	–0.837910093
17311512	15	Mal2	mal, T cell differentiation protein 2	–3.854297350	0.069141832	–9.037782766	0.000367191	0.851771738	0.826252357	0.968997792	–0.888922343	–0.480806573	–1.277292971
17431332	4	Man1c1	mannosidase, alpha, class 1C, member 1	2.056503125	4.159768163	6.358559046	0.002792513	–0.940996405	–1.261810937	–0.436097767	0.815485422	0.863070803	0.960348884
17257906	11	Map2k6	mitogen-activated protein kinase kinase 6	2.511480806	5.702050459	8.478476396	0.000550161	–1.173075985	–0.690902926	–0.838769163	0.892506866	0.842045041	0.968196166
17231925	10	Map7	microtubule-associated protein 7	–2.446995581	0.183392230	–8.436809460	0.000569089	0.760246415	0.969383257	0.978681338	–0.972069807	–0.677965584	–1.058275619
17295685	13	Marveld2	MARVEL (membrane-associating) domain containing 2	–2.970733635	0.127561632	–10.66329305	0.000152970	1.035713309	0.759124892	0.932351493	–0.904562605	–0.875748282	–0.946878807

17512860	8	Marveld3	MARVEL (membrane-associating) domain containing 3	–2.266390950	0.207849193	–7.944514760	0.000814130	0.797319907	0.900330915	1.012304770	–1.045320051	–0.673062459	–0.991573083
17329403	16	Masp1	mannan-binding lectin serine peptidase 1	2.025030990	4.070006194	7.228146380	0.001320777	–0.952444175	–0.979219125	–0.779896225	1.021277292	0.680508863	1.009773370
17444970	5	Medag	mesenteric estrogen dependent adipogenesis	2.474214092	5.556645073	8.081825555	0.000731926	–1.153689234	–0.589723286	–0.946378747	0.867764939	0.831361088	0.990665240
17315002	15	Mettl7a1	methyltransferase like 7A1	2.086623709	4.247528739	6.687725023	0.002103516	–1.275820266	–0.562860298	–0.822223168	0.792637655	0.988614588	0.879651489
17222332	1	Mgat4a	mannoside acetylglu-cosaminyltransferase 4, isoenzyme A	–2.630818890	0.161452436	–8.579131112	0.000522982	0.857955678	0.87562294	0.96117209	–1.001595970	–0.585078071	–1.108076667
17397475	3	Mgst2	microsomal glutathione S-transferase 2	3.355481994	10.235303621	10.978593026	0.000130106	–1.136509398	–0.718730594	–0.856843147	0.847597026	0.954799545	0.909686568
17291026	13	Mir1983	microRNA 1983	–2.270138104	0.207310041	–5.423914211	0.006325166	1.229188269	1.095745657	0.184674763	–1.124322236	–0.816584984	–0.568701469
17255064	11	Mmd	monocyte to macrophage differentiation-associated	2.250807089	4.759490323	7.538204620	0.001087581	–0.982654297	–0.644985550	–1.063160436	0.723340555	0.862478862	1.104980866
17514482	9	Mmp13	matrix metallopeptidase 13	2.826763463	7.094807117	7.452502848	0.001138370	–1.185078715	–0.757589852	–0.684599115	0.467071708	1.051895097	1.108300876
17503825	8	Mmp2	matrix metallopeptidase 2	5.057650233	33.304615773	17.223402079	1.20E–05	–0.942979425	–0.895830926	–0.891575187	1.012718766	0.938147872	0.779518899
17514515	9	Mmp3	matrix metallopeptidase 3	2.406931513	5.303451271	5.768568670	0.004606168	–1.113295903	–0.580225320	–0.840477207	0.394982296	0.699506140	1.439509994
17232215	10	Moxd1	monooxygenase, DBH-like 1	–3.463955998	0.090624441	–11.115257805	0.000126154	0.780720832	1.101460698	0.827689379	–0.898940661	–0.756533746	–1.054396502
17516699	9	Mpzl2	myelin protein zero-like 2	–5.505301398	0.022016037	–15.204965022	2.08E–05	0.931905857	1.004001736	0.777197660	–1.010345796	–0.694851340	–1.007908117
17516707	9	Mpzl3	myelin protein zero-like 3	–3.597685570	0.082601651	–11.963270394	8.12E–05	0.859498568	0.933906293	0.924953666	–1.122400972	–0.808413421	–0.787544134
17228353	1	Mr1	major histocompatibility complex, class I-related	5.078085439	33.779719395	13.011983343	5.27E–05	–1.129309323	–0.642109725	–0.927187117	0.974942428	0.953096671	0.770567066
17224125	1	Mreg	melanoregulin	2.008454856	4.023510661	6.320590952	0.002881796	–0.807375191	–0.846789825	–0.990462885	1.326920254	0.555942496	0.761765152
17357700	19	Ms4a4d	membrane-spanning 4-domains, subfamily A, member 4D	3.965525715	15.622199832	11.893425157	8.43E–05	–0.939239090	–0.741616174	–1.024681711	0.717829100	1.110243069	0.877464806

17342340	17	Msln	mesothelin	–3.223940989	0.107027913	–10.387923330	0.000172657	0.707659874	1.146392313	0.852406856	–1.000460574	–0.819324506	–0.886673962
17374156	2	Muc15	mucin 15	–2.528056059	0.173372134	–5.128065236	0.008248509	0.079117777	0.959832293	1.407763827	–0.913353044	–0.967742380	–0.565618473
17351760	18	Myo5b	myosin VB	–2.286777241	0.204932792	–7.889103888	0.000841782	0.715906906	0.843258897	1.145068772	–1.029754196	–0.799337237	–0.875143142
17316402	15	Nipal2	NIPA-like domain containing 2	–4.934181799	0.032708699	–17.667346512	1.13E–05	0.936752110	0.814200065	0.983302958	–0.906359990	–0.896623078	–0.931272064
17526695	9	Nnmt	nicotinamide N-methyltransferase	2.501643572	5.663302423	8.374942488	0.000589060	–1.116337144	–0.612547569	–0.97069104	0.968117639	0.873158753	0.858299361
17253707	11	Nos2	nitric oxide synthase 2, inducible	2.747677371	6.716349800	5.581925875	0.005481326	–1.489625212	–0.831271895	–0.165531066	0.808936632	0.863603557	0.813887984
17410435	3	Npnt	nephronectin	–3.625855402	0.081004429	–12.853211853	5.66E–05	0.929685841	0.899519899	0.899834430	–0.947423610	–0.764009418	–1.017607144
17316043	15	Npr3	natriuretic peptide receptor 3	–2.884218810	0.135445202	–9.313766203	0.000310937	1.060434697	0.923324446	0.715475211	–0.665682991	–1.005684618	–1.027866745
17512732	8	Nqo1	NAD(P)H dehydrogenase, quinone 1	2.990511145	7.947555271	8.287587676	0.000621880	–0.921098728	–0.840088537	–0.894911886	0.443159835	1.109652196	1.103287118
17236604	10	Ntn4	netrin 4	2.078963536	4.225035714	5.636677573	0.005234003	–1.203401706	–0.706451654	–0.651585797	0.336692859	0.970817126	1.253929172
17219185	1	Olfml2b	olfactomedin-like 2B	2.027552456	4.077125755	6.651382244	0.002165474	–0.888993688	–1.169231198	–0.610922536	0.639111860	1.001304265	1.028731298
17318428	15	Oplah	5-oxoprolinase (ATP-hydrolysing)	2.418649268	5.346701994	6.754254277	0.001989968	–1.285333596	–0.938723429	–0.395083051	0.997864997	0.792970310	0.828304770
17414729	4	Orm1	orosomucoid 1	3.486247057	11.206369456	6.480478482	0.002526782	–1.016564719	–1.157008395	–0.359885685	0.684997822	0.523740768	1.324720209
17274021	12	Osr1	odd-skipped related 1 (Drosophila)	–2.856582854	0.138064771	–9.758119961	0.000240068	0.779005129	0.955143874	0.979731166	–0.890460803	–0.730562644	–1.092856722
17233536	10	P4ha1	procollagen-proline, 2-oxoglutarate 4-dioxygenase (proline 4-hydroxylase), alpha 1 polypeptide	2.075368691	4.214521056	5.833450558	0.004338383	–1.319769637	–0.890513680	–0.373836646	1.023299123	0.953929459	0.606891381
17358690	19	Papss2	3′-phosphoadenosine 5′-phosphosulfate synthase 2	2.606020405	6.088219632	5.528958321	0.005784151	–0.862310492	–0.748382602	–0.879685277	0.099442138	1.030663475	1.360272757
17382224	2	Pax8	paired box gene 8	4.002067424	16.022944905	12.161730033	7.33E–05	–0.946492374	–1.011061268	–0.750792881	1.052715568	0.968261828	0.687369126
17472085	6	Pbp2	phosphati-dylethanolamine binding protein 2	–2.348565561	0.196341145	–7.054033228	0.001512994	0.658658314	1.246918325	0.742446253	–1.018064896	–0.596690409	–1.033267586
17454082	5	Pcolce	procollagen C-endopeptidase enhancer protein	2.616393868	6.132153743	8.694808717	0.000473753	–0.989260873	–1.049909073	–0.661318970	0.805238186	1.084217606	0.811033124
17438246	5	Pdgfra	platelet derived growth factor receptor, alpha polypeptide	3.274381044	9.675800637	10.056460419	0.000207942	–0.978476499	–0.846275389	–0.871590613	1.207357832	0.728985834	0.759998835

17432440	4	Pdpn	podoplanin	2.908298831	7.507324434	10.390219585	0.000172657	–1.018711998	–0.932610449	–0.774283303	0.989652746	0.809795713	0.926157291
17251527	11	Per1	period circadian clock 1	2.382259021	5.213524548	5.625721242	0.005264697	–1.207253243	–0.687797833	–0.625953593	1.372408059	0.833638701	0.314957908
17231844	10	Perp	PERP, TP53 apoptosis effector	–2.336372906	0.198007515	–7.838159579	0.000868584	0.865972231	0.855979420	0.972880888	–1.157175792	–0.611278129	–0.926378617
17282664	12	Pgf	placental growth factor	2.089434055	4.255810918	4.871234857	0.010285913	–1.264100454	–0.369419760	–0.821806716	0.456402785	1.437674597	0.561249549
17399845	3	Pglyrp3	peptidoglycan recognition protein 3	–2.582092192	0.166998588	–7.627597980	0.001006983	0.841292981	0.545232611	1.269930566	–1.026662251	–0.820996642	–0.808797264
17211305	1	Pi15	peptidase inhibitor 15	3.073409808	8.417604974	9.581604270	0.000262032	–0.751855199	–1.159700395	–0.783243634	1.085034854	0.763852559	0.845911815
17227384	1	Pkp1	plakophilin 1	–2.976102162	0.127087834	–9.256495538	0.000323310	0.749265505	0.849068538	1.092965176	–1.165951748	–0.749925955	–0.775421516
17450121	5	Plac8	placenta-specific 8	2.614289938	6.123217536	9.587946518	0.000262032	–0.864731417	–1.034042793	–0.831141682	0.92217493	0.841307351	0.966433611
17297537	14	Plau	plasminogen activator, urokinase	–2.484574121	0.178677005	–7.347954789	0.001220649	0.680229994	0.970488917	0.999969078	–0.616956490	–0.745910915	–1.287820585
17340050	17	Plekhh2	pleckstrin homology domain containing, family H (with MyTH4 domain) member 2	3.14600705	8.852022082	10.018058114	0.000207942	–0.712316470	–0.865440823	–1.124269168	0.725668366	0.983381584	0.992976510
17394297	2	Pltp	phospholipid transfer protein	2.402910223	5.288689301	7.892187831	0.000841782	–1.122463004	–0.956740225	–0.609872528	0.967953002	0.728812247	0.992310507
17544220	X	Pof1b	premature ovarian failure 1B	–3.917738904	0.066167248	–13.719255006	3.92E–05	0.811305841	0.871816660	1.045663980	–0.873282861	–0.973771318	–0.881732301
17397575	3	Postn	periostin, osteoblast specific factor	–6.711497072	0.009541970	–22.905820017	1.13E–05	0.847910108	0.972061634	0.914066641	–0.834018048	–0.948994641	–0.951025694
17416234	4	Ppap2b	phosphatidic acid phosphatase type 2B	2.318740493	4.988964808	7.210831523	0.001329358	–1.202482750	–0.913686809	–0.545708667	0.740236912	0.844393968	1.077247346
17447835	5	Prom1	prominin 1	–2.889523613	0.134948083	–6.164031645	0.003283713	0.427946287	0.802680146	1.303887047	–0.834864253	–0.442981243	–1.256667985
17391287	2	Prom2	prominin 2	–2.632149195	0.161303629	–9.854432139	0.000227134	0.932194373	0.869553354	0.93377962	–0.987117777	–0.868011244	–0.880398326
17326510	16	Pros1	protein S (alpha)	2.822061379	7.071721108	10.395054335	0.000172657	–0.987081358	–0.859267145	–0.885819629	0.846675080	1.006985286	0.878507766
17341671	17	Prss22	protease, serine, 22	–3.007498685	0.124351974	–8.498650331	0.000542557	0.517048463	1.067585146	1.078789661	–0.686541686	–0.945048240	–1.031833343
17357981	19	Prune2	prune homolog 2 (Drosophila)	2.477146186	5.567949728	6.014345100	0.003742169	–1.158029695	–1.005466672	–0.388216463	1.298281390	0.741197643	0.512233796
17336446	17	Psmb8	proteasome (prosome, macropain) subunit, beta type 8 (large multifunctional peptidase 7)	2.471407621	5.545846250	7.336734564	0.001222526	–1.334304035	–0.673673360	–0.643450103	0.876876852	0.822696306	0.951854341
17383712	2	Ptges	prostaglandin E synthase	3.385444726	10.450099157	12.016035558	7.92E–05	–1.017844116	–0.764475599	–0.945540287	0.857106955	0.912648056	0.958104991
17465856	6	Ptn	pleiotrophin	2.072222999	4.205341611	7.683783941	0.000974335	–0.811097505	–0.896213103	–1.022237031	0.833018218	0.976752347	0.919777073

17401269	3	Ptpn22	protein tyrosine phosphatase, non-receptor type 22 (lymphoid)	–2.499723758	0.176810547	–7.339713725	0.001222526	0.790847492	0.960014671	0.897768389	–1.278640852	–0.876809446	–0.493180254
17429057	4	Ptprf	protein tyrosine phosphatase, receptor type, F	–2.794569346	0.144128810	–9.245896208	0.000323310	0.863427780	0.773536742	1.066877269	–1.003199968	–0.669482879	–1.031158945
17398115	3	Ptx3	pentraxin related gene	3.443990003	10.882891407	8.527863396	0.000537334	–0.863671354	–1.018530991	–0.761094615	0.940681729	1.240582567	0.462032663
17219308	1	Pvrl4	poliovirus receptor-related 4	–2.076832113	0.237034324	–6.548011971	0.002389009	0.528729558	0.892801408	1.229905025	–1.084053193	–0.723822434	–0.843560364
17311853	15	Pvt1	plasmacytoma variant translocation 1	2.398630068	5.273022192	7.465413138	0.001128754	–0.980690052	–0.592447762	–1.093918941	1.167319610	0.790814873	0.708922272
17282074	12	Rab15	RAB15, member RAS oncogene family	–2.282985714	0.205472081	–8.434185068	0.000569089	0.900463328	0.968107030	0.861262857	–1.034303822	–0.886277722	–0.809251671
17406807	3	Rab25	RAB25, member RAS oncogene family	–5.093354308	0.029291902	–18.714937036	1.13E–05	0.906160904	0.938191722	0.892097183	–0.867365750	–0.891123331	–0.977960729
17426565	4	Rasef	RAS and EF hand domain containing	–2.667487429	0.157400559	–8.530506534	0.000537334	0.917444342	0.831327475	0.940763529	–1.095251947	–0.560807314	–1.033476086
17470060	6	Rassf4	Ras association (RalGDS/AF-6) domain family member 4	4.301101136	19.713351080	15.426404864	1.93E–05	–0.935500325	–0.842922625	–0.954638497	0.887706086	1.001085686	0.844269676
17448389	5	Rbm47	RNA binding motif protein 47	–2.399298778	0.189556682	–8.386080372	0.000589060	0.807672317	0.774336690	1.129971875	–0.996351083	–0.799405165	–0.916224634
17490578	7	Rcn3	reticulocalbin 3, EF-hand calcium binding domain	2.682124325	6.41800238	9.352484049	0.000302031	–0.947755567	–0.814536951	–0.954274737	0.989699733	1.032412144	0.694455378
17542501	X	Renbp	renin binding protein	2.030069284	4.084244639	7.530899265	0.001087581	–1.003223963	–0.927122312	–0.799025451	0.860805420	0.867995003	1.000571303
17538096	X	Rnf128	ring finger protein 128	2.641926216	6.241644615	8.313562563	0.000614458	–0.960854455	–1.156671574	–0.565550727	0.784429181	0.974438654	0.924208921
17544164	X	Rps6ka6	ribosomal protein S6 kinase polypeptide 6	–3.375347991	0.096364930	–10.017566008	0.000207942	0.944702449	0.973067901	0.772988069	–1.199029153	–0.665656859	–0.826072408
17240012	10	Rspo3	R-spondin 3 homolog (Xenopus laevis)	2.887825785	7.401541587	8.364395546	0.000590654	–1.203179126	–0.513218204	–0.949419119	0.991415338	0.799408420	0.874992690
17399776	3	S100a14	S100 calcium binding protein A14	–5.162289487	0.027925182	–16.426458141	1.52E–05	0.845393088	0.927111901	0.952277961	–1.053463923	–0.756599874	–0.914719153
17399823	3	S100a8	S100 calcium binding protein A8 (calgranulin A)	2.377722478	5.197156411	5.168383670	0.008005942	–0.821574116	–1.006203885	–0.637464915	0.065822362	1.091853634	1.307566922
17491193	7	Saa3	serum amyloid A 3	3.724280333	13.216610537	7.931898689	0.00082133	–1.234275915	–0.841252832	–0.534056269	0.557132282	1.209205941	0.843246793

17317031	15	Samd12	sterile alpha motif domain containing 12	–3.162140533	0.111712263	–11.411485383	0.000110445	0.799136133	0.935484637	0.994950353	–0.986135903	–0.916864754	–0.826570466
17301414	14	Scara5	scavenger receptor class A, member 5 (putative)	2.211485372	4.631518807	6.123310924	0.003401109	–1.276211389	–0.859464739	–0.456918545	0.584799351	1.185857800	0.821937523
17365098	19	Scd1	stearoyl-Coenzyme A desaturase 1	–4.604318782	0.041111369	–14.333063533	3.05E–05	0.766951289	0.946007833	1.005689677	–0.775516018	–1.061387258	–0.881745524
17385924	2	Scn3a	sodium channel, voltage-gated, type III, alpha	–2.582806743	0.166915896	–8.362646377	0.000590654	0.833616454	1.023614503	0.833457447	–0.767277214	–1.210880247	–0.712530944
17310837	15	Sema5a	sema domain, seven thrombospondin repeats (type 1 and type 1-like), transmembrane domain (TM) and short cytoplasmic domain, (semaphorin) 5A	–2.098678195	0.233472058	–7.589898617	0.001036782	0.946797667	0.982809435	0.789536479	–0.954000664	–1.034738986	–0.730403932
17278328	12	Serpina3n	serine (or cysteine) peptidase inhibitor, clade A, member 3N	4.365010337	20.606253715	12.546034936	6.27E–05	–0.957557141	–1.077955386	–0.669317711	0.750770833	0.965210485	0.988848919
17216402	1	Serpinb5	serine (or cysteine) peptidase inhibitor, clade B, member 5	–3.718497543	0.075966252	–10.306570926	0.000177746	0.949703350	0.902372559	0.832343702	–1.249056477	–0.698766948	–0.736596186
17224771	1	Serpine2	serine (or cysteine) peptidase inhibitor, clade E, member 2	2.969083929	7.830388721	8.522863121	0.000537334	–1.035930327	–1.012362841	–0.618413441	0.889574709	0.615534276	1.161597625
17387517	2	Serping1	serine (or cysteine) peptidase inhibitor, clade G, member 1	2.420229011	5.352559809	7.968386360	0.000804593	–1.038526849	–0.753515568	–0.898648115	0.608378370	1.104289385	0.978022777
17493658	7	Serpinh1	serine (or cysteine) peptidase inhibitor, clade H, member 1	5.010303205	32.229350404	16.381165710	1.52E–05	–0.882377255	–0.780717415	–1.063455972	0.870177189	0.946660438	0.909713015
17211043	1	Sgk3	serum/glucocorticoid regulated kinase 3	–2.530760773	0.173047407	–7.095776855	0.001452177	0.830068179	0.875447315	0.925172101	–1.378969352	–0.668752268	–0.582965976
17453714	5	Sh2b2	SH2B adaptor protein 2	2.249165548	4.754077917	7.231360379	0.001320777	–1.147168608	–0.704164653	–0.821429722	1.187779870	0.741751281	0.743231832
17501588	8	Sh2d4a	SH2 domain containing 4A	–3.096722550	0.116894378	–10.411984675	0.000172657	0.783919124	0.898885604	1.031135613	–1.092671546	–0.75564593	–0.865622866
17302523	14	Slain1	SLAIN motif family, member 1	–2.267518039	0.207686876	–7.271827812	0.001287311	0.921086009	0.997334375	0.754226654	–0.643252138	–0.774831109	–1.254563790

17377144	2	Slc24a3	solute carrier family 24 (sodium/ potassium/calcium exchanger), member 3	2.482162925	5.587345079	8.307626024	0.000615227	−1.018365847	−0.918261373	−0.762257239	0.905635606	0.661417733	1.131831121
17287803	13	Slc25a48	solute carrier family 25, member 48	−2.130515474	0.228376249	−7.362088315	0.001217620	1.082337358	0.851329399	0.766193780	−1.138082382	−0.811906642	−0.749871513
17359156	19	Slc35g1	solute carrier family 35, member G1	−2.269310879	0.207428944	−5.970087426	0.003882258	1.054113127	0.763997883	0.752004559	−1.420592862	−0.400324277	−0.749198430
17455386	5	Slc46a3	solute carrier family 46, member 3	2.470430137	5.542089991	8.155539654	0.000700707	−0.980500307	−0.807570802	−0.905481535	0.974577235	1.123412620	0.595562789
17314556	15	Slc48a1	solute carrier family 48 (heme transporter), member 1	2.016651438	4.046435049	7.235825391	0.001320777	−0.895227794	−0.709208492	−1.109381719	0.961160257	0.926970171	0.825687577
17377651	2	Slc52a3	solute carrier protein family 52, member 3	−2.239157385	0.211810001	−7.958811713	0.000805618	1.035790399	0.730044393	0.948941625	−1.061924972	−0.830877202	−0.821974243
17237035	10	Slc6a15	solute carrier family 6 (neurotransmitter transporter), member 15	−2.382896395	0.191724100	−7.672679278	0.000982078	1.000922763	0.953506106	0.725890677	−1.153056057	−0.945644519	−0.581618971
17394153	2	Slpi	secretory leukocyte peptidase inhibitor	3.234018472	9.408850470	10.730919564	0.000148913	−0.972306180	−1.042363705	−0.698481200	0.771857048	0.962043517	0.979250520
17272619	11	Socs3	suppressor of cytokine signaling 3	2.918227369	7.559167561	8.238182897	0.000647518	−0.837522580	−0.719698498	−1.101391275	1.123443446	1.019606573	0.515562334
17437385	5	Sod3	superoxide dismutase 3, extracellular	2.804512474	6.986221936	9.123250775	0.000349986	−1.01069485	−1.008917220	−0.679577135	0.783670518	0.799627312	1.115891376
17447404	5	Sorcs2	sortilin-related VPS10 domain containing receptor 2	−2.505655039	0.176085127	−8.764011106	0.000451699	0.829731990	0.773011341	1.111649732	−0.783027645	−0.962973407	−0.968392012
17449800	5	Sowahb	sosondowah ankyrin repeat domain family member B	−2.139895464	0.226896229	−7.470184852	0.001128736	0.677216347	0.951382146	1.076115698	−0.873909162	−0.776506354	−1.054298674
17399880	3	Sprr2g	small proline-rich protein 2G	2.466119157	5.525554146	5.400320576	0.006462653	−1.361901780	−0.930926555	−0.193909819	0.510150824	1.105117622	0.871469709
17397170	3	Spry1	sprouty homolog 1 (Drosophila)	−2.123500060	0.229489482	−5.305754669	0.007126760	0.879577996	0.69648899	0.937485415	−0.406810813	−0.564652248	−1.542089340
17356240	19	Sptbn2	spectrin beta, non-erythrocytic 2	−2.881272227	0.135722120	−8.497677828	0.000542557	0.788051652	0.624170633	1.259297022	−0.855314124	−0.801580905	−1.014624279
17375538	2	Sqrdl	sulfide quinone reductase-like (yeast)	3.087029198	8.497445468	9.501921141	0.000271849	−1.22324763	−0.758909213	−0.709627047	0.889319203	0.967652888	0.834811799
17241409	10	Srgn	serglycin	2.842826025	7.174240081	7.371473598	0.001213454	−1.338442426	−0.802494570	−0.480756380	1.039555803	0.815479049	0.766658525

17525240	9	St14	suppression of tumorigenicity 14 (colon carcinoma)	–2.877379880	0.136088788	–8.109138994	0.000727912	0.839411759	0.863300949	0.953115942	–1.026448533	–0.453696293	–1.175683825
17317675	15	St3gal1	ST3 beta-galactoside alpha–2,3-sialyltransferase 1	2.894456941	7.435640093	10.040963377	0.000207942	–0.984384470	–1.038746777	–0.695248220	0.951472670	0.849232014	0.917674783
17301594	14	Stc1	stanniocalcin 1	3.785459327	13.789128057	12.482408087	6.39E–05	–0.834473788	–1.030204416	–0.854284216	0.788929253	1.082505857	0.84752731
17330183	16	Stfa3	stefin A3	2.511805062	5.703332180	4.383078888	0.016422221	–0.772208141	–0.611981895	–0.945177575	0.241442294	0.373962041	1.713963276
17384457	2	Stom	stomatin	2.962707913	7.795858544	8.923773593	0.000398122	–1.205699352	–0.648009579	–0.827769427	1.029835851	0.917054664	0.734587843
17517848	9	Stra6	stimulated by retinoic acid gene 6	2.82628163	7.092437980	7.779048011	0.000906140	–0.668939631	–0.84609985	–1.129099151	0.633256094	0.741876905	1.269005634
17394538	2	Sulf2	sulfatase 2	2.825774434	7.089944992	8.291088552	0.000621880	–0.683113698	–1.259820220	–0.724261378	0.863257171	0.744894522	1.059043603
17490939	7	Sult2b1	sulfotransferase family, cytosolic, 2B, member 1	–2.530769758	0.173046329	–7.643564843	0.000997529	0.767479885	0.956651648	0.938166088	–1.297370831	–0.704292254	–0.660634536
17425816	4	Susd1	sushi domain containing 1	–2.343719906	0.197001715	–8.377930646	0.000589060	0.720099851	1.045766798	0.952995503	–0.975618919	–0.807019680	–0.936223553
17485200	7	Syt8	synaptotagmin VIII	–2.037900161	0.243517919	–7.240775578	0.001319353	0.853469007	0.750170303	1.106098764	–0.932756822	–0.753371755	–1.023609498
17480102	7	Sytl2	synaptotagmin-like 2	3.249063102	9.507480677	10.576272497	0.000156631	–0.948594420	–0.962250905	–0.798375805	0.825872598	1.143257377	0.740091156
17467359	6	Tacstd2	tumor-associated calcium signal transducer 2	–2.743151032	0.149358264	–6.453267156	0.002560144	0.785607744	0.624811606	1.157317962	–1.239475982	–0.321635209	–1.006626121
17257405	11	Tanc2	tetratricopeptide repeat, ankyrin repeat and coiled-coil containing 2	2.128474241	4.372548060	6.341134483	0.002831282	–0.895040669	–1.275218915	–0.454696935	1.016967277	0.664473161	0.943516081
17283364	12	Tc2n	tandem C2 domains, nuclear	–3.444812002	0.091835006	–11.570220610	0.000101818	0.756337449	0.948372852	1.013717357	–1.014804314	–0.951625683	–0.75199766
17259907	11	Tcn2	transcobalamin 2	2.264359486	4.804410709	7.178282816	0.001356945	–1.225804602	–0.855258917	–0.586163786	1.023458248	0.931182602	0.712586456
17400072	3	Tdrkh	tudor and KH domain containing protein	–2.220703458	0.214536726	–6.486103604	0.002525422	1.098080186	0.901362215	0.623813052	–1.279049592	–0.508991340	–0.835214521
17450618	5	Tgfbr3	transforming growth factor, beta receptor III	3.662866622	12.665802841	10.261231999	0.000181589	–0.843941891	–0.977599655	–0.863729865	1.189090502	0.910597134	0.585583775
17374488	2	Thbs1	thrombospondin 1	–2.098196546	0.233550017	–6.074420239	0.003540398	0.861226410	0.633636860	1.110260436	–0.546156474	–0.708426581	–1.350540651
17312191	15	Them6	thioesterase superfamily member 6	2.164706341	4.483751557	7.516702841	0.001092913	–0.884574662	–0.715466329	–1.103129860	0.790854746	0.824225419	1.088090686
17330478	16	Tigit	T cell immunoreceptor with Ig and ITIM domains	–3.356940988	0.097602304	–7.820603044	0.000881101	0.965605560	0.981548648	0.670237172	–1.360404748	–0.712877895	–0.544108736

17243406	10	Tle6	transducin-like enhancer of split 6, homolog of Drosophila E(spl)	2.114814195	4.331342311	7.245271293	0.001319353	–1.105708740	–0.882151381	–0.707474990	0.822832401	1.121332287	0.751170422
17509101	8	Tlr3	toll-like receptor 3	–3.549069290	0.085432613	–7.722435355	0.000942560	0.747226278	0.790160066	1.068168963	–1.159286856	–1.101633100	–0.344635352
17485553	7	Tmc4	transmembrane channel-like gene family 4	–4.105131142	0.058107527	–12.533173583	6.27E–05	0.695199276	0.943796189	1.071565216	–0.977967857	–0.765463302	–0.967129522
17255058	11	Tmem100	transmembrane protein 100	3.751867320	13.471768243	11.020113375	0.000128958	–0.768001249	–0.831595316	–1.098026505	0.705104399	1.120202001	0.872316670
17454500	5	Tmem184a	transmembrane protein 184a	–2.794026340	0.144183068	–9.101535203	0.000350540	0.817408462	0.747439889	1.134453411	–0.878183838	–0.742109596	–1.079008328
17418934	4	Tmem54	transmembrane protein 54	–4.670851014	0.039258503	–15.667652944	1.87E–05	0.958640778	0.908608514	0.860194544	–1.063750723	–0.831219909	–0.832473204
17449301	5	Tmprss11e	transmembrane protease, serine 11e	–6.249990195	0.013139096	–19.901000161	1.13E–05	0.929031861	0.802257713	0.997908663	–0.972482130	–0.812352630	–0.944363477
17332568	16	Tmprss2	transmembrane protease, serine 2	–2.891163721	0.134794756	–9.646334109	0.000253442	0.778277221	0.879141268	1.050916684	–0.957723670	–0.691710573	–1.058900929
17426365	4	Tnc	tenascin C	3.853118690	14.451213004	9.729062912	0.000244541	–0.803445334	–1.036433032	–0.826729756	1.232015185	0.875268322	0.559324616
17344309	17	Tnf	tumor necrosis factor	2.721978962	6.597772178	8.924141960	0.000398122	–0.839060521	–0.944089378	–0.916078248	1.139343906	0.939236457	0.620647786
17370735	2	Tnfaip6	tumor necrosis factor alpha induced protein 6	2.418078448	5.344586924	8.566152525	0.000522982	–0.784063009	–1.031965118	–0.900977568	0.784889002	1.079953938	0.852162756
17217651	1	Tnnt2	troponin T2, cardiac	–2.930814297	0.131140545	–8.734036153	0.000458608	0.607425841	1.120572809	0.948964338	–0.979508475	–1.041994366	–0.655460146
17338210	17	Trerf1	transcriptional regulating factor 1	2.000670917	4.001860609	7.230760439	0.001320777	–1.055811126	–0.801504903	–0.859587020	1.074162050	0.841862045	0.800878954
17241308	10	Tspan15	tetraspanin 15	2.684402138	6.428143510	9.600141279	0.000261203	–0.933613245	–0.875973090	–0.914057029	0.777336480	0.856470206	1.089836678
17544385	X	Tspan6	tetraspanin 6	–3.058150508	0.120061832	–11.226193011	0.000117069	0.950951757	0.83902638	0.942457208	–0.842925820	–0.882093604	–1.00741592
17533269	X	Tspan7	tetraspanin 7	–3.142198525	0.113267155	–10.992224821	0.000130091	0.839705197	1.038125260	0.845340563	–0.939917118	–0.770901995	–1.012351907
17400599	3	Txnip	thioredoxin interacting protein	3.859264397	14.512904744	4.500688338	0.014667483	–1.157139752	–0.497652096	–0.646203506	–0.051797424	1.512023500	0.840769277
17449649	5	U90926	cDNA sequence U90926	3.680001397	12.817130455	6.985019006	0.001605940	–0.745926892	–0.838962648	–0.976591014	0.229681768	1.080435263	1.251363523
17372604	2	Ube2l6	ubiquitin-conjugating enzyme E2L 6	2.187610204	4.555502511	5.849794184	0.004291224	–1.418188403	–0.571745924	–0.578743186	0.672419274	0.752094375	1.144163864
17413221	4	Unc13b	unc–13 homolog B (C. elegans)	–2.002077264	0.249640297	–7.400065420	0.001184290	0.890615723	0.808917275	1.027907937	–1.004597606	–0.897134646	–0.825708683
17356002	19	Unc93b1	unc–93 homolog B1 (C. elegans)	2.889483107	7.410049124	8.928243962	0.000398122	–1.235736774	–0.685046556	–0.765740322	0.840381167	0.982003660	0.864138824
17548193	2	Vim	vimentin	3.238928808	9.440928856	10.232355568	0.000183032	–0.972021002	–0.914493880	–0.815694237	1.172502296	0.698687294	0.831019529
17279980	12	Vsnl1	visinin-like 1	–5.085425799	0.029453323	–18.119971819	1.13E–05	0.894831735	0.926696548	0.912604681	–0.932895433	–0.987624977	–0.813612553
17420154	4	Wnt4	wingless-related MMTV integration site 4	–2.101856326	0.232958306	–7.629017703	0.001006983	0.741930401	0.980751806	0.998083997	–0.853142658	–1.032888157	–0.834735390

17469016	6	Wnt7a	wingless-related MMTV integration site 7A	–2.035025157	0.244003685	–6.092973988	0.003486265	0.711434324	1.298630022	0.607925371	–1.106383567	–0.567008427	–0.944597724
17395079	2	Zbp1	Z-DNA binding protein 1	2.746940519	6.712920322	4.861427753	0.010416704	–1.486134280	–0.583899973	–0.324228524	0.951955492	0.274500992	1.167806292
17535448	X	Zfp185	zinc finger protein 185	–2.355400720	0.195413126	–6.165583821	0.003283713	0.793632326	0.653204824	1.131548566	–1.403540640	–0.548613853	–0.626231223
17264053	11	Zfp287	zinc finger protein 287	2.142238498	4.414464674	7.556585595	0.001070382	–0.980569701	–0.814378045	–0.914378729	0.986440837	0.659019976	1.063865663
17486474	7	Zscan4b	zinc finger and SCAN domain containing 4B	–2.030202113	0.244820774	–6.007125825	0.003753263	0.793496371	0.875424610	0.941349754	–0.621594527	–0.573589000	–1.415087208
17486496	7	Zscan4e	zinc finger and SCAN domain containing 4E	–2.558027544	0.169807544	–8.740947462	0.000457996	0.776484047	0.86521794	1.066270224	–1.024730331	–0.991311263	–0.691930618

382 DE genes identified under the threshold of fold change (FC) of 4 or greater and significance less than 0.05.

Supplementary Table S2: Biological process gene ontology (GO) analysis of DE genes

Term	Genes Count	Overlapping Entities
cell adhesion	40	PKP1,DSG2,CDH17,FAT2,CDH10,PPAP2B,EPHA4,EPHA1,ITGB7,ITGA7,ITGB3,ITGB4,ITGB6,PVRL4,AEBP1,MPZL2,SORBS2,FERMT2,LAMA4,COL6A3,POSTN,CCL2,CTGF,THBS1,PTPRF,SEMA5A,HPSE,NPNT,PDPN,GPNMB,CDHR1,PERP,LAMC2,LAMA3,MSLN,FERMT1,MPZL3,ADAM12,TNFAIP6,ISLR
cell-cell signaling	12	GJB2,CCL2,CCL5,CCL7,FGF13,STC1,WNT7A,WNT4,SEMA5A,TNFAIP6,GDF15,FGFBP1
regulation of apoptotic process	11	PAX8,IGF2R,FAS,DAPK1,IL6,IRAK3,LCN2,CASP4,PSMB8,CASP1,DYNAP
cell migration	9	FLT1,PDGFRA,ITGA7,TGFBR3,CTGF,THBS1,MMP2,FMNL3,ST14
cell-cell adhesion	7	PKP1,CDH10,PPAP2B,PVRL4,MPZL2,PDPN,LAMA3
Apoptosis	7	FAS,GAS7,DNASE1L3,DAPK1,SH2B2,CASP4,AMOT
epithelial to mesenchymal transition	7	HIF1A,TGFBR3,WNT4,VIM,CDH1,KRT14,KRT18
response to oxidative stress	6	NQO1,SOD3,MMP2,LCN2,TXNIP,DGKK
cell-cell junction organization	5	CDH10,PVRL4,HEG1,MARVELD2,MARVELD3
canonical Wnt signaling pathway	5	PPAP2B,FZD6,WNT7A,WNT4,RSPO3
positive regulation of MAPK cascade	4	FLT1,IGFBP3,IL6,IGFBP4
non-canonical Wnt signaling pathway	3	FZD6,WNT7A,WNT4
vascular endothelial growth factor receptor signaling pathway	3	FLT1,CCL2,FIGF
positive regulation of reactive oxygen species metabolic process	3	AKR1C3,THBS1,PLAU
negative regulation of cell death	3	PTGES3,CCL5,CTGF
angiogenesis involved in wound healing	2	ITGB3,HPSE
Wnt signaling pathway involved in wound healing, spreading of epidermal cells	1	WNT7A
canonical Wnt signaling pathway involved in positive regulation of cell-cell adhesion	1	PPAP2B
non-canonical Wnt signaling pathway via MAPK cascade	1	WNT4
canonical Wnt signaling pathway involved in positive regulation of wound healing	1	PPAP2B

Many categories shared the same transcripts.

Supplementary Table S3: DE genes and their relation to some biological processes

Gene Symbol	Gene Description	Fold Change	Adjusted *P* Value
DE genes and their relation to cell migration			
Flt1	FMS-like tyrosine kinase 1	7.82	0.000164
Pdgfra	platelet derived growth factor receptor, alpha polypeptide	9.68	0.000208
Itga7	integrin alpha 7	7.77	0.000825
Tgfbr3	transforming growth factor, beta receptor III	12.67	0.000182
Ctgf	connective tissue growth factor	–12.99	0.000135
Thbs1	thrombospondin 1	–4.28	0.00354
Mmp2	matrix metallopeptidase 2	33.3	1.20E–05
Fmnl3	formin-like 3	6.04	0.000523
St14	suppression of tumorigenicity 14 (colon carcinoma)	–7.35	0.000728
DE genes and their relation to oxidative stress			
Nqo1	NAD(P)H dehydrogenase, quinone 1	7.95	0.000622
Sod3	superoxide dismutase 3, extracellular	6.99	0.00035
Mmp2	matrix metallopeptidase 2	33.3	1.20E–05
Lcn2	lipocalin 2	5.31	0.005902
Txnip	thioredoxin interacting protein	14.51	0.014667
Dgkk	diacylglycerol kinase kappa	–12.63	5.27E–05
DE genes and their relation to vasculogenesis			
Angpt1	angiopoietin 1	5.63	0.000906
Amot	angiomotin	–4.5	0.003788
Heg1	HEG homolog 1 (zebrafish)	11.09	0.000117
Tmem100	transmembrane protein 100	13.47	0.000129
DE genes and their relation to angiogenesis			
Hif1a	hypoxia inducible factor 1, alpha subunit	4.18	0.001717
Fzd6	frizzled homolog 6 (Drosophila)	–4.8	0.002185
Ephb2	Eph receptor B2	–4.98	0.000806
Epha1	Eph receptor A1	–4.92	0.001452
Flt1	FMS-like tyrosine kinase 1	7.82	0.000164
Ccl2	chemokine (C-C motif) ligand 2	8.92	0.000272
Ctgf	connective tissue growth factor	–12.99	0.000135
Angpt1	angiopoietin 1	5.63	0.000906
Figf	c-fos induced growth factor	6.54	0.001357
Plau	plasminogen activator, urokinase	–5.6	0.001221
Mmp2	matrix metallopeptidase 2	33.3	1.20E–05
Ecm1	extracellular matrix protein 1	4.35	0.002067
Tmem100	transmembrane protein 100	13.47	0.000129
DE genes and their relation to proteolysis			
Aebp1	AE binding protein 1	67.59	1.13E–05
Mmp2	matrix metallopeptidase 2	33.3	1.20E–05
Mmp13	matrix metallopeptidase 13	7.09	0.001138
Mmp3	matrix metallopeptidase 3	5.3	0.004606
Plau	plasminogen activator, urokinase	–5.6	0.001221
Casp4	caspase 4, apoptosis-related cysteine peptidase	4.09	0.021065
Casp1	caspase 1	79.7	1.13E–05

Adam12	a disintegrin and metallopeptidase domain 12 (meltrin alpha)	–7.14	0.000222
Cfb	complement factor B	4.64	0.000862
Psmb8	proteasome (prosome, macropain) subunit, beta type 8 (large multifunctional peptidase 7)	5.55	0.001223
Masp1	mannan-binding lectin serine peptidase 1	4.07	0.001321
Pros1	protein S (alpha)	7.07	0.000173
Pcolce	procollagen C-endopeptidase enhancer protein	6.13	0.000474
St14	suppression of tumorigenicity 14 (colon carcinoma)	–7.35	0.000728
Slpi	secretory leukocyte peptidase inhibitor	9.42	0.000149
Pi15	peptidase inhibitor 15	8.42	0.000262
Prss22	protease, serine, 22	–8.04	0.000543
Tmprss2	transmembrane protease, serine 2	–7.42	0.000253
Tmprss11e	transmembrane protease, serine 11e	–76.12	1.13E–05
DE genes and their relation to EMT			
Hif1a	hypoxia inducible factor 1, alpha subunit	4.18	0.001717
Tgfbr3	transforming growth factor, beta receptor III	12.67	0.000182
Wnt4	wingless-related MMTV integration site 4	–4.29	0.001006983
Vim	vimentin	9.44	0.000183032
Krt14	keratin 14	–9.64	0.000989648
Krt18	keratin 18	–4.61	0.002544156
Cdh1	cadherin 1	–28.48	1.13E–05

Supplementary Table S4: Interaction analysis of some molecular and biological processes in PLum-AD and PLum-AI cells

Relation	Type	Sentence	TextRef	Connectivity	# of References	Organ
KRT18 ---> apoptosis	Regulation	The KRT18 gene, which is involved in apoptosis, had reduced expression., The present study tested whether CK18 expression influences susceptibility to cytokine-induced apoptosis., A cytokeratin 18-Asp396 neoepitope elevation, indicative of chemotherapy-induced apoptosis, is linked to good prognosis., The cleavage of cytokeratin-18 (CK-18) by caspases is an early event in the apoptotic process., We have shown previously that a K8/K18 loss leads to a modulation of apoptosis in Fas-stimulated mouse hepatocytes. <more data available...>	info:pmid/22653643#abs:10, info:pmid/21119362#abs:3, info:pmid/16821621#abs:8, info:pmid/17847110#abs:2, info:pmid/18486629#abs:3, info:pmid/15194421#abs:4, info:pmid/22114688#abs:5, info:pmid/11684708#abs:9, info:pmid/17969036#abs:4, info:pmid/23167970#abs:8 <more data available...>	2	38	Retina {Organ urn: agi-ncimorgan: C1962966}, Liver {Organ urn: agi-ncimorgan: CL384198}, Heart {Organ urn: agi-ncimorgan: C1281570}, jejunum {Organ urn: agi-ncimorgan: C1278922}, Villus {Organ urn: agi-ncimorgan: C1519988}, Gland {Organ urn: agi-ncimorgan: C1285092}, head {Organ urn: agi-ncimorgan: C0018670}
ITGA7 ——> cell migration	Regulation	The Integrin a7 Cytoplasmic Domain Controls Integrin a7-mediated Cell Migration and Polarization., Forced expression of normal ITGA7 in prostate cancer and leiomyosarcoma cell lines suppressed tumor growth and cancer cell migration *in vitro*., The forced expression of normal ITGA7 in prostate cancer and leiomyosarcoma cell lines suppressed tumor growth and cancer cell migration *in vitro*.	info:pmid/11278916#body:210, info:pmid/20651226#body:13, info:pmid/23830872#body:12	2	3	
CLDN7 ---> apoptosis	Regulation	Additionally, EpCAM-CLDN7 complex was suggested to have role in migration, proliferation, apoptosis resistance, metastasis, and tumorigenicity [37], [39]., Furthermore, EpCAM is one of the surface markers in pancreatic cancer stem cells, and claudin-7 regulates the EpCAM-mediated functions in tumor progression, including those in pancreatic cancer, such as proliferation, migration, and anti-apoptosis.	info:pmid/21789222#cont:170, info:pmid/20566751#body:192	2	2	
ITGA7 ---> apoptosis	Regulation	In addition, site-directed protease-null mutant high temperature requirement A2S306A expression blocked apoptosis induced by ITGA7., In this study, expression of the a7 integrin transgene did suppress apoptosis in a7BX2- mdx/utr -/- mice compared to mdx/utr -/- mice., Concomitantly, integrin a7 silencing promoted apoptotic signaling and made airway smooth muscle cells refractory to the suppressive effects of serum deprivation on caspase 3 activity (Fig. 3C).	info:pmid/20651226#abs:7, info:pmid/21216283#body:150, info:pmid/23756649#cont:375	2	3	airway {Organ urn: agi-ncimorgan: C0458827}

TXNIP --+> apoptosis	Regulation	TIEG and VDUP1 play important roles in mediating apoptosis., Thioredoxin-interacting protein (Txnip) is a modulator of cellular redox state and contributes to cell apoptosis., Furthermore, the Itch-TXNIP interaction regulates intracellular reactive oxygen species levels and apoptosis., TXNIP promoted reactive oxygen species production and apoptosis by inhibiting thioredoxin., TXNIP increases the production of reactive oxygen species, and oxidative stress, resulting in cellular apoptosis., Overexpression of vitamin D(3)-up-regulated protein-1 but not of thioredoxin induced cardiomyocyte apoptosis. <more data available...>	info:pmid/15000885#abs:6, info:pmid/23305039#abs:2, info:pmid/20068034#abs:11, info:pmid/22869588#abs:9, info:pmid/22750447#abs:2, info:pmid/12011048#abs:7, info:pmid/16562669#abs:6, info:pmid/16529693#abs:6, info:pmid/18171713#abs:6, info:pmid/22474421#abs:1 <more data available...>	2	91	Heart {Organ urn: agi-ncimorgan: C1281570}, Islets of Langerhans {Organ urn: agi-ncimorgan: C0022131}, Breast {Organ urn: agi-ncimorgan: C0006141}, liver parenchyma {Organ urn: agi-ncimorgan: C0736268}, Ovary {Organ urn: agi-ncimorgan: CL384202}, Retina {Organ urn: agi-ncimorgan: C1962966}, Pancreas {Organ urn: agi-ncimorgan: C1278931}
THBS1 ---> cell migration	Regulation	Our results show that TSP1 is a potent stimulator of prostate tumor cell migration., TSP-1 stimulated cell migration at low concentrations (0.1–10 microg/ml) and was inhibitory at high concentrations (25–100 microg/ml)., In addition, thrombospondin-1 regulation in hypoxia proved to be important for cell renal carcinoma cell lines cell migration and invasion., Furthermore, we found that wtPTEN inhibited EGF—but not TSP-1—stimulated FTC-133 cell migration and also inhibited invasion induced by EGF and by TSP-1. <more data available...>	info:pmid/22037878#abs:4, info:pmid/12814189#abs:8, info:pmid/23145312#abs:7, info:pmid/15707585#abs:7, info:pmid/21142150#abs:1, info:pmid/15371459#abs:2, info:pmid/10454737#abs:2, info:pmid/17106256#abs:2, info:pmid/10479655#body:203, info:pmid/19129184#body:301 <more data available...>	2	46	Ovary {Organ urn: agi-ncimorgan: CL384202}, Bladder {Organ urn: agi-ncimorgan: C1281573}, Lung {Organ urn: agi-ncimorgan: C1278908}, Endometrium {Organ urn:agi-ncimorgan: C1550633}, Dermis {Organ urn: agi-ncimorgan: C0011646}, Eye {Organ urn: agi-ncimorgan: C1550636}, Renal Tubule {Organ urn: agi-ncimorgan: CL355448}
CLDN7 ---> cell invasion	Regulation	We demonstrate that knockdown of claudin-7 leads to reduced E-cadherin expression, impairment of homotypic adhesion, and increased cell invasion., Knockdown of claudin-7 expression in esophageal squamous cell carcinoma cells induces loss of E-cadherin, along with increased cell growth and enhanced cell invasion [84].	info:pmid/17255337#body:23, info:pmid/24009024#cont:393	2	2	Esophagus {Organ urn:agi-ncimorgan: C1278919}, Ovary {Organ urn: agi-ncimorgan: CL384202}
CASP4 --+> apoptosis	Regulation	Overexpression of ICH-2 in insect cells induces apoptosis., Caspase-11 plays a crucial role in both inflammation and apoptosis., Inhibition of JNK, caspase-4, or caspase-8 activation diminished edelfosine-induced apoptosis., Furthermore, we show that caspase-11-mediated apoptosis under septic condition is Bid-independent., Furthermore, CHOP siRNA or inhibition of caspase-4 activity attenuated withaferin A-induced apoptosis., Furthermore, the inhibition of caspase-4 activity by z-LEVD-fmk significantly reduced resveratrol-induced apoptosis. <more data available...>	info:pmid/7797510#abs:4, info:pmid/11684090#abs:1, info:pmid/17974980#abs:7, info:pmid/12232800#abs:5, info:pmid/21266191#abs:8, info:pmid/17914584#abs:5, info:pmid/17854271#abs:7, info:pmid/16465268#abs:5, info:pmid/24178758#abs:7, info:pmid/24394318#abs:11 <more data available...>	2	100	Gastrula {Organ urn: agi-ncimorgan: C0017199}, Synovial Membrane {Organ urn: agi-ncimorgan: C0039099}, Retina {Organ urn: agi-ncimorgan: C1962966}, Dental Pulp {Organ urn: agi-ncimorgan: C0011399}, Kidney {Organ urn: agi-ncimorgan: C1278978}, retinal pigment epithelium {Organ urn: agi-ncimorgan: C0035322}, airway {Organ urn: agi-ncimorgan: C0458827}

THBS1 --+> apoptosis	Regulation	At day 5, TSP-1 also induced promyelocytic leukemia cell apoptosis., Growth of the cells on a fibronectin substrate also suppressed induction of apoptosis by TSP1 or the peptides., TSP-1-mediated apoptosis was consistently associated with the up-regulation of active Caspase-3., Thrombospondin-1 (TSP-1) is a multifunctional protein known to modulate angiogenesis, endothelial cell adhesion and apoptosis., Knockdown of TSP-1, through RNA interference, resulted in overexpression of VEGF and reduced cytokine-induced apoptosis. <more data available...>	info:pmid/15868907#abs:5, info:pmid/9135017#abs:9, info:pmid/14612980#abs:5, info:pmid/12128069#abs:1, info:pmid/17154366#abs:7, info:pmid/16406676#abs:4, info:pmid/20080874#abs:10, info:pmid/8696941#abs:3, info:pmid/16962673#abs:5, info:pmid/18539706#abs:6 <more data available...>	2	100	Ovary {Organ urn: agi-ncimorgan: CL384202}, Blood capillaries {Organ urn: agi-ncimorgan: C0006901}, Ovarian Follicle {Organ urn: agi-ncimorgan: C1283799}, Dermis {Organ urn: agi-ncimorgan: C0011646}, Microvessels {Organ urn: agi-ncimorgan: C2350570}, Intestines {Organ urn: agi-ncimorgan: C0021853}, Urinary tract {Organ urn: agi-ncimorgan: C0042027}, Brain {Organ urn: agi-ncimorgan: C1269537}, Blood Vessels {Organ urn: agi-ncimorgan: C0005847}, Heart {Organ urn: agi-ncimorgan: C1281570} <more data available...>
ST14 ---> cell invasion	Regulation	Overexpression of bikunin significantly reduced the gene expression of matriptase, which attenuated *in vitro* cell invasion in the PC-3 cells., Suppression of matriptase expression by small hairpin RNA knockdown in ErbB-2-overexpressing LNCaP cells dramatically suppressed cancer cell invasion., Two other members of the TTSP family, matriptase and hepsin, are over-expressed in prostate adenocarcinoma and mechanistically influence cancer cell invasion and metastasis., Matriptase inhibition reduced cancer cell invasion in matrigels. <more data available...>	info:pmid/18649735#abs:6, info:pmid/20971737#abs:9, info:pmid/18338334#abs:2, info:pmid/23044656#cont:50, info:pmid/12571229#body:294, info:pmid/23466486#cont:217, info:pmid/20652801#cont:186, info:pmid/19853659#body:20, info:doi/10.2353/ajpath.2010.100228#body:214	2	9	Aorta {Organ urn: agi-ncimorgan: C1278934}, Prostate {Organ urn: agi-ncimorgan: C1278980}
FLT1 --+> apoptosis	Regulation	Transfection of the sflt-1 gene also attenuated pulmonary fibrosis and apoptosis at 14 days., Knocking down expression of VEGFR1 and inhibiting activation of NFkappaB and JNK also blocked lysophosphatidic acid induced protection against apoptosis., In addition, intra-amniotic sFlt-1 treatment suppressed activation of lung VEGF receptor-2 and increased apoptosis in endothelial and mesenchymal cells in the newborn lung., In turn, FLT-1 neutralization affects leukemia localization (now in the bone marrow diaphysis), increases leukemia apoptosis, and impedes the exit of acute lymphoblastic leukemia cells, prolonging the survival of inoculated mice. <more data available...>	info:pmid/16002726#abs:6, info:pmid/18396013#abs:9, info:pmid/22003089#abs:7, info:pmid/16249383#abs:5, info:pmid/19608702#body:116, info:pmid/22796052#body:235, info:pmid/22503073#body:105, info:pmid/21225468#cont:273, info:pmid/14521839#body:39, info:pmid/23975597#cont:327 <more data available...>	2	31	amnion {Organ urn: agi-ncimorgan: C0002630}, Diaphyses {Organ urn: agi-ncimorgan: C0242696}, Alveolus {Organ urn: agi-ncimorgan: C1515933}, Lung {Organ urn: agi-ncimorgan: C1278908}, Aorta {Organ urn: agi-ncimorgan: C1278934}, nucleus pulposus {Organ urn: agi-ncimorgan: C1185714}, Left ventricular {Organ urn: agi-ncimorgan: C0225897}, Liver {Organ urn: agi-ncimorgan: CL384198}

FLT1 ---> epithelial to mesenchymal transition	Regulation	This effect is reversed with exogenous recombinant mouse VEGF-A165, whereas addition of soluble VEGF receptor-1 blocks epithelial-mesenchymal transformation., VEGFR-1 on tumor cells may contribute to the aggressive behavior of hepatocellular carcinoma cells by inducing epithelial to mesenchymal transition., VEGFR-1 was reported to have a function in eliciting epithelial-to-mesenchymal transition in cancer cells toward invasive and metastatic phenotype., These data suggest that VEGFR-1 may be involved in the regulation of Epithelial-mesenchymal transition. <more data available...>	info:pmid/12591918#abs:4, info:pmid/21345006#abs:8, info:pmid/19689271#abs:4, info:pmid/23776453#cont:464, info:pmid/21336677#cont:321, info:pmid/16397214#title:1, info:pmid/22912411#cont:523, info:pmid/23384563#body:221, info:pmid/22579961#body:167, info:doi/10.1016/j. amjsurg.2007.05.007#body:87	2	10	Endocardium {Organ urn: agi-ncimorgan: C1550632}, Blood Vessels {Organ urn: agi-ncimorgan: C0005847}
PDGFRA --+> cell migration	Regulation	The PDGF alpha-receptor also mediates a mitogenic signal, but fails to induce cell migration in certain cell types., Activation of the platelet-derived growth factor alpha receptor (alphaPDGFR) leads to cell migration and DNA synthesis., According to our data, mole ovotestes result from a process of PDGFRalpha-mediated mesonephric cell migration, which occurs simultaneously in both sexes., PDGFRA, a receptor whose activity is required for cell migration through the primitive streak, is a target of miR-130b and -218 *in vivo*. <more data available...>	info:pmid/8617789#abs:2, info:pmid/10497192#abs:1, info:pmid/19382159#abs:8, info:pmid/22995917#abs:8, info:pmid/22070644#abs:6, info:pmid/21996738#cont:434, info:pmid/22080864#cont:55, info:pmid/12176024#body:111, info:pmid/21331762#cont:154, info:pmid/12651897#body:152 <more data available...>	2	21	Primitive Streak {Organ urn: agi-ncimorgan: C0033153}, Gonadal {Organ urn: agi-ncimorgan: C0018067}, Spinal Cord {Organ urn: agi-ncimorgan: CL366046}, Neural Crest {Organ urn: agi-ncimorgan: C0027789}, Epicardium {Organ urn: agi-ncimorgan: C0225968}, Mesenchyme {Organ urn: agi-ncimorgan: C0162415}, Mesonephric {Organ urn: agi-ncimorgan: C0025492}, Brain {Organ urn: agi-ncimorgan: C1269537}
EGLN3 --+> apoptosis	Regulation	EGLN3 also has pro-apoptotic activity in some cell types., PHD3 may be an important regulator of apoptosis and it is mainly found in tumors with good prognosis., EglN2 and EglN3 also appear to play Hypoxia-inducible factor-independent roles in regulating cell proliferation and apoptosis, respectively., One isoform, PHD3, is expressed in response to hypoxia and causes apoptosis in oxygenated conditions in neural cells., Finally, NF1 loss is highlighted as a valuable study model to investigate the cell lineage selectivity of the Egln3-mediated developmental apoptotic defect of chromaffin precursor cells. <more data available...>	info:pmid/19737309#abs:2, info:pmid/21877141#abs:11, info:pmid/22089927#abs:6, info:pmid/18337469#abs:2, info:pmid/19391076#abs:7, info:pmid/20973793#abs:6, info:pmid/20849813#abs:6, info:pmid/22898032#abs:5, info:pmid/19691672#abs:9, info:pmid/20978507#cont:587 <more data available...>	2	45	Neural Crest {Organ urn: agi-ncimorgan: C0027789}, Heart {Organ urn: agi-ncimorgan: C1281570}, Nervous system {Organ urn: agi-ncimorgan: C0027763}, Nerve {Organ urn: agi-ncimorgan: C1280541}, Breast {Organ urn: agi-ncimorgan: C0006141}, superior cervical ganglion {Organ urn: agi-ncimorgan: C1281048}

PDGFRA ---> apoptosis	Regulation	Thus, our findings show that PDGFRalpha signaling through PLCgamma and phosphatidylinositol 3-kinase has a protective role in preventing apoptosis in early development., Pdgfra is expressed in the mesenchyme of multiple organs during embryonic development and Pdgfralpha is involved in cell proliferation, differentiation, migration, and apoptosis in many tissues., Although PDGFR-A inhibition by this agent resulted in reduced mouse tumor cell growth and increased apoptosis *in vitro*, and reduced tumor cell proliferation *in vivo*, tandutinib did reduce tumor volume at the doses tested (360 mg/kg) *in vivo*. <more data available...>	info:pmid/15919820#abs:8, info:pmid/15811134#abs:1, info:pmid/22146535#abs:5, info:pmid/21659960#cont:148, info:pmid/17141222#body:100, info:pmid/23861062#cont:356, info:pmid/17599807#body:18, info:pmid/24287421#body:100, info:pmid/23805317#cont:197, info:pmid/21996738#cont:292 <more data available...>	2	19	Mesoderm {Organ urn: agi-ncimorgan: C0025485}, Mesenchyme {Organ urn: agi-ncimorgan: C0162415}, Lung {Organ urn: agi-ncimorgan: C1278908}, Fetus {Organ urn: agi-ncimorgan: C1305737}, Liver {Organ urn: agi-ncimorgan: CL384198}, Brain {Organ urn: agi-ncimorgan: C1269537}, Gastrointestinal tract {Organ urn: agi-ncimorgan: C0017189}
LCN2 --+> apoptosis	Regulation	Our results also revealed that NGAL protect cells from apoptosis., It has been shown that over-activation of NGAL induces apoptosis., Thus 24p3 is an MK886-inducible gene and may play an important role in MK886-induced apoptosis., This study provides evidence that overexpression of 24p3 gene can induce apoptosis of mammary epithelial cells., Although previous publications have suggested that 24p3 has pro-apoptotic functions, other data are more suggestive of a survival function., In addition, our experiments with caspase inhibitors LEHD-FMK and IETD-FMK prevent LCN2-induced apoptosis. <more data available...>	info:pmid/18662586#abs:12, info:pmid/23114405#abs:6, info:pmid/12614196#abs:13, info:pmid/15055523#abs:7, info:pmid/16060857#abs:3, info:pmid/22707293#abs:8, info:pmid/18317594#abs:8, info:pmid/19229297#abs:3, info:pmid/23941109#abs:4, info:pmid/16157692#abs:4 <more data available...>	2	88	Fetus {Organ urn: agi-ncimorgan: C1305737}, Sebaceous Glands {Organ urn: agi-ncimorgan: C0036505}, Renal Tubule {Organ urn: agi-ncimorgan: CL355448}, Mammary Gland {Organ urn: agi-ncimorgan: C0929301}, Uterus {Organ urn: agi-ncimorgan: C1269032}, Pancreas {Organ urn: agi-ncimorgan: C1278931}, Brain {Organ urn: agi-ncimorgan: C1269537}, Bone Marrow {Organ urn: agi-ncimorgan: C0005953}, Hippocampus {Organ urn: agi-ncimorgan: C0019564}, Kidney Glomerulus {Organ urn: agi-ncimorgan: C0022663} <more data available...>
DNASE1L3 --+> apoptosis	Regulation	Furthermore, DNase gamma is suggested to be involved in naturally occurring apoptosis in developing nervous systems., These findings suggest that DNase gamma plays an important role in Fas-independent apoptotic DNA fragmentation in hepatocytes., However, overexpression of DNaseY alone neither triggered apoptosis nor facilitated cell death in response to VM26 or serum deprivation., These results demonstrate that DNase-gamma gene transfer can induce apoptosis in human glioma cells, indicating its potential to become a future gene therapy strategy. <more data available...>	info:pmid/15167901#abs:7, info:pmid/15118903#abs:8, info:pmid/15002038#abs:4, info:pmid/12814251#abs:7, info:pmid/14646507#abs:1, info:pmid/12050166#abs:11, info:pmid/12802584#abs:7, info:pmid/15036412#abs:7, info:pmid/10807908#abs:5, info:pmid/14556310#abs:9 <more data available...>	2	17	Nervous system {Organ urn: agi-ncimorgan: C0027763}, Liver {Organ urn: agi-ncimorgan: CL384198}, Skin {Organ urn: agi-ncimorgan: C0684084}

CASP4 ---> cell migration	Regulation	Here, we show that caspase-11, a mammalian pro-inflammatory caspase, regulates cell migration during inflammation., Caspase-3 and caspase- 11 may promote cell migration [27], [28]., Recently, it has been shown that caspase-11 has non-pathological function of regulating cell migration., Caspase-11 plays diverse roles in apoptosis, inflammation, and cell migration., Caspase-11 regulates cell migration by promoting Aip1-Cofilin-mediated actin depolymerization., Unlike caspase 1, caspase 11 mediates the lipopolysaccharide neurotoxicity [26] and regulates cell migration [27]. <more data available...>	info:pmid/17293856#abs:3, info:pmid/19675677#body:318, info:pmid/21530491#body:109, info:pmid/18474237#body:15, info:pmid/18309324#body:231, info:pmid/24037645#cont:60, info:pmid/22582393#cont:316, info:pmid/18411310#body:280, info:pmid/18332114#body:255	2	9	
THBS1 --+> cell invasion	Regulation	TSP-1 up-regulated KB tumor cell invasion 5-fold., Stromal TSP-1 may play a role in regulating tumor cell invasion., Tumor cell invasion was upregulated 3.5 to 4.5-fold by TSP-1 and TGF-beta 1, respectively., Antibodies against uPA or uPAR neutralized the TSP-1- and TGF-beta 1-promoted breast tumor cell invasion., TSP-1 promotes tumor cell invasion of collagen by breast cancer cells., HGF stimulation of cell invasion and enhancement of MMP-9 expression were partially suppressed by TSP-1 overexpression., We previously showed that thrombospondin 1 (TSP-1) upregulates the plasminogen/plasmin system and promotes breast tumor cell invasion. <more data available...>	info:pmid/15172186#abs:9, info:pmid/12219015#abs:2, info:pmid/9695745#abs:9, info:pmid/9288157#abs:9, info:pmid/8661169#abs:12, info:pmid/19959938#abs:4, info:pmid/10090848#abs:1, info:pmid/19634159#abs:13, info:pmid/18661355#abs:4, info:pmid/10917542#abs:9 <more data available...>	2	41	Ground substance {Organ urn: agi-ncimorgan: C1383501}, Bladder {Organ urn: agi-ncimorgan: C1281573}, Aorta {Organ urn: agi-ncimorgan: C1278934}, Salivary Glands {Organ urn: agi-ncimorgan: C0036098}, Skin {Organ urn: agi-ncimorgan: C0684084}, Blood Vessels {Organ urn: agi-ncimorgan: C0005847}, Dermis {Organ urn: agi-ncimorgan: C0011646}
NQO1 ---> apoptosis	Regulation	Mammalian NAD(P)H:quinone oxidoreductases such as human NQO1 act as inducers of apoptosis., NAD(P) H:quinoneoxidoreductase 1 (NQO1) inhibits some cancers and increases p53 and apoptosis in cells., NQO1 participates in cellular defense against oxidative stress and regulates apoptosis via p53- and NF?B-mediated pathways., NQO1 activity, therefore, is a key determinant of beta-Lapachone-mediated apoptosis and cytotoxicity in prostate cancer cells., beta-Lapachone, an o-naphthoquinone, induces a novel caspase- and p53-independent apoptotic pathway dependent on NAD(P)H:quinone oxidoreductase 1 (NQO1). <more data available...>	info:pmid/19709309#abs:1, info:pmid/18676018#abs:1, info:pmid/21706157#abs:1, info:pmid/11412042#abs:9, info:pmid/15837761#abs:1, info:pmid/21947872#abs:7, info:pmid/22687461#abs:8, info:pmid/23831944#abs:7, info:pmid/24189322#abs:7, info:pmid/11279125#abs:1 <more data available...>	2	45	Kidney {Organ urn: agi-ncimorgan: C1278978}, Breast {Organ urn: agi-ncimorgan: C0006141}, Colon {Organ urn: agi-ncimorgan: C1281569}, Bone Marrow {Organ urn: agi-ncimorgan: C0005953}, Skin {Organ urn: agi-ncimorgan: C0684084}, Brain {Organ urn: agi-ncimorgan: C1269537}
ST14 ---> epithelial to mesenchymal transition	Regulation	These results suggest that epithin is a key mediator of TGF-beta-induced epithelial-mesenchymal transition in tumor progression., suppressor of tumorigenicity 14 has also been reported to inhibit ZEB2-driven epithelial to mesenchymal transition.	info:pmid/20398629#abs:8, info:pmid/20980099#body:199	2	2	Mesenchyme {Organ urn: agi-ncimorgan: C0162415}

FLT1 --+> cell migration	Regulation	Blockade of a VEGFR-1 autocrine loop with PTK787/ ZK222584 inhibited tumor cell migration., VEGFR-1/Flt-1, a VEGF receptor that regulates cell migration and blood vessel formation, was also down-regulated in AhR-/- cells., We demonstrate further that FLT-1-induced cell migration involves actin polymerization and lipid raft formation., Further, VEGF stimulation resulted in enhanced cellular migration, which was effectively blocked by pharmacologic inhibition of VEGFR-1 or Src kinase., Our laboratory has shown that vascular endothelial growth factor receptor-1 (VEGFR-1) expression on human pancreatic cancer cell lines mediates cell migration and invasion. <more data available...>	info:pmid/18316624#abs:8, info:pmid/15946950#abs:7, info:pmid/16249383#abs:6, info:pmid/16685275#abs:5, info:pmid/16397214#abs:1, info:pmid/9924144#abs:5, info:pmid/17420287#abs:11, info:pmid/15107818#abs:3, info:pmid/21345805#abs:3, info:pmid/21752276#abs:5 <more data available...>	2	56	Lymphatic System {Organ urn: agi-ncimorgan: C0024235}, Diaphyses {Organ urn: agi-ncimorgan: C0242696}, Colon {Organ urn: agi-ncimorgan: C1281569}, Heart {Organ urn: agi-ncimorgan: C1281570}, Blood Vessels {Organ urn: agi-ncimorgan: C0005847}, Prostate {Organ urn: agi-ncimorgan: C1278980}, Endometrium {Organ urn: agi-ncimorgan: C1550633}, Bone Marrow {Organ urn: agi-ncimorgan: C0005953}, Retina {Organ urn: agi-ncimorgan: C1962966}, Mammary Gland {Organ urn: agi-ncimorgan: C0929301}
ST14 --+> cell migration	Regulation	The serine protease MT-SP1/matriptase plays an important role in cell migration and matrix degradation., Furthermore, TGF-beta-induced cell migration and invasion were significantly impaired by epithin knockdown., These data suggest that matriptase might be a central regulator of cell migration and cancer invasion., These studies demonstrated that matriptase, likely its protease activity, is critical for neural progenitor cell migration., These results further support our working hypothesis that matriptase is an upstream regulator of cellular migration and extracellular matrix degradation. <more data available...>	info:pmid/17255532#abs:1, info:pmid/20398629#abs:6, info:pmid/11864986#body:284, info:pmid/21149451#cont:296, info:pmid/10962009#body:216, info:pmid/12571229#body:289, info:pmid/11567025#body:63	2	7	Mesenchyme {Organ urn: agi-ncimorgan: C0162415}
PAX8 ---| apoptosis	Regulation	Suppression of PAX8 selectively induces apoptotic cell death of ovarian cancer cells., The Pax-8 gene may play a crucial role in heart development and regulating cardiocyte apoptosis., The anti-apoptotic b cell lymphoma 2 (BCL2) gene is associated with treatment resistance, and is transactivated by the paired box transcription factor 8 (PAX8)., Remarkably, tp53inp1 silencing significantly abolishes Pax8-induced apoptosis thus suggesting that tp53inp1 may be the mediator of the observed effects., Furthermore, the inactivation of Pax8 in adult mouse thyroid induces apoptosis of TCF and reveals a significant up-regulation of genes involved in ... <more data available...>	info:pmid/21746896#abs:9, info:pmid/20851479#abs:10, info:pmid/24602166#abs:2, info:pmid/23868062#abs:6, info:pmid/21693675#cont:193, info:pmid/16179407#body:242, info:pmid/16352687#body:243, info:pmid/21924257#body:165, info:pmid/21966443#cont:133, info:doi/10.1016/j. ijcard.2009.09.262#body:12 <more data available...>	2	13	achilles tendon {Organ urn: agi-ncimorgan: C0001074}, female reproductive system {Organ urn: agi-ncimorgan: C0700038}, Kidney {Organ urn: agi-ncimorgan: C1278978}

TGFBR3 ---| epithelial to mesenchymal transition	Regulation	The TGF-beta superfamily coreceptor, the type III TGF-beta receptor (TbetaRIII or betaglycan), is required for Epithelial to mesenchymal transition during embryonic heart development and palate fusion., Beta-glycan is also required for the epithelial-mesenchymal transition of cardiac endothelial cells which leads to heart valve formation., Further, misexpression of TBRIII in the ventricle and addition of TGFß2 results in epithelial-mesenchymal transformation in non-transforming ventricular endocardial cells ( ). <more data available...>	info:pmid/17999987#abs:3, info:pmid/14672946#body:78, info:pmid/15766759#body:163, info:pmid/21550381#body:195, info:pmid/22521265#body:195, info:pmid/20299672#body:183	2	6	Mesenchyme {Organ urn: agi-ncimorgan: C0162415}, Heart {Organ urn: agi-ncimorgan: C1281570}, Heart Ventricle {Organ urn: agi-ncimorgan: C0018827}, Epicardium {Organ urn: agi-ncimorgan: C0225968}
CLDN7 ---| cell migration	Regulation	Taken together, our study demonstrates that claudin-7 inhibits cell migration and invasion through ERK/MAPK signaling pathway in response to growth factor stimulation in human lung cancer cells., Furthermore, claudin-6, 7, and 9 enhance gastric adenocarcinoma cell migration and invasion., Our microarray analysis provides several possible mechanisms by which claudin-7 can affect cell migration and invasion, and these different mechanisms are currently being investigated., Claudin-7 overexpression inhibits cell migration and invasion in NCI-H1299, a human non-small cell lung cancer cell line that lacks endogenous claudin-7 expression.	info:pmid/21641901#abs:11, info:pmid/23391410#body:140, info:pmid/21789222#cont:200, info:pmid/22546605#body:22	2	4	Foot {Organ urn: agi-ncimorgan: C2348704}
WNT4 ---| cell migration	Regulation	Lack of Wnt4 gives rise to masculinization of the XX gonad and we showed previously that the role of WNT4 was to inhibit endothelial and steroidogenic cell migration into the developing ovary., We show that WNT4 represses mesonephric endothelial and steroidogenic cell migration in the XX gonad, preventing the formation of a male-specific coelomic blood vessel and the production of steroids., Finally, some cells that are positive for Cyp21, which is normally expressed only in the adrenal gland, are found in the gonads of Wnt-4-deficient embryos, indicating that Wnt-4 may play a role in cell migration or in the sorting of adrenal and gonadal cells during early development. <more data available...>	info:pmid/15581876#abs:3, info:pmid/12835383#abs:7, info:pmid/12399432#abs:5, info:pmid/15620545#body:161, info:pmid/19419993#body:206, info:pmid/21115154#body:52, info:pmid/23027131#cont:430, info:pmid/22682531#cont:14, info:pmid/21397195#body:34, info:pmid/15589122#body:9 <more data available...>	2	14	Gonadal {Organ urn: agi-ncimorgan: C0018067}, Mesonephric {Organ urn: agi-ncimorgan: C0025492}, Adrenal Glands {Organ urn: agi-ncimorgan: C0001625}, Vascular system {Organ urn: agi-ncimorgan: C0489903}, Testis {Organ urn: agi-ncimorgan: C1278981}, Blood Vessels {Organ urn: agi-ncimorgan: C0005847}
FLT1 ---> cell invasion	Regulation	Cancer-associated fibroblast-induced tumor cell invasion was abrogated by VEGF neutralization or Flt1 knockdown in tumor cells., Given our previous finding of VEGFR-1-mediated tumor cell invasion and migration in pancreatic carcinoma cells, we hypothesize that VEGFR-1 plays a role in tumor progression in pancreatic cancer through the induction of epithelial to mesenchymal transition., Furthermore, we found that VEGFR-1 knockdown inhibits tumor cell invasion and metastasis., A novel intracellular isoform of VEGFR-1 activates Src and promotes cell invasion in MDA-MB-231 breast cancer cells. <more data available...>	info:pmid/21115742#abs:7, info:pmid/16397214#abs:9, info:pmid/21989163#cont:98, info:pmid/20512933#title:1, info:pmid/17145519#body:114, info:pmid/23341879#cont:38, info:pmid/23276825#body:421, info:pmid/23384563#body:179, info:pmid/18508995#body:285, info:pmid/20407463#body:240 <more data available...>	2	12	Mesenchyme {Organ urn: agi-ncimorgan: C0162415}, perirenal fascia {Organ urn: agi-ncimorgan: C0227637}, Epicardium {Organ urn: agi-ncimorgan: C0225968}, Blood Vessels {Organ urn: agi-ncimorgan: C0005847}

CCRL2 --+> cell migration	Regulation	Taken together, these results suggest for the first time that elevated CCRL2 in glioma promotes cell migration and invasion., Accordingly, most, but not all studies, describe a lack of CCRL2-mediated Ca-flux and cell migration., Thus, because CCR2 is the only known high-affinity receptor for MCP-1, the chemokine receptor2-MCP-1 axis may influence both phases of Langerhans cell migration., In addition to being cleared by CCX-CKR, CCL19 is also scavenged by another chemokine decoy receptor, CRAM-B, which is expressed on B cells in a maturation stage-dependent manner, and modulates T- and B-lymphocytes and dendritic cell trafficking [ ]. <more data available...>	info:pmid/23142225#abs:7, info:pmid/21272574#body:227, info:pmid/10899907#body:353, info:pmid/24063739#body:26, info:doi/10.1016/j. ddtec.2012.03.005#body:26	2	5	
LCN2 --+> epithelial to mesenchymal transition	Regulation	The present study aimed at determining the effect of Betulinic acid on NGAL-induced epithelial-to-mesenchymal transition., Estrogen receptor alpha may participate in the pathway that leads to Lcn2-induced epithelial to mesenchymal transition., Lcn2 knockdown also indicated that LCN2 promotes epithelial-mesenchymal transition and migration of endometrial epithelial cells., Overexpression of ERalpha in Lcn2-expressing cells reversed the epithelial to mesenchymal transition and reduced Slug expression, suggesting that ERalpha negatively regulates Lcn2-induced epithelial to mesenchymal transition. <more data available...>	info:pmid/23399635#abs:3, info:pmid/19571677#abs:4, info:pmid/24194573#abs:8, info:pmid/19237579#abs:7, info:pmid/23696034#abs:5, info:pmid/22075378#body:85, info:pmid/21874256#cont:26, info:pmid/23300839#cont:37, info:pmid/22559235#cont:8, info:pmid/22982376#cont:137 <more data available...>	2	18	Endometrium {Organ urn: agi-ncimorgan: C1550633}, Mesenchyme {Organ urn: agi-ncimorgan: C0162415}, Breast {Organ urn: agi-ncimorgan: C0006141}, Colon {Organ urn: agi-ncimorgan: C1281569}, Nephrons {Organ urn: agi-ncimorgan: C0027713}
LCN2 --+> cell migration	Regulation	The cell migration-promoting activity of LCN2 in the CNS was verified *in vivo* using mouse models., In contrast, Lcn2 silencing in aggressive breast cancer cells inhibited cell migration and the mesenchymal phenotype., We conclude that Lcn-2 triggered cytokine secretions to prevent RL95-2 cells from undergoing apoptosis and subsequently increased cell migration., LCN2 protein induced upregulation of glial fibrillary acidic protein (GFAP), cell migration, and morphological changes similar to characteristic phenotypic changes termed reactive astrocytosis. <more data available...>	info:pmid/22030398#abs:6, info:pmid/19237579#abs:5, info:pmid/21278918#abs:9, info:pmid/19129400#abs:8, info:pmid/22346773#abs:11, info:pmid/23539193#abs:8, info:pmid/16952550#abs:9, info:pmid/15637066#body:190, info:pmid/19732769#body:174, info:pmid/24194573#cont:409 <more data available...>	2	18	Mesenchyme {Organ urn: agi-ncimorgan: C0162415}, Central Nervous System {Organ urn: agi-ncimorgan: C0927232}, Endometrium {Organ urn: agi-ncimorgan: C1550633}, Liver {Organ urn: agi-ncimorgan: CL384198}, Brain {Organ urn: agi-ncimorgan: C1269537}, Breast {Organ urn: agi-ncimorgan: C0006141}
LCN2 ---> cell invasion	Regulation	We show that the LCN2 protein is required downstream of NFAT1 to increase breast cancer cell invasion., Previously, we reported that lipocalin 2 could revert 4T1-ras-transformed mesenchymal tumor cells to a more epithelial phenotype, increase E-cadherin expression, and suppress cell invasiveness *in vitro* and *in vivo*, indicating that lipocalin 2 is a metastasis suppressor., These data suggest that for KM12C colon carcinoma cells, NGAL overexpression promoted the cell invasion ability through an MMP9-independent mechanism. <more data available...>	info:pmid/22767506#abs:4, info:pmid/17114340#abs:2, info:pmid/19308044#body:238, info:pmid/22800509#body:14, info:pmid/19237579#body:206, info:pmid/21741997#body:149, info:pmid/22728279#body:109, info:pmid/22328933#cont:250, info:pmid/21132267#cont:206, info:pmid/19419554#body:148 <more data available...>	2	11	Ureteric bud {Organ urn: agi-ncimorgan: C1284058}, Spleen {Organ urn: agi-ncimorgan: C1278932}

IGF2BP1 --+> cell migration	Regulation	IGF2BP1 thus promotes the velocity and persistence of tumor cell migration by controlling the expression of signaling proteins., Accordingly, IGF2BP1 knockdown causes MET-like (mesenchymal-epithelial-transition) morphological changes, enhances the formation of cell-cell contacts and reduces cell migration in various mesenchymal-like tumor-derived cells., IGF2BP1 promotes cell migration by regulating MK5 and PTEN signaling., Taken together, these data suggests that IMP1 might regulate cell migration and invasion in JAR cells through other downstream effectors. <more data available...>	info:pmid/22279049#abs:9, info:pmid/23677615#abs:7, info:pmid/22983196#cont:25, info:pmid/23911878#body:109, info:pmid/22266909#cont:48, info:pmid/22800433#cont:410, info:pmid/19726068#body:15	2	7	Mesenchyme {Organ urn: agi-ncimorgan: C0162415}
SOD3 ---| apoptosis	Regulation	EC-SOD is critical for normal cardiac morphology and protects the heart from oxidant-induced fibrosis, apoptosis, and loss of function., In addition, we showed that the thin epidermis of EC-SOD transgenic mice results from the apoptosis of epidermal cells., Extracellular superoxide dismutase (SOD3), which dismutates superoxide anion to hydrogen peroxide, has been shown to reduce the free radical stress derived apoptosis in tissue injuries., Clinical chemistry and tissue pathology analyses showed that adenoviral EC-SOD gene transfer significantly attenuated release of liver enzymes and inhibited necrosis and apoptosis caused by paracetamol overdose. <more data available...>	info:pmid/19695260#abs:12, info:pmid/22251572#abs:8, info:pmid/21909393#abs:1, info:pmid/11529661#abs:7, info:pmid/21804221#abs:10, info:pmid/22140445#cont:277, info:pmid/16962671#body:55, info:pmid/17601801#body:193, info:doi/10.1016/j. jmig.2010.08.256#body:15	2	9	Heart {Organ urn: agi-ncimorgan: C1281570}, Epidermis {Organ urn: agi-ncimorgan: C0014520}, Pulmonary artery {Organ urn: agi-ncimorgan: C0034052}
HIF1A --+> FLT1	PromoterBinding	Conversely, HIF-1alpha knockdown using antisense oligonucleotides, decreased sFlt-1 expression., In contrast, HIF-1a siRNA inhibited the LPS-induced increase in sFlt-1 production (Fig. 4C)., The HIF (hypoxia-inducible factor)-1a transcription factor regulates VEGF and Flt-1 gene transcription [25]., The mRNA levels of VEGF, VEGFR1, Ang-2, and Ang-4 in HPAECs were also increased by either hypoxia or infection with Ad2/HIF-1alpha/VP16 (Figure 3)., Further, Okuyama et al. stated that VEGFR1 expression was upregulated by HIF-1a after culturing Mesenchymal stem cells in hypoxic conditions [48]. <more data available...>	info:pmid/16627691#abs:7, info:pmid/21447646#cont:208, info:pmid/21929511#cont:39, info:pmid/12958144#body:98, info:pmid/20345248#cont:402, info:pmid/15896847#body:281, info:pmid/16574650#body:274, info:pmid/23034156#cont:129, info:pmid/23633414#cont:35	2	9	Villus {Organ urn: agi-ncimorgan: C1519988}, Placenta {Organ urn: agi-ncimorgan: C1550656}
HIF1A --+> FLT1	MolTransport	Furthermore, knockdown of hypoxia-inducible factor-1a inhibited the hypoxia-induced secretion of sFlt-1 and soluble endoglin., Additionally, siRNA-mediated knockdown of HIF-1a inhibited the hypoxia-induced secretion of sFlt-1 and soluble Endoglin., These results indicate that hypoxia-inducible factor-1a is a key mediator of the hypoxia-induced secretion of sFlt-1 and soluble endoglin.	info:pmid/21641407#abs:7, info:doi/10.1016/j. placenta.2010.08.006#body:1429, info:doi/10.1016/j. ajpath.2011.02.013#body:91	2	3	Chorionic villi {Organ urn: agi-ncimorgan: C0008508}

<table>
<tr><td></td><td></td><td></td><td></td><td></td><td></td><td></td></tr>
<tr><td>CRABP2 ---> apoptosis</td><td>Regulation</td><td>Simvastatin interacts with the Retinoic acid system, inducing the expression of the key protein regulating the uptake of retinol (STRA6) and the expression of apoptosis-promoting CRABP2., Inhibition of CRABP-II enhanced retinoic acid-induced apoptosis and sensitized retinoic acid-resistant hepatocellular carcinoma cells to retinoic acid cytotoxicity by attenuating p42/44 MAPK and Akt activation., CRABP2 knockdown influences apoptosis and proliferation in endometrial stromal cells, Retinoic acid and Crabp2 may be regulating apoptosis (Donato and Noy, 2005), whereas the excess retinol is diverted to storage. <more data available...></td><td>info:pmid/23337719#abs:11, info:pmid/20350780#abs:5, info:pmid/20702525#cont:174, info:pmid/17363914#body:213, info:pmid/21515062#body:94, info:pmid/19181377#body:12, info:pmid/17512406#body:91, info:pmid/23874790#cont:23, info:doi/10.1016/j.fertnstert.2012.07.238#body:15</td><td>2</td><td>9</td><td>Endometrium {Organ urn: agi-ncimorgan: C1550633}, Epidermis {Organ urn: agi-ncimorgan: C0014520}</td></tr>
<tr><td>TGFBR3 ---| cell migration</td><td>Regulation</td><td>In particular, betaglycan may be essential for inhibin-mediated regulation of granulosa cell migration and adhesion, which are integrally involved in the ..., ... betaglycan tonically suppresses nuclear factor-?B-mediated breast cancer cell migration via its intracellular interactions with the scaffolding protein ß- ..., Moreover, overexpression of betaglycan resulted in a significant reduction in cell migration whereas inhibin-· gene silencing enhanced both migration and invasion (63)., For instance, Nov, Rgs4 and Tgfbr3, which are inhibitors of cancer cell migration and invasion,48–50 were expressed in increased levels in DA.ACI(Cia10). CONTEXT {10000151,10000158} <more data available...></td><td>info:pmid/19164448#body:229, info:pmid/23322721#cont:654, info:pmid/21174065#cont:280, info:pmid/22048456#cont:376, info:pmid/21550381#body:202, info:pmid/22521265#body:202, info:pmid/23925295#cont:35, info:pmid/20005735#body:84, info:pmid/20208565#body:41, info:pmid/19854227#body:217</td><td>2</td><td>10</td><td>Growing Follicle {Organ urn: agi-ncimorgan: C1512279}, Ovary {Organ urn: agi-ncimorgan: CL384202}, Fingers {Organ urn: agi-ncimorgan: C0016129}</td></tr>
<tr><td>WNT4 ---| apoptosis</td><td>Regulation</td><td>WNT4 may also act to prevent oocyte apoptosis in the developing ovary [35]., Restoring Wnt4 or Wnt5a expression by gene transfection or recombinant protein reduced high glucose-induced cell apoptosis., Wnt4 was previously identified to control the nuclear localization of FOXO1, a transcription factor and known promoter of apoptosis (reviewed in)., Since cell survival during decidualization appears to be regulated by WNT4, we wanted to determine the mechanism by which WNT4 regulates apoptosis., Together with a significant reduction in Spermatogonial stem cell numbers in 5-week-old mutant mouse testes (Fig. 1), this result suggests that WNT4 may also induce Spermatogonial stem cell apoptosis in vivo. <more data available...></td><td>info:pmid/21297984#cont:116, info:pmid/16943306#body:228, info:pmid/22115959#body:189, info:pmid/21163860#cont:247, info:pmid/22253774#cont:111, info:doi/10.1016/j.jamcollsurg.2006.05.149#body:13</td><td>2</td><td>6</td><td>Ovary {Organ urn: agi-ncimorgan: CL384202}, Decidua {Organ urn: agi-ncimorgan: C0011106}, Testis {Organ urn: agi-ncimorgan: C1278981}</td></tr>
<tr><td>TGFBR3 --+> apoptosis</td><td>Regulation</td><td>However, hypoxia-induced apoptosis in cardiac fibroblasts were almost completely prevented by overexpression of TGFBR3., TGFR-3 promotes important signaling events like growth regulation, migration, apoptosis, and differentiation [1,2]., Inhibition of nuclear factor-?B activity sensitizes granulosa cell tumor cells to TGFß2/betaglycan-mediated apoptosis., Transient overexpression of TGFBR3 induces apoptosis in human nasopharyngeal carcinoma CNE-2Z cells., (2010) TGFBR3, a potential negative regulator of transforming growth factor-b signaling, protects cardiac fibroblasts from hypoxia-induced apoptosis. <more data available...></td><td>info:pmid/21792916#abs:5, info:pmid/23826237#cont:17, info:pmid/23322721#cont:219, info:pmid/23387308#title:1, info:pmid/22028866#cont:473, info:pmid/20208565#body:188, info:pmid/19854227#body:214, info:pmid/22952931#cont:216, info:pmid/21550381#body:184, info:pmid/22521265#body:184 <more data available...></td><td>2</td><td>11</td><td>Heart {Organ urn: agi-ncimorgan: C1281570}, Bone and Bones {Organ urn: agi-ncimorgan: C0262950}, Kidney {Organ urn: agi-ncimorgan: C1278978}, Adrenal Cortex {Organ urn: agi-ncimorgan: C0001613}</td></tr>
</table>

<table>
<tr><th></th><th></th><th></th><th></th><th></th><th></th><th></th></tr>
<tr><td>ESRP1 ---| epithelial to mesenchymal transition</td><td>Regulation</td><td>We further show that ectopically expressing ESRP1 inhibits Snail-induced Epithelial-mesenchymal transition, suggesting that down-regulation of ESRP1 is required for function by Snail in Epithelial-mesenchymal transition., Down-regulation of ESRP1 induces epithelial-mesenchymal transition phenotypes ( )., Furthermore, expression of ESRP1/2 mRNA decreased in a model of epithelial-to-mesenchymal transition., ESRP1 and -2 have been proposed as new master regulators of epithelial-mesenchymal transition (28), and our finding that ESRP1 and E-cadherin mRNA were ... <more data available...></td><td>info:pmid/22961986#abs:6, info:pmid/24035864#body:248, info:pmid/19328061#body:18, info:pmid/22585092#cont:162, info:pmid/21393860#cont:182, info:pmid/23825128#cont:397, info:pmid/22349259#cont:190, info:pmid/22548723#body:153, info:pmid/24331928#body:38</td><td>2</td><td>9</td><td>Mesenchyme {Organ urn: agi-ncimorgan: C0162415}</td></tr>
<tr><td>KRT18 ---> cell invasion</td><td>Regulation</td><td>KRT18 plays an important role in tumor cell invasion by increasing cell flexibility and motility., Moreover, in line with the emergence of keratins as multifunctional regulators of epithelial malignancies, the persistence of K8/K18 IFs is a hallmark of invasive squamous cell carcinoma, where such perturbed K8/K18 expression appears to contribute to cell invasiveness., With regard to cancer, there is accumulating evidence showing, for instance, that persistence of K8/K18 IFs is a hallmark of invasive squamous cell carcinoma, where such perturbed K8/K18 expression appears to contribute to cell invasiveness through an actin-dependent motility [18].</td><td>info:pmid/15177556#body:116, info:pmid/23164509#body:22, info:pmid/22685604#cont:40</td><td>2</td><td>3</td><td>Peritoneal Cavity {Organ urn: agi-ncimorgan: C1704247}</td></tr>
<tr><td>IGF2BP1 ---| apoptosis</td><td>Regulation</td><td>CRD-BP down-regulation contributes to cell sensitization by increasing apoptosis and diminishing melanoma cell growth in response to chemotherapeutic agents., Knockdown of CRD-BP inhibits NF-kappaB activity, induces apoptosis, and suppresses proliferation and tumorigenic properties of melanoma cells., Furthermore, we show that loss of IMP-1 induces Caspase-3- and PARP-mediated apoptosis, and inhibits K-Ras expression in SW480 cells, which is rescued by CYFIP2 knockdown., A study has indicated that CRD-BP, the ortholog of IMP1, promotes cell proliferation by suppressing apoptosis. <more data available...></td><td>info:pmid/21981993#abs:5, info:pmid/18454174#abs:4, info:pmid/21252116#abs:8, info:pmid/23911878#body:11, info:pmid/19647520#body:68</td><td>2</td><td>5</td><td></td></tr>
<tr><td>GLIPR2 --+> epithelial to mesenchymal transition</td><td>Regulation</td><td>However, whether GLIPR-2 could induce type 3 epithelial-to-mesenchymal transition in carcinogenesis needs further investigation., The origin of these myofibroblasts is not known, but in vitro studies have shown that GLIPR-2 can induce epithelial to mesenchymal transition in a renal epithelial cell line., GLIPR-2 overexpression in HK-2 cells promotes an epithelial-to-mesenchymal transition through extracellular signal-regulated kinases 1 and 2 activation.</td><td>info:pmid/24204846#abs:3, info:pmid/17055234#abs:7, info:pmid/23516513#cont:222</td><td>2</td><td>3</td><td>Kidney {Organ urn: agi-ncimorgan: C1278978}, Mesenchyme {Organ urn: agi-ncimorgan: C0162415}</td></tr>
<tr><td>TXNIP --+> epithelial to mesenchymal transition</td><td>Regulation</td><td>These results suggested that knockdown of TXNIP antagonized high glucose-induced Epithelial to mesenchymal transition by inhibiting reactive oxygen species production, activation of p38 MAPK and ERK1/2, and expression of TGF-ß1, highlighting TXNIP as a potential therapy target for diabetic nephropathy., Deficiency of thioredoxin binding protein-2 enhances TGF-ß signaling and promotes epithelial to mesenchymal transition.</td><td>info:pmid/24041652#abs:11, info:pmid/22768160#title:1</td><td>2</td><td>2</td><td></td></tr>
</table>

THBS1 ---> epithelial to mesenchymal transition	Regulation	Using two Pofut2 gene-trap lines, we demonstrated that O-fucosylation of Thrombospondin type 1 repeats was essential for restricting epithelial to mesenchymal transition in the primitive streak, correct patterning of mesoderm, and localization of the definitive endoderm., TGF-ß and thrombospondin-1 from such tumors could be partly responsible for both Treg induction and the further enhancement of Epithelial-mesenchymal transition in an autocrine manner., Progress was made recently by Du et al (2010) using Pofut2 knockout mice where they found that O-fucosylation of thrombospondin type 1 repeats is critical for restricting epithelial-to-mesenchymal transition, correct patterning of the mesoderm, and localization of the endoderm in embryonic development.	info:pmid/20637190#abs:3, info:pmid/19249678#body:45, info:pmid/22588082#cont:32	2	3	Primitive Streak {Organ urn: agi-ncimorgan: C0033153}
SOD3 ---| cell migration	Regulation	Furthermore, Langerhans cell migration to lymph nodes was impaired in EC-SOD transgenic mice., In addition, we demonstrated that SOD3 inhibits ovalbumin-induced airway extracellular remodeling and T helper 2 cell trafficking., Figure 6 Ddx5 and Ddx17–regulated SOD3 expression is involved in cell migration and invasion., This result is consistent with a previous report that showed that EC-SOD reduces inflammatory cell migration (Laurila et al., 2009)., In addition, we demonstrated that SOD3 inhibits OVA-induced airway extracellular remodeling and Th2 cell trafficking.	info:pmid/17392825#abs:7, info:pmid/22583151#abs:7, info:pmid/23022728#cont:425, info:pmid/23223134#cont:661, info:doi/10.1016/j. jdermsci.2012.11.328#body:7	2	5	Lymph Nodes {Organ urn: agi-ncimorgan: C0154054}, airway {Organ urn: agi-ncimorgan: C0458827}
WNT4 ---> epithelial to mesenchymal transition	Regulation	Wnt4 regulates epithelial to mesenchymal transition and ductal branching.	info:pmid/24099911#body:166	2	1	Mammary Gland {Organ urn: agi-ncimorgan: C0929301}
THBS1 --+> MMP2	Expression	MMP2 mRNA was upregulated 1.7-fold by TSP-1 at 10 and 20 microg/mL., As indicated above within this section, different TSP-1 fragments produced after thrombin treatment can stimulate or inhibit MMP-2 and MMP-9 synthesis., Taken together, these observations suggest that TSP1 and -2 can inhibit generation of active gelatinase (MMP2 and MMP9) species., It suggests that SHWSPW heparin-binding sequence within TSRs of TSP-1, and/or an adjacent sequence might be involved in collagen-binding domain123 interaction with TSP-1, further influencing MMP-2 levels. <more data available...>	info:pmid/12844104#abs:11, info:pmid/20006665#body:348, info:pmid/10900205#body:318, info:pmid/18222489#body:78, info:pmid/19129184#body:414, info:pmid/15036264#body:249, info:pmid/12128069#body:75, info:pmid/17126831#body:133, info:pmid/12235282#body:112, info:pmid/17761938#body:175	2	10	Dermis {Organ urn: agi-ncimorgan: C0011646}, Cardiovascular system {Organ urn: agi-ncimorgan: C1269562}, Carotid Arteries {Organ urn: agi-ncimorgan: C0007272}

THBS1 ---> MMP2	DirectRegulation	A binding assay was used to determine the specificity of TSP-1 binding to MMP2., Although MMP2 interacted with TSP1 and TSP2 via its gelatin-binding domain or a closely mapping site, neither TSP1 nor TSP2 was degraded by MMP2 *in vitro*., collagen-binding domain123, as well as individual domains, behave as competitive inhibitors of the TSP-1-directed endocytic clearance of active MMP-2, but not of its latent form, by HT1080 fibrosarcoma cells., Furthermore, showed that TSP-1 may also interact with MMP2 and by this mean inhibit the proMMP2 activation. <more data available...>	info:pmid/12844104#abs:8, info:pmid/10900205#abs:8, info:pmid/18222489#abs:3, info:pmid/18321763#body:120, info:pmid/22798012#cont:143, info:pmid/11805097#body:48, info:pmid/23853587#cont:754, info:pmid/23749112#cont:285, info:pmid/21744247#cont:75, info:pmid/15655118#body:164 <more data available...>	2	41	Aorta {Organ urn: agi-ncimorgan: C1278934}, Blood Vessels {Organ urn: agi-ncimorgan: C0005847}, Arteries {Organ urn: agi-ncimorgan: C0003842}, Heart {Organ urn: agi-ncimorgan: C1281570}, trabecular meshwork {Organ urn:agi-organ:trabecular%20 meshwork}, Coronary artery {Organ urn: agi-ncimorgan: C1269008}, Tendon {Organ urn: agi-ncimorgan: C0039508}, Carotid Arteries {Organ urn: agi-ncimorgan: C0007272}, Basal lamina {Organ urn: agi-ncimorgan: C0085872}
KRT18 ---> KRT14	Regulation	In this report, we identified K18 phospho-Ser33 as the site that regulates keratin/14-3-3 binding during mitosis, and showed that K18 Ser33 phosphorylation generates a unique motif that is essential but not sufficient for binding of 14-3-3 proteins.	info:pmid/9524113#body:52	2	1	
KRT14 ---> KRT18	Regulation	Similarly, ITGA6, ITGB4, and CK14 decreased (Figure 1d) and CK18 (Figure 1e) increased at the protein level., When the urea concentration was raised to 8 M under otherwise identical conditions, the K5-K18 tetramers were destabilized to a greater extent than K5-K14 ones (data not shown).	info:pmid/20379195#cont:82, info:pmid/9786957#body:338	2	2	Breast {Organ urn: agi-ncimorgan: C0006141}
LCN2 ---- MMP2	Binding	Neutrophil gelatinase-associated lipocalcin (NGAL) is an early indicator of kidney injury., Levels of Cystatin-C, as well as serum and urine neutrophil gelatinase-associated lipocain (NGAL), were significantly augmented., This review discusses a member of the lipocalin superfamily, 24p3 or neutrophil gelatinase-associated lipocalcin (NGAL), which induces the formation of kidney epithelia., In this study, levels of interleukin -8, IL-6, interleukin-1 receptor antagonist (IL-1ra), secretory leukocyte protease inhibitor (SLPI), and neutrophil gelatinase-associated lipocalcin (NGAL) were measured in divers before and after a 2-mo period of daily diving. <more data available...>	info:pmid/16773412#abs:2, info:pmid/22725830#abs:7, info:pmid/12788784#abs:4, info:pmid/12015350#abs:3, info:pmid/23535405#abs:2, info:pmid/23477150#abs:1, info:pmid/21714857#abs:4, info:pmid/21311195#abs:1, info:pmid/22268365#abs:2, info:pmid/11574282#body:50 <more data available...>	2	21	Kidney {Organ urn: agi-ncimorgan: C1278978}, Heart {Organ urn: agi-ncimorgan: C1281570}, Renal Tubule {Organ urn: agi-ncimorgan: CL355448}, Ovary {Organ urn: agi-ncimorgan: CL384202}
CASP4 ---> VIM	ProtModification	Interestingly, caspase-4 led to a dose-dependent cleavage of human vimentin ( ) that was blocked by a specific caspase-4 inhibitor (, lanes 5 and 6).	info:pmid/16127151#body:188	2	1	Synovial Membrane {Organ urn: agi-ncimorgan: C0039099}

ST14 --+> MMP3	Regulation	Moreover, it was found that purified matriptase activated one of the important matrix metalloproteinases, stromelysin (MMP-3)., This is intriguingly analogous to the phenotype of transgenic mice with overexpression of epithin in the skin, and epithin can proteolytically activate MMP-3., Epithin/ matriptase has been shown to directly activate the latent forms of HGF ( ), uPA ( ) and MMP3 ( ) *in vitro*. uPA mRNA is expressed in mammary epithelium of nulliparous adult, early and mid-pregnancy, is subsequently down regulated during lactation, and reappears during involution ( ). <more data available...>	info:pmid/16999819#abs:7, info:pmid/20398629#body:109, info:pmid/19853659#body:72, info:doi/10.2353/ ajpath.2010.100228#body:215	2	4	Skin {Organ urn: agi-ncimorgan: C0684084}, Prostate {Organ urn: agi-ncimorgan: C1278980}
EGLN3 ---> HIF1A	ProtModification	Many studies have shown that PHD3 not only can hydroxylate Hypoxia inducible factor-1a, but also has various other biological functions., The present study identifies a novel layer in the regulation of HIF1a hydroxylation by PHD1/3., Recent report, however, indicates that PHD2 and 3 hydroxylate HIF-1a and HIF-2a with similar efficiency., In 2007, Fedulova and co-workers demonstrated that PHD-3 hydroxylates P567 in HIF-1a, which is not LxxLAP motif compliant., Although PHD1 and PHD3 can hydroxylate HIF-1a *in vitro*, HIF does not appear to be their physiological target in the cell (32). <more data available...>	info:pmid/24049515#abs:3, info:pmid/15210114#body:113, info:pmid/22804960#cont:14, info:pmid/23886708#body:22, info:pmid/23807085#cont:134, info:pmid/21575608#body:75, info:pmid/19683567#body:12, info:pmid/21665470#body:6, info:pmid/21421125#body:102, info:pmid/24222137#cont:183 <more data available...>	2	41	Retina {Organ urn: agi-ncimorgan: C1962966}, Fingers {Organ urn: agi-ncimorgan: C0016129}, Kidney {Organ urn: agi-ncimorgan: C1278978}, Heart {Organ urn: agi-ncimorgan: C1281570}
HIF1A ---> EGLN3	Expression	Reporter analysis shows that the hypoxic induction of the PHD2 promoter is hypoxia-inducible factor-1a dependent, whereas PHD3 promoter/enhancer activity is dependent on both hypoxia-inducible factor-1a and hypoxia-inducible factor-2a., This is because the transcription of PHD3 is regulated by hypoxia inducible factor-1a., They also demonstrated a massive transcription induction of PHD-3 mRNA promoted by HIF-1a., Although hypoxia decreases overall PHD activity, upregulation of HIF-1a induces the expression of PHD2 and PHD3.70 <more data available...>	info:pmid/22451659#abs:5, info:pmid/21575608#body:90, info:pmid/23886708#body:130, info:pmid/23486514#cont:106, info:pmid/20980436#cont:139, info:pmid/17003045#body:219, info:pmid/24015760#cont:171, info:pmid/17434750#body:223, info:pmid/21927022#cont:66, info:pmid/17387348#body:118 <more data available...>	2	13	nucleus pulposus {Organ urn: agi-ncimorgan: C1185714}
EGLN3 ---> HIF1A	Expression	Prolyl hydroxylase-3 (PHD3) regulates degradation of hypoxia-inducible factor-1a., Overexpression of SM-20/ PHD3 transiently resulted in a decrease in hypoxia-inducible factor-1 alpha protein concentrations under basal conditions as well as after stimulation with ANG II., We recently identified a new WD-repeat protein designated Morg1 (MAPK organizer 1) that interacts with prolyl hydroxylase 3 (PHD3), an important enzyme involved in the regulation of HIF-1a and HIF-2a expression., We recently identified a novel WD-repeat protein designated Morg1 (MAPK organizer 1) that interacts with prolyl hydroxylase 3 (PHD3), an important enzyme involved in the regulation of HIF-1alpha and HIF-2alpha expression. <more data available...>	info:pmid/20978507#abs:2, info:pmid/15308873#abs:14, info:pmid/22982595#abs:3, info:pmid/19726548#abs:3, info:pmid/19574556#body:166, info:pmid/21818118#cont:272, info:pmid/22138393#body:4, info:pmid/21575608#body:75, info:pmid/12181324#body:281, info:pmid/21620138#body:127 <more data available...>	2	25	Kidney {Organ urn: agi-ncimorgan: C1278978}, Placenta {Organ urn: agi-ncimorgan: C1550656}, Lower Extremity {Organ urn: agi-ncimorgan: C0023216}
MMP3 ---> THBS1	ProtModification	Biosynthesis and expression of a disintegrin-like and metalloproteinase domain with thrombospondin-1 repeats-15: a novel versican-cleaving proteoglycanase.	info:pmid/24220035#title:1	2	1	

THBS1 ---| MMP3	Expression	Moreover, TSP-1 null hearts had increased myocardial matrix metalloproteinase 3 expression and enhanced matrix metalloproteinase 9 activation after pressure overload., Furthermore, matrix metalloproteinase 3 and 9 are upregulated in Tsp1-knockout mice., Thus, processing and consequent activation of pro-MMP-9 to MMP-9 by physiological activators such as MMP-3 is blocked by TSP-1., thrombospondin-1 may inhibit MMP-9 activity by preventing activation of pro-MMP-2, and MMP-3 can cleave and activate MMP-2 and MMP-9.	info:pmid/21947471#abs:10, info:pmid/24492068#body:98, info:doi/10.1016/S1471-4914(01)02075-5#body:17, info:doi/10.1016/j.bbrc.2006.12.064#body:99	2	4	Heart {Organ urn: agi-ncimorgan: C1281570}, Mammary Gland {Organ urn: agi-ncimorgan: C0929301}
CDH1 ---- ST14	Binding	Epithin colocalized with E-cadherin at the membrane contacts of the compacted morula-stage embryo as revealed by double-staining immunocytochemistry and confocal microscopy, respectively., Matriptase is expressed during intestinal epithelial differentiation and colocalizes with E-cadherin to apical junctional complexes in differentiated polarized Caco-2 monolayers., Epithin, a type II membrane serine protease, colocalizes with E-cadherin in mouse conceptus ( )., suppressor of tumorigenicity 14 encodes a membrane-associated serine protease (matriptase) that co-localizes with E-cadherin, can process the hepatocyte growth factor precursor and plays a role in epithelial differentiation and maintenance of tight junctions.	info:pmid/15848395#abs:6, info:pmid/20142489#abs:4, info:pmid/18155829#body:75, info:pmid/20980099#body:197	2	4	Morula {Organ urn: agi-ncimorgan: C0026573}, Intestines {Organ urn: agi-ncimorgan: C0021853}, Conceptus {Organ urn: agi-ncimorgan: C1516779}
KRT18 ---> FAS	Expression	The results also did not support the idea that K8/K18 filaments influence the expression of Fas on the surface of bovine luteal cells., Notably, intermediate filaments such as keratin 8 and 18 have been suggested to prevent CD95 targeting to the membrane by a process involving microtubules., As demonstrated in Fig. 4 B, the total content of the death receptor was not affected by the K8-null mutation, thus suggesting that K8/K18 modulate Fas targeting to the surface membrane., Furthermore, keratin 8/18 filaments regulate Fas receptor signaling, and modest overexpression of the phosphorylation mutant K8 S73A significantly enhances Fas-mediated apoptosis in transgenic mouse livers ( ). <more data available...>	info:pmid/23113883#abs:10, info:pmid/15501679#body:81, info:pmid/11514590#body:121, info:pmid/22960429#body:145, info:pmid/19282868#body:38	2	5	Liver {Organ urn: agi-ncimorgan: CL384198}, Kidney {Organ urn: agi-ncimorgan: C1278978}
FAS ---> KRT18	Regulation	SV40 infection also suppressed CD95-mediated cleavage of cytokeratin-18 (Figure 1b)., Fas administration also modulates Keratin 8/K18 phosphorylation (Ku et al., 2003), including an increase in Keratin 8 serine 73/S431 and a decrease in K18 S33 phosphorylation in all transgenic lines (Fig. 2C)., For instance, Fas stimulation activates the anti-apoptotic ERK1/2 signaling pathway at the early period and the stress-associated kinase JNK at a later period, and this results to phosphorylation of specific Ser residues on K8 and K18.	info:pmid/11803462#body:94, info:pmid/16818723#body:86, info:pmid/17498695#body:79	2	3	
KRT18 ---> VIM	ProtModification	During viral replication, all of vimentin were degraded and phosphorylated keratin 18 increased.	info:pmid/3396082#abs:5	2	1	

VIM ---> KRT18	DirectRegulation	It was already shown that K18 could possibly interact with vimentin to form mixed filaments., honokiol blocked TGFß/TNFa-induced modulation of mesenchymal and epithelial markers leading to decreased expression of vimentin and fibronectin and elevated expression of occludin and CK-18 (B, C)., Furthermore, green fluorescent protein-K18 colocalized exclusively with the endogenous tonofibrils and not with the endogenous vimentin fibrils as demonstrated by the direct observation of a live PtK2 cell (Fig 1G), followed by fixing and processing the same cell for double immunofluorescence (Fig 1H and Fig I). <more data available...>	info:pmid/20109457#body:97, info:pmid/24508063#body:98, info:pmid/11331302#body:199, info:pmid/22906492#body:102	2	4	Mesenchyme {Organ urn: agi-ncimorgan: C0162415}
CLDN7 ---> CDH1	Expression	Decreased claudin-7 expression, in turn, leads to attenuation of E-cadherin expression., Notably, similar to our findings, claudin-7-dependent regulation of E-cadherin was unidirectional because forced E-cadherin expression in the same cells did not affect claudin-7 expression., Knockdown of claudin-7 expression in esophageal squamous cell carcinoma cells induces loss of E-cadherin, along with increased cell growth and enhanced cell invasion [84]., While the mislocalization of claudin-7 in esophageal squamous cell carcinoma leads to the loss of E-cadherin expression, N-glycosylation of E-cadherin has been shown to stabilize tight junctions [8]. <more data available...>	info:pmid/23969095#body:6, info:pmid/21878201#body:134, info:pmid/24009024#cont:393, info:pmid/20953359#cont:22, info:pmid/20334898#body:105, info:pmid/17255337#body:234	2	6	Ovary {Organ urn: agi-ncimorgan: CL384202}, Breast {Organ urn: agi-ncimorgan: C0006141}, Esophagus {Organ urn: agi-ncimorgan: C1278919}
SOD3 ---> HIF1A	Expression	Combined, these results suggest that the Hypoxia-inducible factor-1a suppression by Extracellular superoxide dismutase may be due to the decreased levels of O2- and not due to increased levels of H2O2 or other hydroperoxides., High expression levels of SOD3 was reported to significantly induce the expression of HIF-1a in tumors, under hypoxic conditions, and thus demonstrates a relationship between SOD3 and HIF-1a pathways., Conclusion: Inhibition of NADPH oxidoreductase attenuates expression of SOD3, which decreases expression of hypoxia inducible factor-1a, and subsequently promotes apoptosis.	info:pmid/24509158#body:108, info:pmid/21620448#body:115, info:doi/10.1016/j.jmig.2010.08.256#body:15	2	3	
CDH1 ---> KRT18	ProtModification	Confocal Z-stacks of cultured trophoblasts co-stained for E-cadherin (green), cleaved cytokeratin 18 (red) and DNA (blue)., We assessed the localization patterns of caspase-cleaved forms of cytokeratin 18 by co-staining tissues for E-cadherin, DNA and caspase-cleaved forms of cytokeratin 18 and examination by Z-stack confocal microscopy.	info:pmid/23102999#body:49, info:pmid/22341340#body:49	2	2	Villus {Organ urn: agi-ncimorgan: C1519988}
KRT18 --+> CDH1	Regulation	Both CK18 and E-cadherin (Cdh1) gene expression increased significantly from day 0 to day 7 (10.3-fold for CK18 and 1.7-fold for E-cadherin) and from day 7 to day 21 (1.6-fold for CK18 and 3.2-fold for E-cadherin) while vimentin and Snail1 except Twist1 demonstrated downward trend in expression between day 0 and day 21 (1.9-fold for vimentin and 4.5-fold for Snail1) (Figure 5B).	info:pmid/21347296#body:203	2	1	

<table>
<tr><td>TXNIP ---| HIF1A</td><td>Expression</td><td>Taken together, these findings indicate that VDUP1 is a novel tumor suppressor which mediates the nuclear export of pVHL/HIF1alpha complex to destabilize HIF1alpha., TXNIP also associates with the von Hippel-Landau protein (pVHL), enhancing the interaction between pVHL and HIF1a to promote nuclear export and degradation of HIF1a., TXNIP may affect the glucose uptake through the suppression of two important regulators of glycolysis, Akt [56] and HIF-1a [80]., ... the a-ar-restin gene family, TXNIP also inhibits glucose uptake and lactate production by destabilizing the hypoxia-induced transcription factor (HIF1a) ... <more data available...></td><td>info:pmid/18062927#abs:6, info:pmid/20584310#body:82, info:pmid/20844768#cont:296, info:pmid/22904171#cont:139, info:pmid/24098117#cont:112</td><td>2</td><td>5</td><td>Ovarian Follicle {Organ urn: agi-ncimorgan: C1283799}, Lung {Organ urn: agi-ncimorgan: C1278908}</td></tr>
<tr><td>TXNIP ---> HIF1A</td><td>MolTransport</td><td>VDUP1 mediates nuclear export of HIF1alpha via CRM1-dependent pathway., (2008) VDUP1 mediates nuclear export of HIF1alpha via CRM1-dependent pathway.</td><td>info:pmid/18062927#title:1, info:pmid/20844768#cont:591</td><td>2</td><td>2</td><td></td></tr>
<tr><td>FAS ---> THBS1</td><td>Regulation</td><td>The endothelial cell apoptotic activity of THBS-1 is mediated through interaction with the Fas/Fas ligand, caspase-3 activation and FAK fragmentation (Volpert et al, 2002).</td><td>info:pmid/19738618#body:280</td><td>2</td><td>1</td><td></td></tr>
<tr><td>THBS1 --+> FAS</td><td>Regulation</td><td>Specificity for remodeling vessels is provided by the dependence of TSP1-induced apoptosis on Fas-Fas ligand interactions, which occur preferentially in capillary sprouts.</td><td>info:pmid/12231357#body:111</td><td>2</td><td>1</td><td>Cornea {Organ urn: agi-ncimorgan: C1550625}</td></tr>
<tr><td>ITGB3 ---- THBS1</td><td>Binding</td><td>Because TSP-1 is an integrin ß3 ligand, we sought to determine whether upregulation of TSP-1 might promote the association of aVß3 with PKCß and trigger downstream signaling.</td><td>info:pmid/20184965#body:132</td><td>2</td><td>1</td><td></td></tr>
<tr><td>THBS1 --+> ITGB3</td><td>Regulation</td><td>In aortas from diabetic animals and in hyperglycemic cells, the absence of TSP-1 reduced the association of integrin ß3 and PKCß (D and E) Moreover, under conditions of euglycemia, the association of PKCß with integrin ß3 was promoted by the addition of TSP-1 to smooth muscle cells (F).</td><td>info:pmid/20184965#body:139</td><td>2</td><td>1</td><td>Aorta {Organ urn: agi-ncimorgan: C1278934}</td></tr>
<tr><td>LCN2 --+> CDH1</td><td>Expression</td><td>Overexpression of lipocalin-2 correlated with a lower extent of epithelial-to-mesenchymal transition ($p < 0.05$), increased E-cadherin expression ($p < 0.05$), decreased vimentin expression ($p < 0.05$), and reduced cancer cell migration and invasion in pancreatic cancer., We found that lipocalin 2 could also convert 4T1-Ras-transformed mesenchymal tumor cells to an epithelial phenotype, increase E-cadherin expression, and suppress cell invasiveness in vitro and tumor growth and lung metastases in vivo., Previously, we reported that lipocalin 2 could revert 4T1-ras-transformed mesenchymal tumor cells to a more epithelial phenotype, increase E-cadherin expression, and suppress cell invasiveness in vitro and in vivo, indicating that lipocalin 2 is a metastasis suppressor. <more data available...></td><td>info:pmid/23539193#abs:8, info:pmid/15691834#abs:2, info:pmid/17114340#abs:2, info:pmid/19237579#body:97, info:pmid/21132267#cont:263, info:pmid/18768801#body:208, info:pmid/22075378#body:50, info:pmid/22328933#cont:250, info:pmid/17604154#body:86</td><td>2</td><td>9</td><td>Mesenchyme {Organ urn: agi-ncimorgan: C0162415}, Basal lamina {Organ urn: agi-ncimorgan: C0085872}</td></tr>
</table>

HIF1A ---| THBS1	Expression	When the cells were cultured under hypoxic conditions, mRNA and protein levels of TSP-1, and mRNA levels of integrin beta(3) were increased with the increase in HIF-1alpha protein., Present studies with human umbilical vein endothelial cells suggest that MCPIP-induced angiogenesis is mediated via hypoxia-inducible factor (HIF-1a), vascular endothelial growth factor (VEGF), and silent information regulator (SIRT-1) induction that results in the inhibition of angiogenesis inhibitor thrombospondin-1., HIF1-a has been shown to induce pro-angiogenic programs through modulation of a variety of molecules including but not limited to VEGF, FLT1, ANGPT2, THBS1 and CYR61 [29–31,33,37,38]. <more data available...>	info:pmid/18384112#abs:4, info:pmid/24048733#abs:3, info:pmid/23922772#cont:347, info:pmid/24412200#body:235, info:pmid/23164805#body:187	2	5	Coronary artery {Organ urn: agi-ncimorgan: C1269008}, Ground substance {Organ urn: agi-ncimorgan: C1383501}
CLDN7 ---| MMP3	Expression	Deletion of claudin-7 also markedly up-regulated the expression levels of MMP-3 and MMP-7, which could cause intestinal tissue damage and inflammation.	info:pmid/22044670#body:145	2	1	Intestines {Organ urn: agi-ncimorgan: C0021853}
MMP3 ---> CLDN7	Expression	Intestines of Cldn7 (-/-) mice produced significantly higher levels of cytokines, the nuclear factor ?B p65 subunit, and cyclooxygenase 2; they also up-regulated expression of matrix metalloproteinases -3 and -7. small interfering RNA in epithelial cell lines showed that the increased expression of MMP-3 resulted directly from claudin-7 depletion, whereas that of MMP-7 resulted from inflammation.	info:pmid/22044670#abs:8	2	1	Intestines {Organ urn: agi-ncimorgan: C0021853}
GLIPR2 ---| CDH1	Expression	The epithelial-to-mesenchymal transition PCR Array analysis indicated that GLIPR-2 overexpression in HK-2 cells increased E-cadherin expression but decreased vimentin expression., An *in vitro* assay suggests that GLIPR-2 can promote epithelial cells to become more like fibroblasts, both by changes in morphology and decreased expression of E-cadherin.	info:pmid/23516513#cont:280, info:pmid/17055234#body:97	2	2	Mesenchyme {Organ urn: agi-ncimorgan: C0162415}
GLIPR2 ---> VIM	Expression	Expression of vimentin did not appear to be significantly increased in the presence of GLIPR-2 conditioned medium., The epithelial-to-mesenchymal transition PCR Array analysis indicated that GLIPR-2 overexpression in HK-2 cells increased E-cadherin expression but decreased vimentin expression.	info:pmid/17055234#body:60, info:pmid/23516513#cont:280	2	2	Mesenchyme {Organ urn: agi-ncimorgan: C0162415}
LCN2 ---> HIF1A	Expression	The regulation of HIF-1a and VEGF by Lcn2 was also demonstrated in the aggressive MDA-MB-231 cell line., Furthermore, LCN2 promotes expression of VEGF and HIF1A which contribute to enhanced vascularity., However, on the contrary, some studies have demonstrated that NGAL can inhibit the pro-neoplastic factor HIF-1alpha, FA-Kinase phosphorylation and also VEGF synthesis, thus suggesting that, in alternative conditions, NGAL also, paradoxically, has an anti-tumoral and anti-metastatic effect in neoplasias of, for example, the colon, ovary and pancreas. <more data available...>	info:pmid/22982376#abs:7, info:pmid/23056397#abs:8, info:pmid/19540040#abs:5, info:pmid/23022179#body:292	2	4	Colon {Organ urn: agi-ncimorgan: C1281569}, Breast {Organ urn: agi-ncimorgan: C0006141}

HIF1A ---> LCN2	Expression	(D) Hif-1a silencing partially overcomes EGFR-induced Lcn2 upregulation., epidermal growth factor-induced Lcn2 expression was found to be mediated through stabilization of the transcription factor hypoxia inducible factor-1 alpha (HIF-1a).	info:pmid/20921623#cont:245, info:pmid/22513004#body:614, info:doi/10.1016/j.bbcan.2012.03.008#body:614	2	3	Kidney {Organ urn: agi-ncimorgan: C1278978}, Nephrons {Organ urn: agi-ncimorgan: C0027713}
LCN2 ---> VIM	Expression	Incubation of Human peritoneal mesothelial cell with recombinant NGAL reversed the transforming growth factor-beta-induced up-regulation of Snail and vimentin but rescued the down-regulation of E-cadherin., Overexpression of lipocalin-2 correlated with a lower extent of epithelial-to-mesenchymal transition ($p < 0.05$), increased E-cadherin expression ($p < 0.05$), decreased vimentin expression ($p < 0.05$), and reduced cancer cell migration and invasion in pancreatic cancer., To investigate the function of Lcn2 in breast cancer progression, Lcn2 was overexpressed in human breast cancer cells and was found to up-regulate mesenchymal markers, including vimentin and fibronectin, down-regulate the epithelial marker E-cadherin, and significantly increase cell motility and invasiveness. <more data available...>	info:pmid/19148711#abs:7, info:pmid/23539193#abs:8, info:pmid/19237579#abs:3, info:pmid/22075378#body:50	2	4	Peritoneum {Organ urn: agi-ncimorgan: C0496954}, Mesenchyme {Organ urn: agi-ncimorgan: C0162415}, Basal lamina {Organ urn: agi-ncimorgan: C0085872}
FAS ---| PDGFRA	Expression	Expression of PDGFRa was decreased following GANT-61 treatment with concomitant increase in Fas., It is interesting to note that the expression of PDGFRa was decreased following GDC-0449 treatment with concomitant increase in Fas.	info:pmid/23200667#body:135, info:pmid/22087285#cont:117	2	2	
PDGFRA ---> FAS	Regulation	Analysis of the molecular mechanisms of GANT61-induced cytotoxicity in HT29 cells showed increased Fas expression and decreased expression of PDGFRa, which also regulates Fas., Analysis of the molecular mechanisms of GDC-0449-induced cell death in cancer stem cells showed increased Fas expression and decreased expression of PDGFRa, which also regulates Fas.	info:pmid/21135115#abs:7, info:pmid/22087285#cont:128	2	2	
KRT18 --+> epithelial to mesenchymal transition	Regulation	Using protein immunoblotting, we confirmed that MCF7-Six1 lines contained increased levels of the mesenchymal protein fibronectin and decreased levels of the epithelial proteins cytokeratin 18, claudin-1, and claudin-8 (Figure 3B and Supplemental Figure 2), consistent with a Six1-induced epithelial-mesenchymal transition.	info:pmid/19726885#body:133	2	1	Mesenchyme {Organ urn: agi-ncimorgan: C0162415}
TGFBR3 ---> cell invasion	Regulation	Knockdown of TGFBR3 decreases cell invasion through Matrigel., Betaglycan expression decreases granulosa cell invasion through Matrigel., Furthermore, Hyaluronic acid-stimulated epicardial cell invasion required Tgfbr3.112, As we have previously demonstrated, TGFBR3 is frequently downregulated during cancer progression and suppresses tumor cell invasion and metastasis in a cell-autonomous manner (25, 26)., Specifically, four of these genes (Pawr, Per1, Igfbp6 and Thbd) negatively regulate cell proliferation and growth, and three genes (Rgs4, Nov and Tgfbr3) negatively regulate cell invasion (Table 3).	info:pmid/23835618#cont:249, info:pmid/19164448#body:185, info:pmid/22679138#cont:412, info:pmid/23925295#cont:361, info:pmid/22048456#cont:151	2	5	Epicardium {Organ urn: agi-ncimorgan: C0225968}, Synovial Membrane {Organ urn: agi-ncimorgan: C0039099}

<table>
<tr><td>KRT18 ---> cell migration</td><td>Regulation</td><td>Prior studies have shown that Keratin 8 and K18 regulate cell migration in various cell types including OSCC [14,33,34]., Keratin 8 and 18 loss in epithelial cancer cells increases collective cell migration and cisplatin sensitivity through claudin1 up-regulation., We thus sought to assess to which extent the K8/K18 loss affects hepatoma cell migration and whether it occurs through a novel protein kinase C mediation., Taken together these results suggest that K8 and K18 promote the cell migration and neoplastic progression by modulating ?4-integrin-mediated signalling in oral squamous cell carcinoma. <more data available...></td><td>info:pmid/22114688#cont:318, info:pmid/23449973#title:1, info:pmid/20357007#body:187, info:pmid/21610092#cont:291, info:pmid/10801882#body:49, info:pmid/23907942#cont:36</td><td>2</td><td>6</td><td></td></tr>
<tr><td>ST14 ---| apoptosis</td><td>Regulation</td><td>MT-SP1 inhibition may result in the observed inhibition of differentiation and/or increased apoptosis., The data presented in this paper show that Matriptase/MT-SP1 has a pivotal role in thymocyte survival by suppressing apoptosis.</td><td>info:pmid/10500122#body:283, info:pmid/12032844#body:224</td><td>2</td><td>2</td><td>prostate ventral lobe {Organ urn: agi-ncimorgan: C2700158}</td></tr>
<tr><td>FLT1 ---> MMP2</td><td>Regulation</td><td>VEGF has been shown to regulate MMP-2 expression in several cell lines.5 Taken together, these findings suggest that swimming training likely provides these beneficial effects on ischemic revascularization in aged mice through VEGF/Flt-1-mediated MMP-2 activation induced by stimulation of insulin-like growth factor-1/phosphatidylinositol 3-kinase/Akt-dependent hypoxia-induced factor-1a synthesis and prevention of hypoxia-induced factor-1a destabilization.</td><td>info:pmid/20679550#body:139</td><td>2</td><td>1</td><td></td></tr>
<tr><td>FLT1 ---> VIM</td><td>Expression</td><td>Western blotting showed that VEGFR-1 activation led to decreased expression of the epithelial markers E-cadherin and plakoglobin, increased expression of the mesenchymal markers vimentin and N-cadherin, and increased nuclear expression of beta-catenin., Moreover, VEGFR1 activation leads to down-regulation of the expression of E-cadherin and plakoglobin, and to up-regulation of mesenchymal vimentin and N-cadherin.</td><td>info:pmid/16397214#abs:5, info:pmid/20890584#cont:81</td><td>2</td><td>2</td><td>Mesenchyme {Organ urn: agi-ncimorgan: C0162415}</td></tr>
<tr><td>THBS1 ---| ST14</td><td>Expression</td><td>Incubations with Human platelet TSP1 at 100 µg/ml resulted in greater inhibition of degradation of labeled mTSP1 or mouse TSP2. 3T3 cells appeared necrotic after incubation with Human platelet TSP1 at concentrations higher than 150 µg/ml.</td><td>info:pmid/8663244#body:173</td><td>2</td><td>1</td><td></td></tr>
<tr><td>ST14 ---> CLDN7</td><td>Expression</td><td>Alternatively, the expression levels of matriptase may affect the expression levels of claudin-7.</td><td>info:pmid/21310043#cont:323</td><td>2</td><td>1</td><td></td></tr>
<tr><td>PDGFRA ---> epithelial to mesenchymal transition</td><td>Regulation</td><td>We previously showed that there was a relation between WT1 and Pdgfra, which may affect the process of epithelial-to-mesenchymal transformation [7]., In addition, platelet-derived growth factor family receptora was shown to be important for maintenance of epithelial-mesenchymal transition, evasion of apoptosis, and metastasis in transformed mesenchymal murine mammary cells.</td><td>info:pmid/21516490#cont:285, info:pmid/22070644#cont:30</td><td>2</td><td>2</td><td>Mesenchyme {Organ urn: agi-ncimorgan: C0162415}</td></tr>
</table>

HIF1A ---> IGF2BP1	PromoterBinding	Hypoxic stimulation transcriptionally regulates CRD-BP by facilitating the acetylation of histones within the CRD-BP gene and by modulating the extent of HIF1a binding to the CRD-BP promoter.	info:pmid/23038779#abs:4	2	1	
IGF2BP1 ---| cell invasion	Regulation	Here, we show that in both T47D and MDA231 human breast carcinoma cells IMP1/ZBP1 functions to suppress cell invasion.	info:pmid/22266909#abs:5	2	1	
PDGFRA --+> cell invasion	Regulation	Furthermore, suppression of PDGFRA and PLCG1 is involved in miR-218-2-induced inhibition of thyroid cancer cell invasion and growth., (D) Impact of Dyn2 knockdown on PDGFRa-promoted LN444 glioma growth, invasion, cell proliferation and apoptosis *in vivo*.	info:pmid/23720784#cont:190, info:pmid/21996738#cont:292	2	2	Fetus {Organ urn: agi-ncimorgan: C1305737}
HIF1A ---> NQO1	Regulation	Ethanol-induced HO-1 and NQO1 are differentially regulated by HIF-1alpha and Nrf2 to attenuate inflammatory cytokine expression., Ethanol-induced HO-1 and NQO1 are differentially regulated by HIF-1a and Nrf2 to attenuate inflammatory cytokine expression.	info:pmid/20833713#title:1, info:pmid/23294312#cont:1105	2	2	Cerebellum {Organ urn: agi-ncimorgan: C1268981}
IGF2BP1 ---> CDH1	Expression	IMP1/ZBP1 facilitates the localization of E-cadherin and b-actin mRNAs at cell–cell contacts	info:pmid/22266909#cont:85	2	1	
TXNIP ---| cell migration	Regulation	dipeptidyl peptidase-4, KLF4 and thioredoxin interacting protein instead inhibit cell migration and invasion and induce cell cycle arrest [37–39].	info:pmid/22328933#cont:251	2	1	
EGLN3 ---> epithelial to mesenchymal transition	Regulation	In this case, downregulation of PHD3 expression following hypoxia and/or cytokine-mediated epithelial-to-mesenchymal transition may have evolved to disengage PHD3-mediated hydroxylation, and allow for cell cytoskeletal changes necessary for cell motility.	info:pmid/24367580#cont:346	2	1	
HIF1A --+> PDGFRA	PromoterBinding	At first approximation, this would seem contrary to the results showing that hypoxia-inducible factor1a and hypoxia-inducible factor2a specifically regulate PDGFRa and FAK.	info:pmid/23563312#cont:166	2	1	
GLIPR2 --+> cell migration	Regulation	These findings suggest that GLIPR-2 may be involved in proximal tubular cell epithelial-to-mesenchymal transition to promote cell migration.	info:pmid/23516513#cont:296	2	1	
ST14 --+> MMP2	Regulation	Although our study did not determine this as a direct proteolytic activation by matriptase, the presence of matriptase is clearly required for MMP2 activation.	info:pmid/21149451#cont:409	2	1	
EGLN3 ---> cell migration	Regulation	The presence of stress fibres and phosphorylated FAK is a marker of focal adhesions that anchor the F-actin filaments to transmembrane proteins and mediate communication with the cell's environment suggesting that PHD3 as oxygen sensor also influences tumour cell invasion and possibly cell migration with the biological function of escaping hypoxic areas.	info:pmid/20978507#cont:582	2	1	

<table>
<tr><td></td><td></td><td></td><td></td><td></td><td></td><td></td></tr>
<tr><td>NQO1 --+> THBS1</td><td>Regulation</td><td>Several mRNAs, such as those encoding CYP1B1, IL8, neuronal pentraxin 1, plasminogen activator inhibitor 2, PTGS2, NQO1, signal transducer and activator of transcription 1, transforming growth factor, beta-induced, thrombospondin 1, TNFRSF11B, and WNT5A, were induced in both types of cells by the AhR ligands 2,3,7,8- tetrachlorodibenzo- p-dioxin and polychlorinated biphenyl-126, and the fold induction of expression was generally higher in precursors than in adipocytes [see Supplemental Material, ...</td><td>info:pmid/22262711#cont:303</td><td>2</td><td>1</td><td></td></tr>
<tr><td>SOD3 ---> cell invasion</td><td>Regulation</td><td>Therefore, nonmetastatic 67NR cells that express a higher mH2A1.1-to-mH2A1.2 ratio than metastatic 4T1 cells might be able to adapt their genetic programs to redox metabolism by expressing genes such as SOD3, which would in turn affect cell invasiveness.</td><td>info:pmid/23022728#cont:717</td><td>2</td><td>1</td><td></td></tr>
<tr><td>EGLN3 ---> cell invasion</td><td>Regulation</td><td>PHD3 modulates tumour cell growth, invasion and cell morphology</td><td>info:pmid/20978507#cont:195</td><td>2</td><td>1</td><td>Pancreas {Organ urn: agi-ncimorgan: C1278931}</td></tr>
<tr><td>ESRP1 ---> CDH1</td><td>Expression</td><td>Esrp1 silencing resulted in significantly decreased levels of E-cadherin after 3-d silencing (Fig. 5B).</td><td>info:pmid/22585092#cont:137</td><td>2</td><td>1</td><td></td></tr>
<tr><td>CDH1 --+> ESRP1</td><td>Expression</td><td>Interestingly, examination components. of the microarray data (Sup. Table 1) revealed that expression of Upon Slug expression in UM-SCC-38 cells, we observed an ESRP1 and ESRP2 mRNA were decreased in UM-SCC-38/slug expected reduction in E-cadherin expression and a striking loss cells compared to control cells in agreement with the observed of E-cadherin localization of the remaining E-cadherin from alternative splicing of the p120 mRNA.</td><td>info:embase/ 2011455233#cont:113</td><td>2</td><td>1</td><td></td></tr>
<tr><td>KRT14 ---| NQO1</td><td>Regulation</td><td>Relative to normal corneal epithelial proteins, six keratoconus epithelial proteins (lamin-A/C, keratin type I cytoskeletal 14, tubulin beta chain, heat shock cognate 71 kDa protein, keratin type I cytoskeletal 16 and protein S100-A4) exhibited up-regulation and five proteins (transketolase, pyruvate kinase, 14-3-3 sigma isoform, phosphoglycerate kinase 1, and NADPH dehydrogenase (quinone) 1) showed down-regulation.</td><td>info:pmid/21281627#abs:5</td><td>2</td><td>1</td><td>Cornea {Organ urn: agi-ncimorgan: C1550625}</td></tr>
<tr><td>NQO1 ---| KRT14</td><td>Regulation</td><td>In this report, we demonstrate that inactivation of NQO1 gene in mice led to the loss of cytokeratin 14 (K14), an early marker of skin differentiation and thinning of skin epithelium.</td><td>info:pmid/21042282#cont:36</td><td>2</td><td>1</td><td>Skin {Organ urn: agi-ncimorgan: C0684084}</td></tr>
<tr><td>TXNIP ---| VIM</td><td>Regulation</td><td>Consistently, the depletion of E-Cadherin, an epithelial marker, was quickened, and similarly the induction of vimentin, a mesenchymal marker, was elevated in Thioredoxin Binding Protein-2 knockdown-A549 cells (Fig. 6).</td><td>info:pmid/22768160#cont:93</td><td>2</td><td>1</td><td>Mesenchyme {Organ urn: agi-ncimorgan: C0162415}</td></tr>
<tr><td>LCN2 ---- THBS1</td><td>Binding</td><td>DNMT1 is nuclear, but reported to co-localize with annexin V. Lipocalin 2 is decreased by 1.29 fold and is also negatively associated with THBS1, which is increased by 2.13 fold and identified as a binding partner of ELA2.</td><td>info:pmid/21853032#cont:153</td><td>2</td><td>1</td><td>Bone Marrow {Organ urn: agi-ncimorgan: C0005953}</td></tr>
</table>

FLT1 ---> CDH1	Expression	Western blotting showed that VEGFR-1 activation led to decreased expression of the epithelial markers E-cadherin and plakoglobin, increased expression of the mesenchymal markers vimentin and N-cadherin, and increased nuclear expression of beta-catenin., Moreover, VEGFR1 activation leads to down-regulation of the expression of E-cadherin and plakoglobin, and to up-regulation of mesenchymal vimentin and N-cadherin., VEGFR-1 activation led to reduced E-cadherin expression while EMT-associated transcription factors (Snail, Slug, Twist) and mesenchymal markers vimentin and N-cadherin were upregulated. <more data available...>	info:pmid/16397214#abs:5, info:pmid/20890584#cont:81, info:pmid/21492078#cont:110, info:pmid/23776453#cont:595	2	4	Mesenchyme {Organ urn: agi-ncimorgan: C0162415}
WNT4 ---> CDH1	Expression	Further studies found that the expression of cell polarity associated genes (Par6b and E-cadherin) and Wnt signaling genes (Wnt4, Wnt11) were downregulated in Wt1 deficient SCs, and that the expression of Par6b and E-cadherin was regulated by Wnt4.	info:pmid/23935527#abs:8	2	1	Testis {Organ urn: agi-ncimorgan: C1278981}
PDGFRA ---> MMP3	Expression	Our studies show that PDGFR-alpha induced MMP3 gene expression and increased cell proliferation and cell migration upon stimulation by platelet-derived growth factor -AA., We investigated PDGF-alpha receptor - and beta receptor -mediated signaling pathways for the expression of MMP-3 and invasion activity using porcine aortic endothelial cells with stable expression of normal or mutated Platelet-derived growth factor receptors.	info:pmid/14519668#abs:6, info:pmid/12051699#abs:3	2	2	Nervous system {Organ urn: agi-ncimorgan: C0027763}, Aorta {Organ urn: agi-ncimorgan: C1278934}
TXNIP ---| cell invasion	Regulation	VDUP1 suppressed cell invasiveness and tumor metastasis, which were also recovered by blocking of nuclear export.	info:pmid/18062927#abs:5	2	1	
EGLN3 ---| HIF1A	ProtModification	Consequently, in hypoxia Siah determines the availability of PHD1/3, which otherwise modify HIF-1alpha to enable its association-dependent degradation by pVHL., Although PHD1 and PHD3 can hydroxylate HIF-1a *in vitro*, HIF does not appear to be their physiological target in the cell (3)., During normoxia, the HIF-1alpha subunit is hydroxylated on two prolines by three recently identified prolyl hydroxylases (PHD1, PHD2, and PHD3) (13–17)., HIF-1a can also be regulated at the protein level by the oxygen-dependent hydroxylation of HIF-1a by specific prolyl hydroxylases (PHD1-PHD3). <more data available...>	info:pmid/15492505#abs:5, info:pmid/18463229#body:64, info:pmid/12888568#body:69, info:pmid/18202012#body:72, info:pmid/12615973#body:63, info:pmid/16815840#body:175	2	6	
